# Ge=B π‐Bonding: Synthesis and Reversible [2+2] Cycloaddition of Germaborenes

**DOI:** 10.1002/anie.201914608

**Published:** 2020-01-07

**Authors:** Dominik Raiser, Christian P. Sindlinger, Hartmut Schubert, Lars Wesemann

**Affiliations:** ^1^ Institut für Anorganische Chemie Eberhard Karls Universität Tübingen Auf der Morgenstelle 18 72076 Tübingen Germany; ^2^ Institut für Anorganische Chemie Georg August Universität Göttingen Tammannstr. 4 37077 Göttingen Germany

**Keywords:** [2+2] cycloaddition, borylene, germylene, photochemistry, π-bonding

## Abstract

Phosphine‐stabilized germaborenes featuring an unprecedented Ge=B double bond with short B⋅⋅⋅Ge contacts of 1.886(2) (**4**) and 1.895(3) Å (**5**) were synthesized starting from an intramolecular germylene–phosphine Lewis pair (**1**). After oxidative addition of boron trihalides BX_3_ (X=Cl, Br), the addition products were reduced with magnesium and catalytic amounts of anthracene to give the borylene derivatives in yields of 78 % (**4**) and 57 % (**5**). These halide‐substituted germaborenes were characterized by single‐crystal structure analysis, and the electronic structures were studied by quantum‐chemical calculations. According to an NBO NRT analysis, the dominating Lewis structure contains a Ge=B double bond. The germaborenes undergo a reversible, photochemically initiated [2+2] cycloaddition with the phenyl moiety of a terphenyl substituent at room temperature, forming a complex heterocyclic structure with Ge^IV^ in a strongly distorted coordination environment.

## Graphical abstract

**Germaborenes** with the first authentic Ge=B double bond were synthesized. Upon photoactivation, germaborenes undergo a reversible [2+2] cycloaddition reaction with an arene moiety.

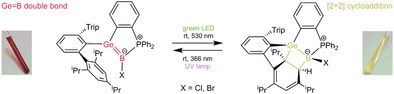

The chemistry of boron–element compounds containing B−E multiple bonds is a challenging field of research.^1^ Currently, the chemistry of B−B double and triple bonds is in the focus of interest.1k In terms of B=E double bonds with elements of Group 14, compounds exhibiting a double bond between boron and carbon were reported more than thirty years ago by Berndt, Nöth and Paetzold et al. (Scheme 1, **A**–**C**).1p–1r

**Scheme 1:**
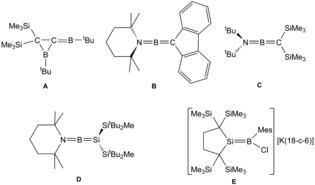
Examples of B=E double bonds (E=C, Si).

A double bond between boron and silicon was reported in 2006 (Scheme 1, **D**).1o Sekiguchi and co‐workers reacted a 1,1‐dilithiosilane [Li_2_Si(Si*t*Bu_2_Me)_2_] with 2,2,6,6‐tetramethylpiperidinedichloroborane and isolated the low‐valent boron compound as yellow crystals.1o The borasilene features a Si=B double bond of 1.8379(17) Å. A chloride adduct of a borasilene (Scheme 1, **E**) was isolated after reduction of the addition product between a cyclic dialkylsilylene and dichloromesitylborane. In the chloride adduct, the double bond between boron and silicon [1.859(2) Å] is only slightly longer than in example **D**.1s A carbene adduct of a borasilene exhibiting a longer Si−B distance of 1.990(2) Å was reported by Inoue and co‐workers.1x

We have recently reported the synthesis of a phosphine‐stabilized digermavinylidene.^2^ Its synthesis started from an intramolecular Lewis pair between a germylene and a phosphine (**1**).^3^ After addition of germanium dichloride, the addition product was reduced with a strong reducing agent such as {(^Mes^Nacnac)Mg}_2_, and the digermavinylidene was isolated in 52 % yield [^Mes^Nacnac={[(Mes)NC(Me)]_2_CH}^−^, Mes=2,4,6‐Me_3_C_6_H_2_].^4^

Herein, we report on the synthesis of phosphine‐stabilized germaborenes.1ag The intramolecular Lewis pair **1** was reacted with boron trihalides, and the oxidative addition products **2** and **3** were isolated in high yield and characterized (Scheme 2). In the ^11^B NMR spectrum, the tetracoordinated boron atoms of adducts **2** and **3** lead to resonances at −0.3 (**2**) and −13.1 ppm (**3**). The resonances of the phosphorus atoms were found at 3.9 (**2**) and 6.8 ppm (**3**) in the ^31^P NMR spectrum. These ^11^B and ^31^P NMR resonances can be compared with signals of phosphine adducts of boron trihalides or organoboron dihalides.^5^

**Scheme 2:**
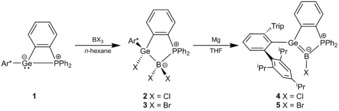
Synthesis of germaborenes **4** and **5**. Ar*=2,6‐Trip_2_C_6_H_3_, Trip=2,4,6‐triisopropylphenyl.

In Figure 1, the molecular structure of the addition product **2** in the solid state is depicted. The molecular structure of the BBr_3_ addition product **3**, including crystallographic details, is presented in the Supporting Information. The Ge−B bond lengths of **2** [2.095(5) Å] and **3** [2.089(2) Å] lie in the range of interatomic distances found for B−Ge single bonds [2.033(6)–2.260(13) Å].^6^ The B−P distances in **2** [2.017(5) Å] and **3** [1.979(2) Å] are closely related to B−P distances found in phosphine adducts of boron trihalides [1.952(2)–1.970(2) Å].5c, 5d

**Figure 1:**
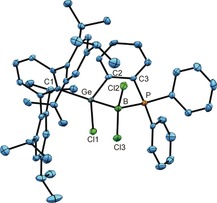
ORTEP^7^ of the molecular structure of **2**. Ellipsoids set at 50 % probability. Hydrogen atoms are omitted for clarity. Interatomic distances [Å] and angles [°]: Ge–B 2.095(5), Ge–Cl1 2.2059(10), Ge–C1 1.990(4), Ge–C2 1.968(4), B–P 2.017(5), B–Cl2 1.845(5), B–Cl3 1.831(4), C2–C3 1.410(5); Ge‐B‐P 95.9(2), C2‐Ge‐B 96.5(2), B‐P‐C3 101.6(2), P‐C3‐C2 115.9(3), C3‐C2‐Ge 115.3(3), B‐Ge‐Cl1 98.3(1), B‐Ge‐C2 96.5(2), P‐B‐Cl2 102.4(2), P‐B‐Cl3 114.1(2).

To reduce the addition products **2** and **3**, reactions with magnesium were studied. The best results were obtained after activation of the magnesium with dibromoethane and addition of catalytic amounts of anthracene.^8^ Reductions were carried out in tetrahydrofuran at ambient temperature (Scheme 2). In the course of the reduction the colorless solution turned deep red, and the reduction products **4** and **5** were isolated as single crystals from a pentane solution. The lowest‐energy absorption bands observed in the UV/Vis spectra were located at *λ*_max_(**4**)=528 nm and *λ*_max_(**5**)=520 nm. The molecular structures in the solid state of **4** and **5** were determined by single‐crystal X‐ray diffraction, and in Figure 2, an ORTEP of **4** is shown.

**Figure 2:**
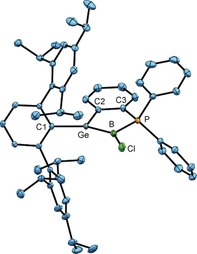
ORTEP of the molecular structure of **4**. Ellipsoids set at 50 % probability. Hydrogen atoms are omitted for clarity. Interatomic distances [Å] and angles [°]: Ge–B 1.886(2), Ge–C1 1.944(1), Ge–C2 1.948(1), B–P 1.888(2), B–Cl 1.786(2), C2–C3 1.411(2), C3–P 1.811(1); C1‐Ge‐C2 112.9(1), C2‐Ge‐B 102.0(1), C1‐Ge‐B 144.7(1), Ge‐B‐P 103.1(1), B‐P‐C3 106.4(1), P‐C3‐C2 114.6(2), C3‐C2‐Ge 113.8(1), Cl‐B‐Ge 138.7(1), Cl‐B‐P 118.2(1).

The Ge−B bond lengths of 1.886(2) (**4**) and 1.895(3) Å (**5**) are short distances between these elements and point towards a double bond. Recently, Apeloig, Driess, and co‐workers have published a borylsilylgermylene stabilized by a silylene donor.^9^ For this molecule, resonance structures with partial Ge=B character were discussed, and crystal‐structure analysis gave a bond length of 1.971(2) Å. In the molecular structures of germaborenes **4** and **5**, the observed short Ge−B interatomic distances go along with trigonal‐planar coordination at these atoms, which is manifested by the sum of the bond angles around the Ge and B atoms [**4**: Σ angles at Ge 359.6°, B 359.8°; **5**: Ge 359.6°, B 360.0°]. This gives rise to a characterization of this bond as a classic double bond. In comparison to the starting materials **2** and **3**, we found slightly shorter B−P distances in the reduction products [**4**: 1.888(2), **5**: 1.878(3) Å]. The ^31^P NMR signals of **4** and **5** show only slight changes in chemical shift in comparison to the starting materials **2** and **3**. In the ^11^B NMR spectrum, the resonances for the reduction products were found as doublets at lower field at 17.3 ppm, ^1^ *J*_PB_=132 Hz (**4**) and 10.3 ppm, ^1^ *J*_PB_=134 Hz (**5**), corroborating the coordination number three; the coupling with the neighboring phosphorus atom was resolved as a doublet. To evaluate the electronic structures of the germaborenes **4** and **5**, quantum‐chemical calculations were carried out (see the Supporting Information).^10^ The density functional theory optimized geometries of both compounds **4** and **5** are in good agreement with the solid‐state structures [Ge−B distance in **4**: exp. 1.886(2) Å, calc. 1.910 Å; **5**: exp. 1.895(3) Å, calc. 1.904 Å].

In Figure 3, the HOMO, representing the π‐bond between B and Ge of the bromo‐substituted germaborene **5**, is shown. A natural bond orbital (NBO) analysis reveals an occupancy of 1.94 electrons for the Ge−B σ‐bond and of 1.67 electrons for the π‐bond.^12^ Both bonds are slightly polarized: σ‐bond Ge(44.2 %)−B(55.8 %) and π‐bond Ge(42.3 %)−B(57.8 %). This leads to a Wiberg bond index (WBI) of 1.51, indicating a covalent Ge−B bond in line with Ge−B multiple bond character. NBO and NRT (natural resonance theory) analyses on model compounds 4* and 5*, where Ar* and Ph have been replaced by Me, were also performed. Among all of the suggested structures, the dominating Lewis structure contains a Ge=B double bond as depicted in Figure 3. Similar to the results for **5**, the B−Ge σ‐bonds reveal an occupancy of 1.97 (4*) and 1.96 (5*) electrons, and the occupancies of the B−Ge π‐bonds are 1.77 (4*) and 1.78 (5*) electrons, to give a natural B−Ge bond order of 1.73 (4* and 5*). A resonance structure of notable contribution considers π‐bonding from a halide lone pair that is donated to boron (see the Supporting Information). However, no relevant contribution of >1 % was obtained for a structure that contains a boron‐based lone pair as a description of base‐stabilized borylenes would implement. This further corroborates the proposed efficient orbital overlap allowing for authentic Ge=B π‐bonding.

**Figure 3:**
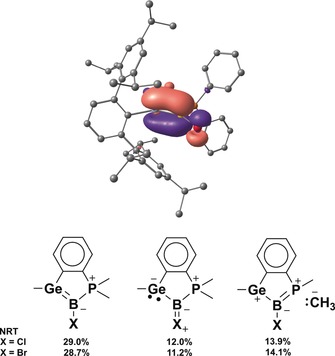
HOMO (top) of **5**. Δ*E*(HOMO–LUMO)=1.572 eV.^11^ Results of the NBO NRT calculations (substituents Ar* and Ph replaced by Me groups).

Both germaborenes **4** and **5** were exposed to the light of a mercury vapor lamp. Upon using this lamp with a relatively broad spectral range, only mixtures of the starting germaborene (**4**, **5**) and the product of cycloaddition (**6**, **7**) were isolated (**4**/**6**: 30/70 %; **5**/**7**: 10/90 %). Using a light source with a wavelength of 530 nm, which is close to that of the lowest‐energy absorption band, quantitative formation of the [2+2] cycloaddition products **6** and **7** was observed by NMR spectroscopy (Scheme 3). A computationally predicted absorption spectrum by TDDFT of **4** is in good agreement with the experimental spectrum.^13^ The nature of the lowest‐energy excitation around *λ*=520 nm is dominated by a transition from the Ge=B π‐orbital into the π‐system of the phenylidene moiety (see the Supporting Information). Upon heating compounds **4** and **5** under the exclusion of daylight for 68 h at 60 °C, no transformation of either compound into the corresponding cycloaddition product was observed by NMR spectroscopy.

**Scheme 3:**
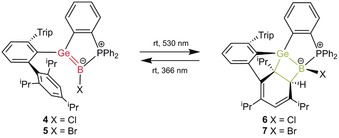
Reversible [2+2] cycloaddition of **4** and **5**.

In the ^11^B NMR spectrum and in the ^31^P NMR spectrum, new resonances for compounds **6** and **7** were seen. The ^11^B NMR signals were observed as singlets at −4.5 (**6**) and −10.1 ppm (**7**), indicating tetracoordination at the boron atoms. However, coupling with the neighboring phosphorus atom was not resolved. Crystals suitable for X‐ray diffraction were obtained for both cycloaddition products from hexane or benzene solutions. In both cases, four stereogenic tetracoordinate atoms Ge, B, C4, and C5 are formed upon ring formation. Compound **7** crystallizes in the space group *P*2_1_, and we observed only one enantiomer by single‐crystal X‐ray diffraction, whereas compound **6** crystallizes in the centrosymmetric space group *P*2_1_/*n*.^14^ In Figure 4 an ORTEP of the molecular structure of **6** in the solid state is depicted. The Ge=B double bond of the germaborene has reacted with the double bond of a Trip moiety of a terphenyl substituent giving rise to a complex polyheterocyclic system.

**Figure 4:**
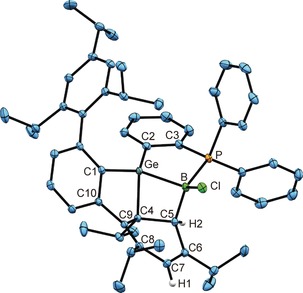
ORTEP of the molecular structure of **6**. Ellipsoids set at 50 % probability. Hydrogen atoms except for H1 and H2 are omitted for clarity. Interatomic distances [Å] and angles [°]: Ge–B 2.1229(16), B–P 1.9766(16), P–C3 1.8227(14), C2–C3 1.407(2), Ge–C2 1.9509(14), Ge–C1 1.9393(14), Ge–C4 1.9920(14), B–C5 1.663(2), B–Cl 1.8395(16), C4–C5 1.5622(19), C4–C9 1.5442(19), C9–C10 1.4952(19), C1–C10 1.4207(19), C5–C6 1.5098(19), C6–C7 1.342(2), C7–C8 1.471(2), C8–C9 1.3588(19); C1‐Ge‐C2 127.7(1), C1‐Ge‐B 127.9(1), C1‐Ge‐C4 93.0(1), C2‐Ge‐B 102.2(1), C4‐Ge‐B 74.4(1), C2‐Ge‐C4 116.8(1), C5‐B‐P 114.8(1), Ge‐B‐P 96.2(1), C5‐B‐Ge 86.3(1), Cl‐B‐Ge 124.1(1), Cl‐B‐P 113.6(1), Cl‐B‐C5 117.9(1), B‐P‐C3 106.2(1), C3‐C2‐Ge 112.5(1), P‐C3‐C2 118.7(1), C10‐C1‐Ge 105.2(1), C4‐C9‐C10 114.2(1), C9‐C10‐C1 115.4(1), Ge‐C4‐C9 95.0(1), C4‐C5‐C6 111.1(1), C4‐C5‐B 101.1(1), C5‐C6‐C7 117.7(1), C6‐C7‐C8 123.2(1), C7‐C8‐C9 118.7(1), C8‐C9‐C4 119.1(1).

The Ge−B bond of **6** is expectedly elongated upon [2+2] addition to 2.1229(16) Å, which can be compared with the Ge−B single bonds found in **2** and **3**. The length of the bond between C4 and C5 of 1.5622(19) Å is in line with a single bond. Double bonds between C6/C7 [1.342(2) Å] and C8/C9 [1.3588(19) Å] were found for the cyclohexadiene moiety. Upon formation of the four‐membered cycle, the germanium atom adopts a distorted trigonal (C1, C2, B) pyramidal (C4) geometry. The sum of the bond angles around the germanium atom for the substituents C1, C2, and B is 358.7°. Considering the initial observation of product mixtures with mercury lamps, we probed potential reversibility by exposure of **6** and **7** to light of shorter wavelength. Indeed, the reversibility of the cycloaddition was confirmed upon irradiation of the addition products with light of lower wavelength (366 nm). After irradiation for three days at room temperature, both addition products had been reconverted into the germaborenes (Scheme 3; see the Supporting Information for NMR spectra). The calculated TDDFT spectrum of **6** is also in good agreement with the experimental spectra, and the lowest‐energy transitions predicted to occur around 320 nm involve excitations from HOMO and HOMO−1, which contain contributions of the cyclohexadiene π‐system and the C−Ge/C−B bonds (HOMO) and the Ge−B single bond (HOMO−1), respectively (see the Supporting Information).

Otherwise, when stored under the exclusion of daylight, compounds **6** and **7** are stable. Even heating up to 60 °C in benzene solution does not result in any reconversion of **6** or **7**. Cycloaddition reactions between alkenes and arenes are widely used in organic synthesis because of their high efficiency and selective stereochemistry.^15^ In the case of main group element chemistry, some rare examples of [4+2] cycloaddition reactions between unsaturated main group element compounds and arenes have been published. Recently, Stephan and co‐workers reported a reversible intramolecular [4+2] cycloaddition of a phosphaalkene with an arene ring.^16^ Sakurai et al. published the [4+2] addition of a disilene.^17^ [4+2] Cycloaddition reactions with an unsaturated dialuminium species were presented by Power et al. and Tokitoh et al.^18^ Furthermore, Xie and co‐workers generated 1,3‐dehydro‐*o*‐carborane and isolated the products of a [4+2] cycloaddition.^19^ The high reactivity of unsaturated element–boron compounds, B=Si, was documented by Iwamoto and co‐workers with the CH activation of borasilene.1s Cycloaddition reactions of alkynes with homodinuclear multiple bonds of Group 13 and 14 element compounds have been reported for B, Al, Ga, Si, Ge, and Sn.18b, 20 Germaborenes **4** and **5** undergo a reversible [2+2] cycloaddition with an arene moiety upon photoactivation. In main group element chemistry, the reported reaction is a rare example of this type of cycloaddition.

To conclude, following a reaction sequence of oxidative addition and reduction, halide‐substituted germaborenes with the first authentic Ge=B double bond were synthesized in a straightforward fashion starting from an intramolecular, stabilized germylene–phosphine Lewis pair. The electronic structure of the Ge=B double bond was studied by DFT calculations and analyzed by the NBO NRT method. The high reactivity of the Ge=B double bond was manifested by the reversible intramolecular [2+2] cycloaddition with a C=C double bond of a phenyl substituent.

## Conflict of interest

The authors declare no conflict of interest.

## Supporting information

As a service to our authors and readers, this journal provides supporting information supplied by the authors. Such materials are peer reviewed and may be re‐organized for online delivery, but are not copy‐edited or typeset. Technical support issues arising from supporting information (other than missing files) should be addressed to the authors.

Supplementary
