# Supplementary material for: Ge=B π‐Bonding: Synthesis and Reversible [2+2] Cycloaddition of Germaborenes
Source: Angew Chem Int Ed Engl. 2020 Jan 7;59(8):3151–5. doi: 10.1002/anie.201914608 (PMC7028040; doi:10.1002/anie.201914608)
Supplement: Supplementary file 1 — Supplementary [file ANIE-59-3151-s001.pdf]

Angewandte Chemie
Eine Zeitschrift der Gesellschaft Deutscher Chemiker

Supporting Information

Ge=B π-Bonding: Synthesis and Reversible [2+2] Cycloaddition of Germaborenes

*Dominik Raiser, Christian P. Sindlinger, Hartmut Schubert, and Lars Wesemann**

anie_201914608_sm_miscellaneous_information.pdf

Supporting Information

Content

Experimental Details S2

General Information S2

Syntheses S2

NMR spectroscopy S7

NMR spectra of compound **2**. S7

NMR spectra of compound **3**. S11

NMR spectra of compound **4**. S15

NMR spectra of compound **5**. S19

NMR spectra of compound **6**. S23

NMR spectra of compound **7**. S27

Crystallography S34

Computational Details S38

Structure Optimisation, NBO, NRT analyses S38

TDDFT calculations S50

References S61

Experimental Details

General Information

All manipulations were carried out under argon atmosphere using standard Schlenk techniques or an MBraun Glovebox. Benzene and toluene were distilled from sodium. Hexane and pentane were obtained from a MBRAUN solvent purification system and degassed by three times freeze pump thaw. Benzene-d_6 and toluene-d_8 were distilled from sodium and stored over molecular sieve. Terphenyl-Ge(II) chloride (Ar*GeCl), (o-PPh_2)C_6H_4GeAr* **1** and Mg anthracene were prepared according to a literature procedure.[1] Furthermore, chemicals were purchased commercially and used as received. Elemental analysis was performed by the Institut für Anorganische Chemie, Universität Tübingen using a Vario MICRO EL analyser.

NMR spectra were recorded with either a Bruker Avance III HD 300 NanoBay spectrometer equipped with a 5 mm BBFO probe head and operating at 300.13 (^{1}H), 75.47 (^{13}C), 121.49 (^{31}P) and 96.29 (^{11}B) MHz, a Bruker AvanceII+400 NMR spectrometer equipped with a 5 mm QNP (quad nucleus probe) head and operating at 400.13 (^{1}H), 100.62 (^{13}C), 161.97 (^{31}P) MHz, a Bruker AVII+ 500 NMR spectrometer with a variable temperature set up and a 5 mm TBO probe head and operating at 500.13 (^{1}H), 125.76 (^{13}C), 202.47 (^{31}P) and 160.46 (^{11}B) MHz, a Bruker Avance III HDX 600 NMR spectrometer with a 5 mm Prodigy BBO cryo probe head operating at 600.13 (^{1}H), 150.90 (^{13}C), 242.94 (^{31}P) and 192.55 (^{11}B) MHz or a Bruker Avance III HDX 700 NMR spectrometer with a 5 mm Prodigy TCI cryo probe head operating at 700.29 (^{1}H), 176.10 (^{13}C) MHz. Chemical shifts are reported in δ values in ppm relative to external TMS (^{1}H, ^{13}C) and), 85% aqueous H_3PO_4 (^{31}P) referenced in most cases on the residual proton signal of the solvent C_6D_6 (^{1}H 7.15 ppm; ^{13}C 128.0 ppm). ^{31}P- as well as ^{1}H and ^{13}C-spectra in toluene-d_8, benzene-d_6 were referenced using the chemical shift of the solvent ^{2}H resonance frequency and Ξ = 25.145020% for ^{13}C, Ξ = 40.480742% for ^{31}P, Ξ = 32.083974% ^{11}B.[2] The multiplicity of the signals is abbreviated as s = singlet, d = doublet, t = triplet, quint = quintet, sept = septet and m = multiplet or unresolved. The proton and carbon signals were assigned by detailed analysis of ^{1}H, ^{13}C{^{1}H}, ^{1}H-^{1}H COSY, ^{1}H-^{13}C HSQC, ^{1}H-^{13}C HMBC and ^{13}C{^{1}H} DEPT 135 spectra. Selected 1D-NMR spectra of the compounds and mixtures can be found in the Supporting Information.

UV/vis measurements were performed on a PerkinElmer Lambda 35 instrument.

Syntheses

Elemental analyses were performed with the crystalline substances.

Synthesis of (o-PPh_2)C_6H_4Ge(Cl){Ar*}(BCl_2) (2). (o-PPh_2)C_6H_4GeAr* **1** (200 mg, 245 µmol, 1.00 eq) was dissolved in hexane (10 mL) and added dropwise to a solution of $Me_2S{\cdot}BCl_3$ (44.0 mg, 245 µmol, 1.00 eq) in hexane (8 mL). After stirring over night the orange reaction mixture turned to a colorless suspension. The product **2** was filtered off and washed with cold hexane (1 mL). Further batches of product **2** were received by crystallization from hexane solution at −40 °C (combined yield: 214 mg, 201 µmol, 98%). Colorless single crystals suitable for X-ray crystallography were obtained from a concentrated toluene solution with ten drops of *n*-hexane at −40 °C. Analytical data: **^{1}H NMR** (400.11 MHz, 298.2 K, C_6D_6): δ (ppm) 7.62-7.55 (m, 2 H, C_6H_5), 7.50-7.44 (m, 2 H, C_6H_5), 7.28 (d, 2 H, *m*-C_6H_2, $^4J_{HH}$ = 1.7 Hz), 7.20 (d, 2 H, *m*-GeC_6H_3 $^3J_{HH}$ = 7.3 Hz), 7.14 (s, 2 H, *m*-C_6H_2, overlapping solvent signal), 7.10 (t, 1 H, *p*-GeC_6H_3, $^3J_{HH}$ = 7.5 Hz), 7.03 (t, 1 H, *6*-GeC_6H_4, $^3J_{HH}$ = 8.2 Hz), 7.00-6.95 (m, 2 H, *4*-GeC_6H_4, C_6H_5), 6.94-6.86 (m, 3 H, *3*-GeC_6H_4, C_6H_5), 6.82-6.76 (m, 1 H, *5*-GeC_6H_4), 6.76-6.67 (m, 3 H, C_6H_5), 3.42 (sept., 2 H, *o*-C*H*(CH_3)$_2$, $^3J_{HH}$ = 6.6 Hz), 3.17 (sept., 2 H, *o*-C*H*(CH_3)$_2$, $^3J_{HH}$ = 6.7 Hz), 2.87 (sept., 2 H, *p*-C*H*(CH_3)$_2$, $^3J_{HH}$ = 6.9 Hz), 1.44 (d, 6 H, *o*-CH(CH_3)$_2$, $^3J_{HH}$ = 6.8 Hz), 1.33 (d, 6 H, *o*-CH(CH_3)$_2$, $^3J_{HH}$ = 6.5 Hz), 1.29 (d, 6 H, *p*-CH(CH_3)$_2$, $^3J_{HH}$ = 6.8 Hz), 1.28 (d, 6 H, *p*-CH(CH_3)$_2$, $^3J_{HH}$ = 6.8 Hz), 1.20 (d,

6 H, *o*-CH(CH_3)$_2$, $^3J_{HH}$ = 6.7 Hz), 1.11 (d, 6 H, *o*-CH(CH_3)$_2$, $^3J_{HH}$ = 6.8 Hz). **^{13}C{^{1}H} NMR** (100.06 MHz, 298.2 K, C_6D_6): δ (ppm) 155.4 (d, *1*-GeC_6H_4 $^1J_{PC}$ = 22.6 Hz), 148.5 (s, *p*-C_6H_2), 147.5 (s, *o*-C_6H_2), 147.4 (s, *o*-GeC_6H_3), 147.2 (s, *o*-C_6H_2), 139.4 (s, *i*-C_6H_2), 139.0 (d, *i*-GeC_6H_3, $^4J_{PC}$ = 16.0 Hz), 136.2 (d, *3*-GeC_6H_4, $^2J_{PC}$ = 11.8 Hz), 134.4 (d, *o*-PC_6H_5, $^2J_{PC}$ = 8.0 Hz), 134.1 (d, *o*-PC_6H_5, $^2J_{PC}$ = 8.5 Hz), 133.2 (d, *6*-GeC_6H_4, $^3J_{PC}$ = 4.2 Hz), 132.2 (s, *m*-GeC_6H_3), 131.5 (d, *4*-GeC_6H_4, $^3J_{PC}$ = 2.8 Hz), 131.4 (d, *p*-PC_6H_5, $^4J_{PC}$ = 2.8 Hz), 131.3 (d, *p*-PC_6H_5, $^4J_{PC}$ = 2.4 Hz), 129.6 (d, *2*-GeC_6H_4, $^1J_{PC}$ = 70.6 Hz), 128.7 (d, *5*-GeC_6H_4, $^4J_{PC}$ = 8.5 Hz), 128.2 (d, *m*-PC_6H_5, $^3J_{PC}$ = 3.8 Hz), 128.1 (d, *m*-PC_6H_5, $^3J_{PC}$ = 3.8 Hz), 127.7 (s, *p*-GeC_6H_3), 125.5 (d, *i*-PC_6H_5, $^4J_{PC}$ = 61.7 Hz), 123.2 (d, *i*-PC_6H_5, $^4J_{PC}$ = 62.2 Hz), 121.4 (s, *m*-C_6H_2), 121.2 (s, *m*-C_6H_2), 34.5 (s, *p*-CH(CH_3)$_2$), 31.1 (s, *o*-CH(CH_3)$_2$), 31.0 (s, *o*-CH(CH_3)$_2$), 26.1 (s, *o*-CH(CH_3)$_2$), 25.4 (s, *o*-CH(CH_3)$_2$), 24.2 (s, *p*-CH(CH_3)$_2$), 24.1 (s, *p*-CH(CH_3)$_2$), 23.6 (s, *o*-CH(CH_3)$_2$), 23.2 (s, *o*-CH(CH_3)$_2$). **^{31}P{^{1}H} NMR** (161.96 MHz, 298.2 K, C_6D_6) δ (ppm) 3.85 (br, 230Hz hw, s). **^{11}B{^{1}H} NMR** (96.29 MHz, 298.2 K, C_6D_6) δ (ppm) −0.5 (br, 300 Hz hw, s). **Anal.** Calcd. (%) for $C_{54}H_{63}BCl_3GeP$: C 69.53, H 6.81; found: C 69.04, H 6.47.

Synthesis of (o-PPh$_2$)C$_6$H$_4$Ge(Br){Ar*}(BBr$_2$) (3). (o-PPh$_2$)C_6H_4GeAr* **1** (200 mg, 245 µmol, 1.00 eq) was dissolved in hexane (10 mL) and added dropwise to a solution of Me_2S·BBr_3 (76.7 mg, 245 µmol, 1.00 eq) in hexane (8 mL). After stirring over night the orange reaction mixture turned to a colorless suspension. The product **3** was filtered off and washed with cold hexane (1 mL). Further batches of product **3** were obtained by crystallization from the hexane solution at −40 °C (214 mg, 201 µmol, 82%). Colorless single crystals suitable for X-ray crystallography were obtained from a concentrated toluene solution with ten drops of *n*-hexane at −40 °C. Analytical data: **^{1}H NMR** (300.13 MHz, 298.2 K, C_6D_6): δ (ppm) 7.74-7.63 (m, 4 H, C_6H_5), 7.39 (s, 2 H, *m*-C_6H_2), 7.34-7.16 (br s, 2 H, *m*-GeC_6H_3), 7.12-6.94 (m, 7 H, C_6H_5, *p*-GeC_6H_3, *m*-C_6H_2, GeC_6H_4), 6.91-6.84 (m, 1 H, GeC_6H_4), 6.77-6.65 (m, 4 H, C_6H_5, GeC_6H_4), 6.56-6.45 (m, 1 H, GeC_6H_4), 3.53 (br s, 2 H, *o*-C*H*(CH_3)$_2$), 3.31 (br s, 2 H, *o*-C*H*(CH_3)$_2$), 2.91 (sept., 2 H, *p*-C*H*(CH_3)$_2$, $^3J_{HH}$ = 6.9 Hz), 1.71 (d, 6 H, *o*-CH(CH_3)$_2$, $^3J_{HH}$ = 6.9 Hz), 1.34 (d, 6 H, *p*-CH(CH_3)$_2$, $^3J_{HH}$ = 6.9 Hz), 1.33 (d, 6 H, *p*-CH(CH_3)$_2$, $^3J_{HH}$ = 6.9 Hz), 1.40-1.17 (br s, 6 H, *o*-CH(CH_3)$_2$), 1.22 (d, 6 H, *o*-CH(CH_3)$_2$, $^3J_{HH}$ = 6.7 Hz), 1.22 (d, 6 H, *o*-CH(CH_3)$_2$, $^3J_{HH}$ = 6.7 Hz). **^{13}C{^{1}H} NMR** (125.77 MHz, 263.2 K, THF-d$_8$): δ (ppm) 154.2 (d, *1*-GeC_6H_4, $^2J_{PC}$ = 22.6 Hz), 148.9 (s, *p*-C_6H_2), 147.8 (s, *o*-C_6H_2), 147.6 (s, *o*-C_6H_2), 147.1 (s, *o*-GeC_6H_3), 140.7 (s, *i*-C_6H_2), 138.2 (s, *i*-GeC_6H_3), 137.8 (d, GeC_6H_4, J_{PC} = 13.4 Hz), 137.1 (d, GeC_6H_4, J_{PC} = 11.0 Hz), 134.6 (d, PC_6H_5, J_{PC} = 9.5 Hz), 134.1 (d, PC_6H_5, J_{PC} = 7.7 Hz), 133.7 (s, GeC_6H_4), 133.1 (s, GeC_6H_4), 132.2 (s, PC_6H_5), 131.6 (s, PC_6H_5), 128.8-128.4 (m, GeC_6H_3, GeC_6H_4, PC_6H_5), 128.2 (d, PC_6H_5, J_{PC} = 11.0 Hz), 127.4-126.7 (m, PC_6H_5), 121.2 (d, *i*-PC_6H_5, $^1J_{PC}$ = 69.5 Hz), 121.5 (s, *m*-C_6H_2), 121.2 (s, *m*-C_6H_2), 120.3 (s, *m*-C_6H_2), 34.6 (s, *p*-CH(CH_3)$_2$), 34.4 (s, *p*-CH(CH_3)$_2$), 31.3 (s, *o*-CH(CH_3)$_2$), 30.5 (s, *o*-CH(CH_3)$_2$), 27.8 (s, *o*-CH(CH_3)$_2$), 24.6 (s, *o*-CH(CH_3)$_2$), 24.4 (s, *o*-CH(CH_3)$_2$), 23.7 (s, *p*-CH(CH_3)$_2$), 23.6 (s, *p*-CH(CH_3)$_2$), 23.5 (s, *o*-CH(CH_3)$_2$), 22.7 (s, *o*-CH(CH_3)$_2$), 21.9 (s, *o*-CH(CH_3)$_2$). **^{31}P{^{1}H} NMR** (161.96 MHz, 298.2 K, C_6D_6) δ (ppm) 6.84 (br, 256 Hz hw, s). **^{11}B{^{1}H} NMR** (96.29 MHz, 298.2 K, C_6D_6) δ (ppm) −13.1 (br, 392 Hz hw, s). **Anal.** Calcd. (%) for $C_{54}H_{63}BBr_3GeP$: C 60.83, H 5.96; found: C 60.99, H 6.24.

Synthesis of (*o*-PPh$_2$)C$_6$H$_4$(Ar*)GeBCl (4). Magnesium (33.9 mg, 1.39 mmol, 10.0 eq) and two drops of dibromoethane were stirred in tetrahydrofuran (2 mL). After a few minutes anthracene (2.48 mg, 13.9 µmol, 0.10 eq) was added and the reaction mixture was stirred for five minutes followed by addition of (*o*-PPh$_2$)C_6H_4(Ar*)Ge(Cl)BCl_2 **2** (130 mg, 139 µmol, 1.00 eq) dissolved in tetrahydrofuran (5mL). During the reduction the color of the solution turned from colorless to deep red. After four hours the solvent was removed *in vacuo*. After extraction with diethylether (2 mL) and pentane (2 mL) product **4** was obtained as a red powder (94.0 mg, 109 µmol, 78%). Red single crystals of **4** suitable for X-ray crystallography were obtained from a concentrated *n*-pentane solution at −40 °C. Analytical data: **^{1}H NMR** (400.11 MHz, 298.2 K, C_6D_6): δ (ppm) 7.50-7.39 (m, 6 H, C_6H_5, *m*-GeC_6H_3), 7.10 (dd, 1 H, *p*-GeC_6H_3, $^3J_{HH}$ = 8.2 Hz, $^3J_{HH}$ = 8.2 Hz), 7.26-7.21 (m, 1 H, *6*-GeC_6H_4), 7.14 (s, 4 H, *m*-C_6H_2, overlapping

solvent signal), 7.02-6.90 (m, 7 H, *5*-GeC$_6$*H*$_4$, C$_6$*H*$_5$), 6.73-6.86 (m, 1 H, *3*-GeC$_6$*H*$_4$), 6.58-6.53 (m, 1 H, *4*-GeC$_6$*H*$_4$), 3.23 (sept., 4 H, *o*-C*H*(CH$_3$)$_2$, $^3J_{HH}$ = 6.7 Hz), 2.80 (sept., 2 H, *p*-C*H*(CH$_3$)$_2$, $^3J_{HH}$ = 6.9 Hz), 1.35 (d, 12 H, *o*-CH(C*H*$_3$)$_2$, $^3J_{HH}$ = 6.8 Hz), 1.19 (d, 12 H, *p*-CH(C*H*$_3$)$_2$, $^3J_{HH}$ = 6.8 Hz). **^{13}C{^{1}H} NMR** (100.06 MHz, 298.2 K, C$_6$D$_6$): δ (ppm) 154.5 (d, *1*-GeC$_6$H$_4$, $^2J_{PC}$ = 22.6 Hz), 148.1 (s, *p*-*C*$_6$H$_2$), 147.9 (s, *o*-GeC$_6$H$_3$), 147.0(s, *o*-*C*$_6$H$_2$), 143.6 (d, *i*-GeC$_6$H$_3$, $^3J_{PC}$ = 24.3 Hz), 138.6 (s, *i*-*C*$_6$H$_2$), 132.5 (d, P*C*$_6$H$_5$, $^2J_{PC}$ = 10.4 Hz), 132.4-132.0 (m, *5*-GeC$_6$H$_4$, *2*-GeC$_6$H$_4$, P*C*$_6$H$_5$), 130.9 (d, P*C*$_6$H$_5$, J_{PC} = 2.4 Hz), 129.6 (d, *3*-Ge*C*$_6$H$_4$, $^2J_{PC}$ = 6.6 Hz), 129.4 (s, *m*-Ge*C*$_6$H$_3$), 128.6-128.14 (m, *5*-GeC$_6$H$_4$, PC$_6$H$_5$), 128.0 (s, *p*-Ge*C*$_6$H$_3$, overlapping solvent signal), 127.0 (s, P*C*$_6$H$_5$, overlapping solvent signal), 125.0 (d, *4*-GeC$_6$H$_4$, $^3J_{PC}$ = 8.9 Hz), 120.5 (s, *m*-*C*$_6$H$_2$), 34.5 (s, *p*-*C*H(CH$_3$)$_2$), 30.9 (s, *o*-*C*H(CH$_3$)$_2$), 26.2 (s, *o*-CH(*C*H$_3$)$_2$), 24.0 (s, *o*-CH(*C*H$_3$)$_2$), 23.2 (s, *p*-CH(*C*H$_3$)$_2$). **^{31}P{^{1}H} NMR** (161.96 MHz, 298.2 K, C$_6$D$_6$) δ (ppm) 5.21 (br, 485 Hz hw, s). **^{11}B{^{1}H} NMR** (96.29 MHz, 298.2 K, C$_6$D$_6$) δ (ppm) 17.3 (d, $^1J_{PB}$ = 132 Hz). **Anal.** Calcd. (%) for C$_{54}$H$_{63}$BClGeP: C 75.25, H 7.37; found: C 75.89, H 7.74.

Synthesis of (*o*-PPh$_2$)C$_6$H$_4$(Ar*)GeBBr (5). Magnesium (42.0 mg, 1.73 mmol, 10.0 eq) and two drops of dibromoethane were stirred in tetrahydrofuran (2 mL). After few minutes anthracene (3.08 mg, 17.3 µmol, 0.10 eq) was added and the reaction mixture was stirred for five minutes followed by addition of (*o*-PPh$_2$)C$_6$H$_4$(Ar*)Ge(Br)BBr$_2$ **3** (184 mg, 173 µmol, 1.00 eq) dissolved in tetrahydrofuran (2 mL). The color of the solution turned from colorless to deep red. After four hours the solvent was removed *in vacuo*. After extraction with diethylether (2 mL) and pentane (2 mL) the product **5** could be obtained as a red powder (91.4 mg, 101 µmol, 57%). Red single crystals of **5** suitable for X-ray crystallography were obtained from a concentrated *n*-pentane solution at −40 °C. Analytical data: **^{1}H NMR** (400.13 MHz, 298.2 K, C$_6$D$_6$): δ (ppm) 7.50-7.45 (m, 4 H, C$_6$*H*$_5$), 7.43 (d, 2 H, *m*-GeC$_6$*H*$_3$, $^3J_{HH}$ = 7.3 Hz), 7.31 (dd, 1 H, *p*-GeC$_6$*H*$_3$, $^3J_{HH}$ = 7.0 Hz, $^3J_{HH}$ = 8.0 Hz), 7.28-7.25 (m, 1 H, *6*-GeC$_6$*H*$_4$), 7.14 (s, 4 H, *m*-C$_6$*H*$_2$, overlapping solvent signal), 7.02-6.98 (m, 2 H, C$_6$*H*$_5$), 6.98-6.93 (m, 5 H, *5*-GeC$_6$*H*$_4$, C$_6$*H*$_5$), 6.79-6.73 (m, 1 H, *3*-GeC$_6$*H*$_4$), 6.59-6.54 (m, 1 H, *4*-GeC$_6$*H*$_4$), 3.23 (sept., 4 H, *o*-C*H*(CH$_3$)$_2$, $^3J_{HH}$ = 7.0 Hz), 2.81 (sept., 2 H, *p*-C*H*(CH$_3$)$_2$, $^3J_{HH}$ = 6.9 Hz), 1.35 (d, 12 H, *o*-CH(C*H*$_3$)$_2$, $^3J_{HH}$ = 6.9 Hz), 1.21 (d, 12 H, *p*-CH(C*H*$_3$)$_2$, $^3J_{HH}$ = 6.8 Hz), 1.20 (d, 12 H, *o*-CH(C*H*$_3$)$_2$, $^3J_{HH}$ = 6.8 Hz). **^{13}C{^{1}H} NMR** (100.06 MHz, 298.2 K, C$_6$D$_6$): δ (ppm) 154.5 (s, *1*-Ge*C*$_6$H$_4$), 148.2 (s, *p*-*C*$_6$H$_2$), 147.7 (s, *o*-Ge*C*$_6$H$_3$), 147.0(s, *o*-*C*$_6$H$_2$), 143.7 (s, *i*-Ge*C*$_6$H$_3$), 138.4 (s, *i*-*C*$_6$H$_2$), 132.6 (d, P*C*$_6$H$_5$, J_{PC} = 10.3 Hz), 132.5-131.8 (m, *2*-Ge*C*$_6$H$_4$, *5*-Ge*C*$_6$H$_4$, *4*-Ge*C*$_6$H$_4$), 131.0 (s, P*C*$_6$H$_5$), 129.6 (d, *3*-Ge*C*$_6$H$_4$, $^2J_{PC}$ = 6.6 Hz), 129.4 (s, *m*-Ge*C*$_6$H$_3$), 128.5 (d, P*C*$_6$H$_5$, J_{PC} = 11.1 Hz), 128.3-127.2 (m, P*C*$_6$H$_5$, *p*-Ge*C*$_6$H$_3$, overlapping solvent signal), 125.0 (d, *4*-Ge*C*$_6$H$_4$, $^3J_{PC}$ = 8.5 Hz), 120.5 (s, *m*-*C*$_6$H$_2$), 34.5 (s, *p*-*C*H(CH$_3$)$_2$), 30.9 (s, *o*-*C*H(CH$_3$)$_2$), 26.2 (s, *o*-CH(*C*H$_3$)$_2$), 24.0 (s, *o*-CH(*C*H$_3$)$_2$), *p*-CH(*C*H$_3$)$_2$), 23.3 (s, *o*-CH(*C*H$_3$)$_2$). **^{31}P{^{1}H} NMR** (202.45 MHz, 298.2 K, C$_6$D$_6$) δ(ppm) 7.3 (m, 452 Hz hw). **^{11}B{^{1}H} NMR** (160.46 MHz, 298.2 K, C$_6$D$_6$) δ (ppm) 10.3 (d, $^1J_{PB}$ = 134.0 Hz). **Anal.** Calcd. (%) for C$_{60}$H$_{77}$BBrGeP: C 72.60, H 7.82; found: C 72.85, H 7.40.

Synthesis of (*o*-PPh$_2$)C$_6$H$_4${(Trip)C$_6$H$_3$(C$_6$H$_2$^{i}Pr$_3$GeBCl)} 6. (*o*-PPh$_2$)C$_6$H$_4$(Ar*)GeBCl **4** (59.1 mg, 68.4 µmol, 1.00 eq) was dissolved in *n*-hexane (6 mL) and exposed to a green LED (530 nm). After 16 hours the color of the reaction mixture changed from deep red to light yellow and a colorless participant was formed. NMR shows a quantitative reaction. All steps were carried out under exclusion of daylight. The solvent was reduced *in vacuo* and the product **6** filtered off as a colorless powder (43.5 mg, 50.2 µmol, 74%). Colorless single crystals of **6** suitable for X-ray crystallography were obtained by diffusing *n*-hexane into a concentrated benzene solution at room temperature. Analytical data: **^{1}H NMR** (700.29 MHz, 298.2 K, C$_6$D$_6$): δ (ppm) 7.84 (d, 2 H, *m*-GeC$_6$*H*$_3$, $^3J_{HH}$ = 7.5 Hz), 7.80-7.75 (m, 2 H, C$_6$*H*$_5$), 7.53-7.49 (m, 2 H, C$_6$*H*$_5$), 7.42 (t, 1 H, *p*-GeC$_6$*H*$_3$, $^3J_{HH}$ = 7.5 Hz), 7.36 (dd, 1 H, *m*-GeC$_6$*H*$_3$, $^3J_{HH}$ = 7.4 Hz, $^4J_{HH}$ = 1.0 Hz), 7.26 (d, 1 H, *m*-C$_6$*H*$_2$(Trip), $^4J_{HH}$ = 1.7 Hz), 7.11-6.94 (m, 9 H, *3*-C$_6$*H*$_4$, *6*-C$_6$*H*$_4$, *5*-C$_6$*H*$_4$, C$_6$*H*$_5$), 6.85 (d, 1 H, *m*-C$_6$*H*$_2$(Trip), $^4J_{HH}$ = 1.7 Hz), 6.75-6.70 (m, 1 H, *4*-GeC$_6$*H*$_4$), 6.17 (s, 1 H, *4*-GeC$_6$*H*$_2$), 3.78 (sept., 1 H, *3*-C*H*(CH$_3$)$_2$(GeC$_6$H$_2$), $^3J_{HH}$ = 6.8 Hz), 3.41 (sept., 1 H, *o*-C*H*(CH$_3$)$_2$(Trip), $^3J_{HH}$ = 6.9 Hz), 3.10 (sept., 1 H, *o*-C*H*(CH$_3$)$_2$(Trip), $^3J_{HH}$ = 6.8 Hz), 2.74 (sept., 1 H, *p*-C*H*(CH$_3$)$_2$(Trip), $^3J_{HH}$ = 6.9 Hz),

2.69 (d, 1 H, *6*-GeC$_6$*H*$_2$, $^3J_{PH}$ = 21.3 Hz), 2.02 (sept., 1 H, *1*-C*H*(CH$_3$)$_2$(GeC$_6$H$_2$), $^3J_{HH}$ = 6.7 Hz), 1.72 (sept., 1 H, *5*-C*H*(CH$_3$)$_2$(GeC$_6$H$_2$), $^3J_{HH}$ = 6.8 Hz), 1.46 (d, 3 H, *3*-CH(C*H*$_3$)$_2$(GeC$_6$H$_2$), $^3J_{HH}$ = 6.9 Hz), 1.38 (d, 3 H, *o*-CH(C*H*$_3$)$_2$(Trip), $^3J_{HH}$ = 6.9 Hz), 1.35 (d, 3 H, *o*-CH(C*H*$_3$)$_2$(Trip), $^3J_{HH}$ = 6.7 Hz), 1.21 (d, 3 H, *o*-CH(C*H*$_3$)$_2$(Trip), $^3J_{HH}$ = 6.5 Hz), 1.19 (d, 3 H, *5*-CH(C*H*$_3$)$_2$(GeC$_6$H$_2$), $^3J_{HH}$ = 6.7 Hz), 1.18 (d, 3 H, *p*-CH(C*H*$_3$)$_2$(Trip), $^3J_{HH}$ = 7.1 Hz), 1.15 (d, 3 H, *p*-CH(C*H*$_3$)$_2$(Trip), $^3J_{HH}$ = 6.9 Hz), 0.98 (d, 3 H, *o*-CH(C*H*$_3$)$_2$(Trip), $^3J_{HH}$ = 6.9 Hz), 0.89 (d, 3 H, *1*-CH(C*H*$_3$)$_2$(GeC$_6$H$_2$), $^3J_{HH}$ = 7.5 Hz), 0.88 (d, 3 H, *1*-CH(C*H*$_3$)$_2$(GeC$_6$H$_2$), $^3J_{HH}$ = 7.1 Hz), 0.84 (d, 3 H, *o*-CH(C*H*$_3$)$_2$(Trip), $^3J_{HH}$ = 6.7 Hz), 0.72 (d, 3 H, *5*-CH(C*H*$_3$)$_2$(GeC$_6$H$_2$), $^3J_{HH}$ = 7.1 Hz). **^{13}C{^{1}H} NMR** (176.09 MHz, 298.2 K, C$_6$D$_6$): δ (ppm) 151.8 (d, *1*-GeC$_6$H$_4$, $^2J_{PC}$ = 23.7 Hz), 151.0 (s, *o*-Ge*C*$_6$H$_3$), 149.3 (d, *5*-GeC$_6$H$_2$, $^3J_{PC}$ = 3.6 Hz), 146.9 (s, *p*-*C*$_6$H$_2$(Trip)), 146.2 (s, *o*-*C*$_6$H$_2$(Trip)), 145.8 (s, *o*-*C*$_6$H$_2$(Trip)), 144.2 (d, *i*-GeC$_6$H$_3$, $^3J_{PC}$ = 8.1 Hz), 142.9 (s, *o*-GeC$_6$H$_3$), 141.9 (s, *3*-GeC$_6$H$_2$), 139.3 (s, *i*-C$_6$H$_2$(Trip)), 134.8 (d, *6*-Ge*C*$_6$H$_4$, $^3J_{PC}$ = 11.7 Hz), 133.3 (d, P*C*$_6$H$_5$, J_{PC} = 8.1 Hz), 132.6 (d, *2*-Ge*C*$_6$H$_4$, $^1J_{PC}$ = 74.5 Hz), 132.5 (d, *3*-Ge*C*$_6$H$_4$, $^2J_{PC}$ = 5.2 Hz), 132.4 (d, P*C*$_6$H$_5$, J_{PC} = 9.1 Hz), 131.0 (s, *2*-GeC$_6$H$_2$), 130.4 (s, PC$_6$H$_5$), 130.2 (s, PC$_6$H$_5$), 129.8 (s, *5*-GeC$_6$H$_4$), 127.9 (d, PC$_6$H$_5$, J_{PC} = 10.1 Hz), 127.4 (s, PC$_6$H$_5$), 127.2-126.3 (m, *m*-GeC$_6$H$_3$, *4*-GeC$_6$H$_4$, PC$_6$H$_5$, overlapping solvent signal), 125.9 (s, *p*-Ge*C*$_6$H$_3$), 124.4 (s, *m*-GeC$_6$H$_3$), 119.9 (s, *m*-*C*$_6$H$_2$(Trip)), 119.6 (s, *m*-*C*$_6$H$_2$(Trip)), 115.0 (s, *4*-GeC$_6$H$_2$), 58.8 (d, *1*-GeC$_6$H$_2$, $^3J_{PC}$ = 15.9 Hz), 34.7 (br s, *1*-GeC$_6$H$_2$(*C*H(CH$_3$)$_2$)), 35.2 (d, *1*-GeC$_6$H$_2$(*C*H(CH$_3$)$_2$), $^4J_{PC}$ = 3.3 Hz), 33.7 (s, *p*-*C*H(CH$_3$)$_2$(Trip)), 32.9 (s, *5*-GeC$_6$H$_2$(*C*H(CH$_3$)$_2$)), 29.5 (s, *o*-*C*H(CH$_3$)$_2$(Trip)), 29.3 (s, *o*-*C*H(CH$_3$)$_2$(Trip)), 28.3 (s, *3*-GeC$_6$H$_2$(*C*H(CH$_3$)$_2$)), 25.4 (s, *o*-CH(*C*H$_3$)$_2$(Trip)), 24.7 (s, *o*-CH(*C*H$_3$)$_2$(Trip)), 23.4 (s, *p*-CH(*C*H$_3$)$_2$(Trip)), 23.3 (s, *p*-CH(*C*H$_3$)$_2$(Trip)), 22.7 (s, *o*-CH(*C*H$_3$)$_2$(Trip)), 22.5 (s, *3*-GeC$_6$H$_2$(CH(*C*H$_3$)$_2$)), 22.3 (s, *o*-CH(*C*H$_3$)$_2$(Trip)), 21.4 (s, *5*-GeC$_6$H$_2$(CH(*C*H$_3$)$_2$)), 20.8 (s, *3*-GeC$_6$H$_2$(CH(*C*H$_3$)$_2$)), 20.3 (s, *1*-GeC$_6$H$_2$(CH(*C*H$_3$)$_2$)), 20.1 (s, *1*-GeC$_6$H$_2$(CH(*C*H$_3$)$_2$)), 19.9 (s, *5*-GeC$_6$H$_2$(CH(*C*H$_3$)$_2$)). **^{31}P{^{1}H} NMR** (121.49 MHz, 298.2 K, C$_6$D$_6$) δ (ppm) 13.4 (s). **^{11}B{^{1}H} NMR** (96.29 MHz, 298.2 K, C$_6$D$_6$) δ (ppm) −4.5 (s). **Anal.** Calcd. (%) for C$_{54}$H$_{63}$BClGeP: C 75.25, H 7.37; found: C 74.82, H 8.31.

Synthesis of (*o*-PPh$_2$)C$_6$H$_4${(Trip)C$_6$H$_3$(C$_6$H$_2$^{i}Pr$_3$GeBBr)} 7. (*o*-PPh$_2$)C$_6$H$_4$(Ar*)GeBBr **5** (49.6 mg, 54.6 µmol, 1.00 eq) was dissolved in hexane (6 mL) and exposed to a green LED (530 nm). After 16 hours the color of the reaction mixture had changed from deep red to light yellow. NMR shows a quantitative reaction. All steps were carried out under exclusion of daylight. After washing with cold *n*-pentane (0.2 mL) the product **7** could be obtained as a colorless powder (37.5 mg, 41.3 µmol, 76%). Light yellow single crystals of **7** suitable for X-ray crystallography were obtained from a concentrated *n*-hexane solution at room temperature. Analytical data: **^{1}H NMR** (400.13 MHz, 298.2 K, C$_6$D$_6$): δ (ppm) 7.82 (d, 2 H, *m*-GeC$_6$*H*$_3$, $^3J_{HH}$ = 7.5 Hz), 7.77-7.70 (m, 2 H, C$_6$*H*$_5$), 7.60-7.52 (m, 2 H, C$_6$*H*$_5$), 7.42 (t, 1 H, *p*-GeC$_6$*H*$_3$, $^3J_{HH}$ = 7.5 Hz), 7.31 (dd, 1 H, *m*-GeC$_6$*H*$_3$, $^3J_{HH}$ = 7.5 Hz, $^4J_{HH}$ = 1.0 Hz), 7.25 (d, 1 H, *m*-C$_6$*H*$_2$(Trip), $^4J_{HH}$ = 1.8 Hz), 7.14-6.91 (m, 9 H, C$_6$*H*$_4$, C$_6$*H*$_5$), 6.87 (d, 1 H, *m*-C$_6$*H*$_2$(Trip), $^4J_{HH}$ = 1.6 Hz), 6.75-6.70 (m, 1 H, C$_6$*H*$_4$), 6.16 (s, 1 H, *4*-GeC$_6$*H*$_2$), 3.77 (sept., 1 H, *3*-C*H*(CH$_3$)$_2$(GeC$_6$H$_2$), $^3J_{HH}$ = 6.4 Hz), 3.43 (sept., 1 H, *o*-C*H*(CH$_3$)$_2$(Trip), $^3J_{HH}$ = 6.1 Hz), 3.10 (sept., 1 H, *o*-C*H*(CH$_3$)$_2$(Trip), $^3J_{HH}$ = 6.8 Hz), 2.73 (sept., 1 H, *p*-C*H*(CH$_3$)$_2$(Trip), $^3J_{HH}$ = 6.9 Hz), 2.63 (d, 1 H, *6*-GeC$_6$*H*$_2$, $^3J_{PH}$ = 22.2 Hz), 1.96 (sept., 1 H, *1*-C*H*(CH$_3$)$_2$(GeC$_6$H$_2$), $^3J_{HH}$ = 6.8 Hz), 1.65 (sept., 1 H, *5*-C*H*(CH$_3$)$_2$(GeC$_6$H$_2$), $^3J_{HH}$ = 6.0 Hz), 1.44 (d, 3 H, *3*-CH(C*H*$_3$)$_2$(GeC$_6$H$_2$), $^3J_{HH}$ = 7.0 Hz), 1.37 (d, 3 H, *o*-CH(C*H*$_3$)$_2$(Trip), $^3J_{HH}$ = 6.8 Hz), 1.35 (d, 3 H, *o*-CH(C*H*$_3$)$_2$(Trip), $^3J_{HH}$ = 6.8 Hz), 1.23 (d, 6 H, *3*-CH(C*H*$_3$)$_2$(GeC$_6$H$_2$), *5*-CH(C*H*$_3$)$_2$(GeC$_6$H$_2$), $^3J_{HH}$ = 6.7 Hz), 1.16 (d, 3 H, *p*-CH(C*H*$_3$)$_2$(Trip), $^3J_{HH}$ = 7.0 Hz), 1.14 (d, 3 H, *p*-CH(C*H*$_3$)$_2$(Trip), $^3J_{HH}$ = 7.0 Hz), 1.14 (d, 3 H, *o*-CH(C*H*$_3$)$_2$(Trip), $^3J_{HH}$ = 6.8 Hz), 0.88-0.80 (m, 9 H, *1*-CH(C*H*$_3$)$_2$(GeC$_6$H$_2$), *o*-CH(C*H*$_3$)$_2$(Trip)), 0.66 (d, 3 H, *5*-CH(C*H*$_3$)$_2$(GeC$_6$H$_2$), $^3J_{HH}$ = 6.8 Hz). **^{13}C{^{1}H} NMR** (100.06 MHz, 298.2 K, C$_6$D$_6$): δ (ppm) 152.5 (d, *1*-Ge*C*$_6$H$_4$, $^2J_{PC}$ = 10.3 Hz), 152.0 (s, *o*-Ge*C*$_6$H$_3$), 151.0 (d, *5*-Ge*C*$_6$H$_2$, $^3J_{PC}$ = 3.3 Hz), 147.8 (s, *p*-*C*$_6$H$_2$(Trip)), 147.0 (s, *o*-*C*$_6$H$_2$(Trip)), 146.6 (s, *o*-*C*$_6$H$_2$(Trip)), 144.4 (d, *i*-Ge*C*$_6$H$_3$, $^3J_{PC}$ = 10.3 Hz), 143.8 (s, *o*-Ge*C*$_6$H$_3$), 142.5 (s, *3*-Ge*C*$_6$H$_2$), 140.1 (s, *i*-*C*$_6$H$_2$(Trip)), 134.8 (d, Ge*C*$_6$H$_4$, J_{PC} = 11.8 Hz), 134.1 (d, P*C*$_6$H$_5$, J_{PC} = 8.0 Hz), 133.5-131.2 (m, Ge*C*$_6$H$_4$, P*C*$_6$H$_5$), 132.6 (d, Ge*C*$_6$H$_4$, J_{PC} = 11.8 Hz), 131.1 (br s, P*C*$_6$H$_5$), 130.5 (br s, P*C*$_6$H$_5$), 128.8 (d, P*C*$_6$H$_5$, J_{PC} = 10.1 Hz), 128.3-127.0 (m, *m*-Ge*C*$_6$H$_3$, Ge*C*$_6$H$_4$, P*C*$_6$H$_5$, overlapping solvent signal),

126.9 (s, *p*-Ge*C*$_6$H$_3$), 125.2 (s, *p*-Ge*C*$_6$H$_3$), 120.7 (s, *m*-*C*$_6$H$_2$(Trip)), 120.4 (s, *m*-*C*$_6$H$_2$(Trip)), 115.6 (s, *4*-Ge*C*$_6$H$_2$), 60.0 (d, *1*-Ge*C*$_6$H$_2$, $^3J_{PC}$ = 14.8 Hz), 35.2 (s, *1*-GeC$_6$H$_2$(*C*H(CH$_3$)$_2$)), 34.7-34.2 (m, *6*-GeC$_6$H$_2$(*C*H(CH$_3$)$_2$)), *p*-*C*H(CH$_3$)$_2$(Trip)), 33.4 (s, *5*-GeC$_6$H$_2$(*C*H(CH$_3$)$_2$)), 30.3 (s, *o*-*C*H(CH$_3$)$_2$(Trip)), 30.1 (s, *o*-*C*H(CH$_3$)$_2$(Trip)), 29.1 (s, *3*-GeC$_6$H$_2$(*C*H(CH$_3$)$_2$)), 26.3 (s, *o*-CH(*C*H$_3$)$_2$(Trip)), 25.5 (s, *o*-CH(*C*H$_3$)$_2$(Trip)), 24.2 (s, *p*-CH(*C*H$_3$)$_2$(Trip)), 24.1 (s, *p*-CH(*C*H$_3$)$_2$(Trip)), 23.5 (s, *3*-GeC$_6$H$_2$(CH(*C*H$_3$)$_2$)), 23.4 (s, *o*-CH(*C*H$_3$)$_2$(Trip)), 23.3 (s, *o*-CH(*C*H$_3$)$_2$(Trip)), 22.3 (s, *5*-GeC$_6$H$_2$(CH(*C*H$_3$)$_2$)), 21.5 (s, *3*-GeC$_6$H$_2$(CH(*C*H$_3$)$_2$)), 21.0 (s, *1*-GeC$_6$H$_2$(CH(*C*H$_3$)$_2$)), 20.8 (s, *1*-GeC$_6$H$_2$(CH(*C*H$_3$)$_2$)), 20.5 (s, *5*-GeC$_6$H$_2$(CH(*C*H$_3$)$_2$)). **^{31}P{^{1}H} NMR** (121.49 MHz, 298.2 K, C$_6$D$_6$) δ(ppm) 17.0 (s). **^{11}B{^{1}H} NMR** (96.29 MHz, 298.2 K, C$_6$D$_6$) δ(ppm) −10.1 (s). **Anal.** Calcd. (%) for C$_{54}$H$_{63}$BBrGeP: C 71.56, H 7.01; found: C 71.44, H 6.79.

NMR spectroscopy

NMR spectra of compound **2**.

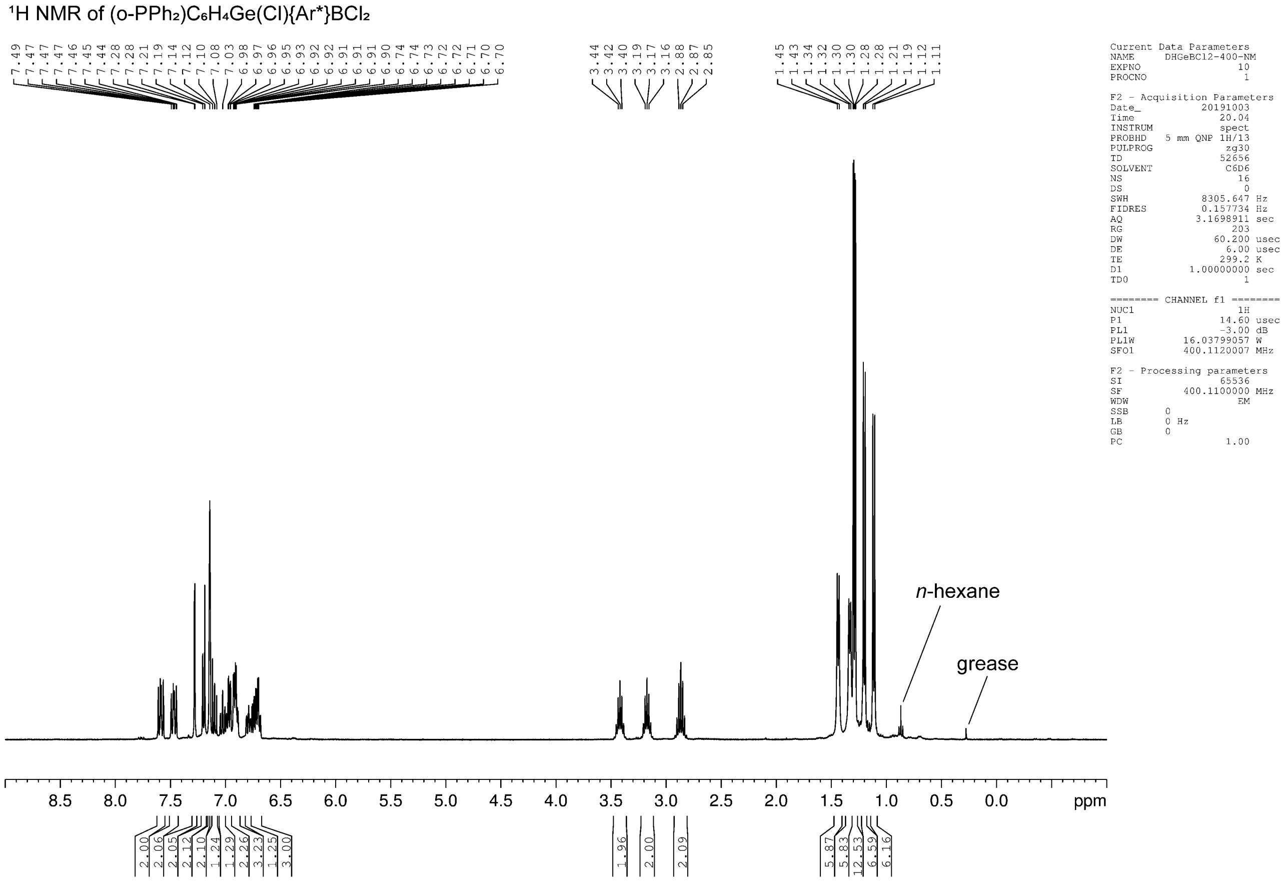

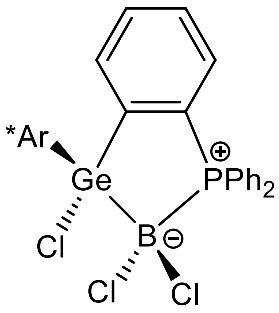

Figure S1. ^{1}H NMR of compound **2**.

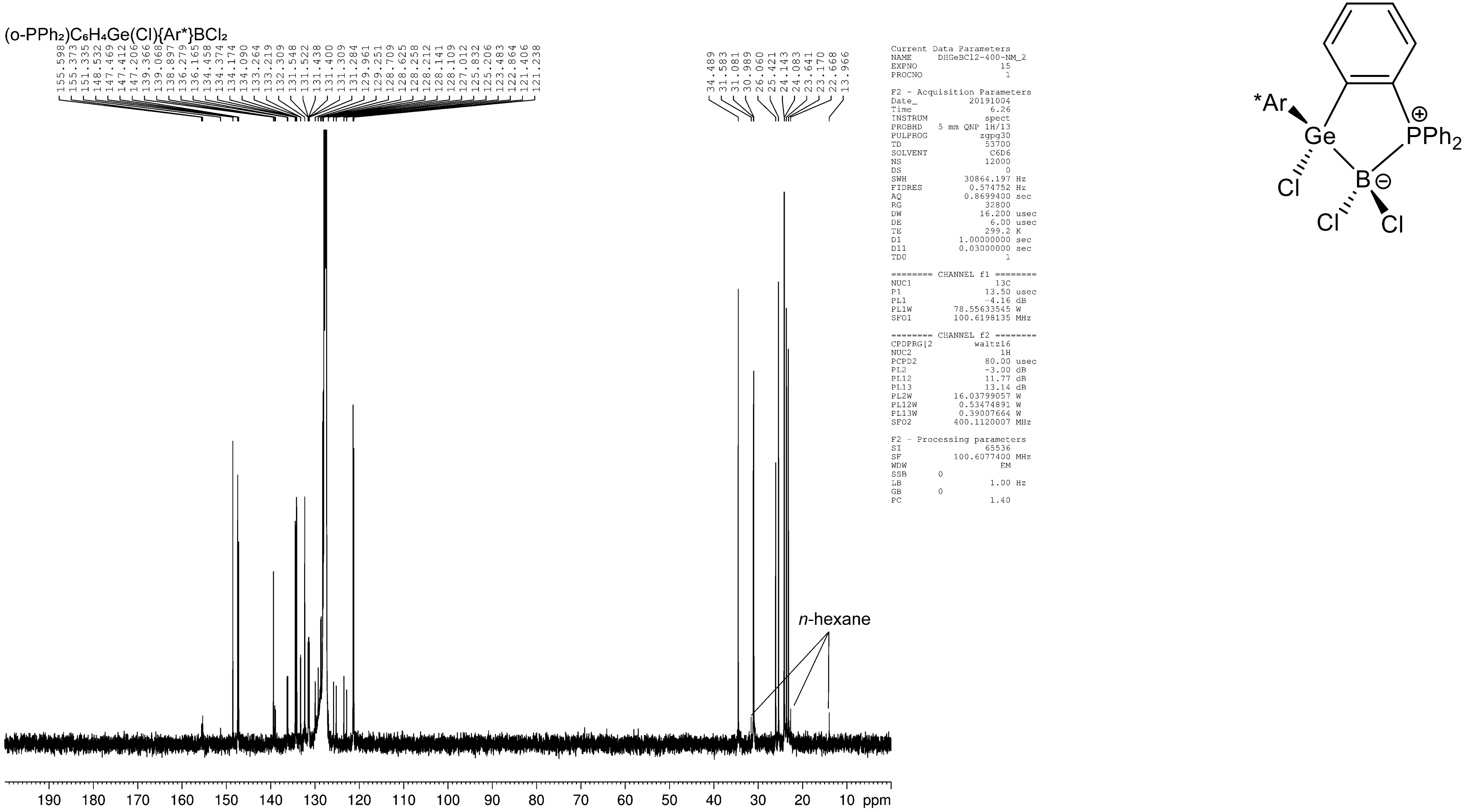

Figure S2. $^{13}C\{^{1}H\}$ NMR spectrum of compound **2**.

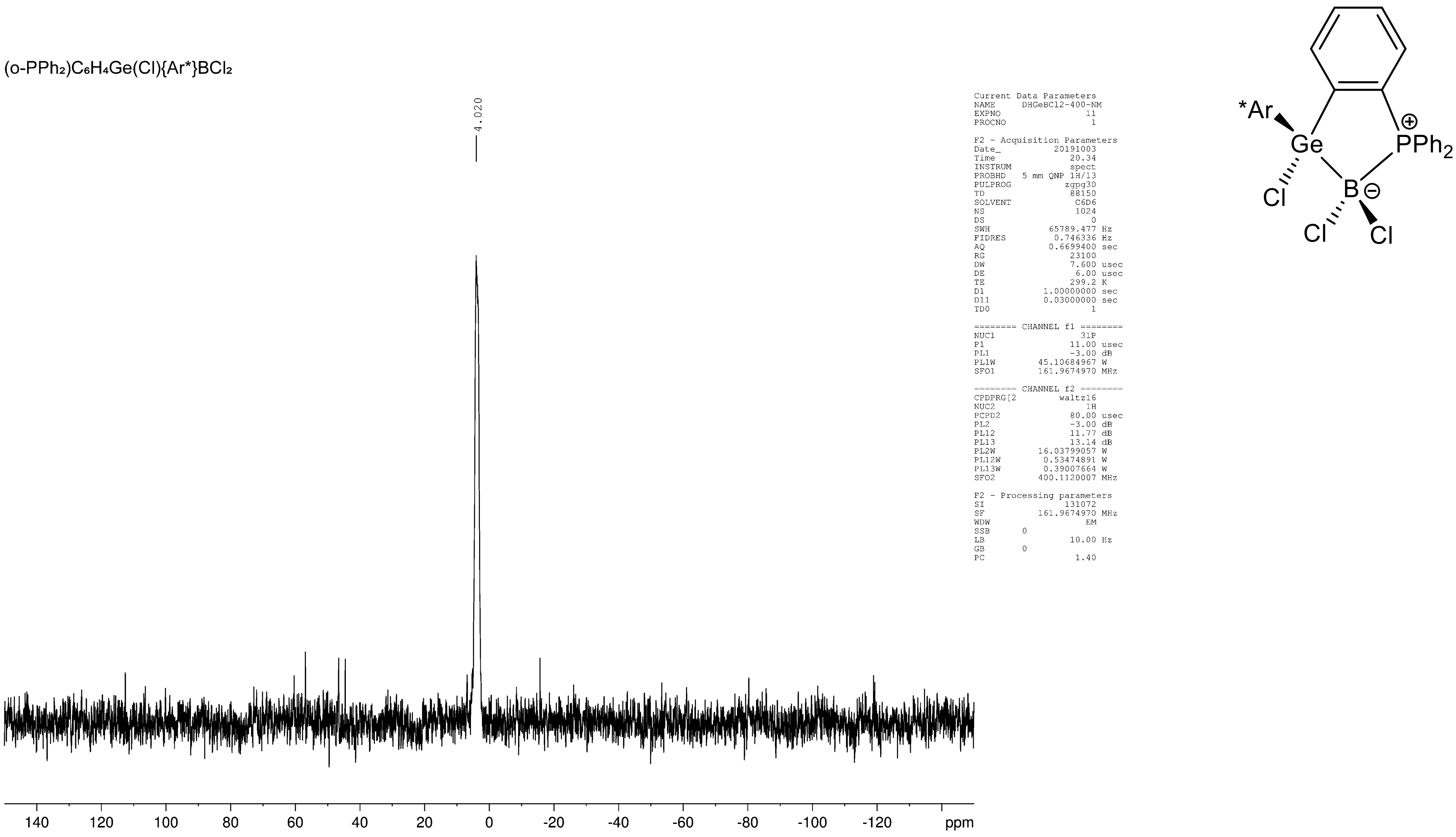

Figure S3. $^{31}P\{^{1}H\}$ NMR spectrum of compound **2**.

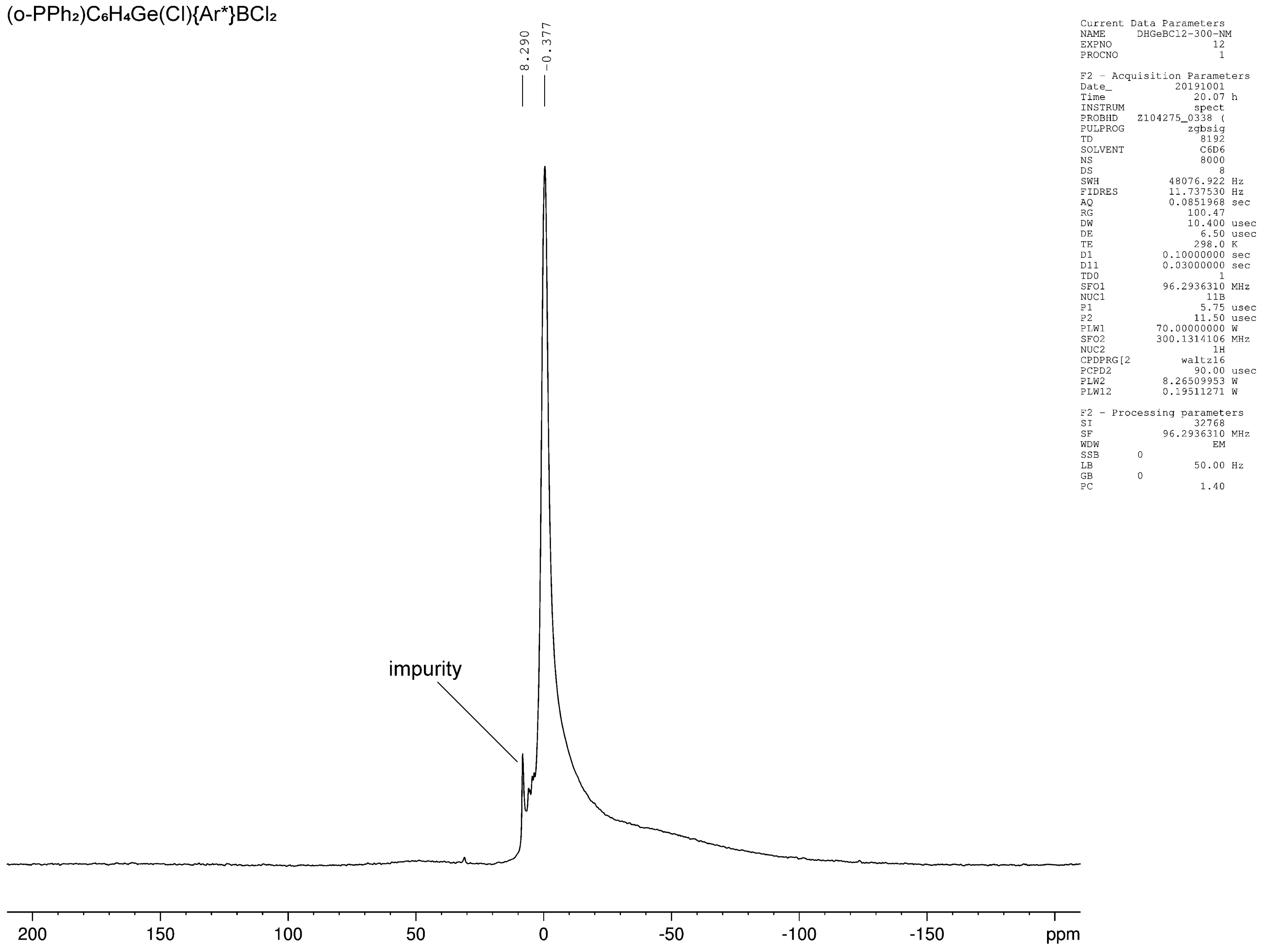

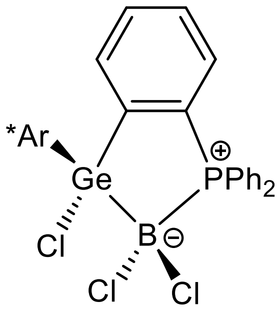

Figure S4. $^{11}B\{^1H\}$ NMR spectrum of compound **2**.

NMR spectra of compound **3**.

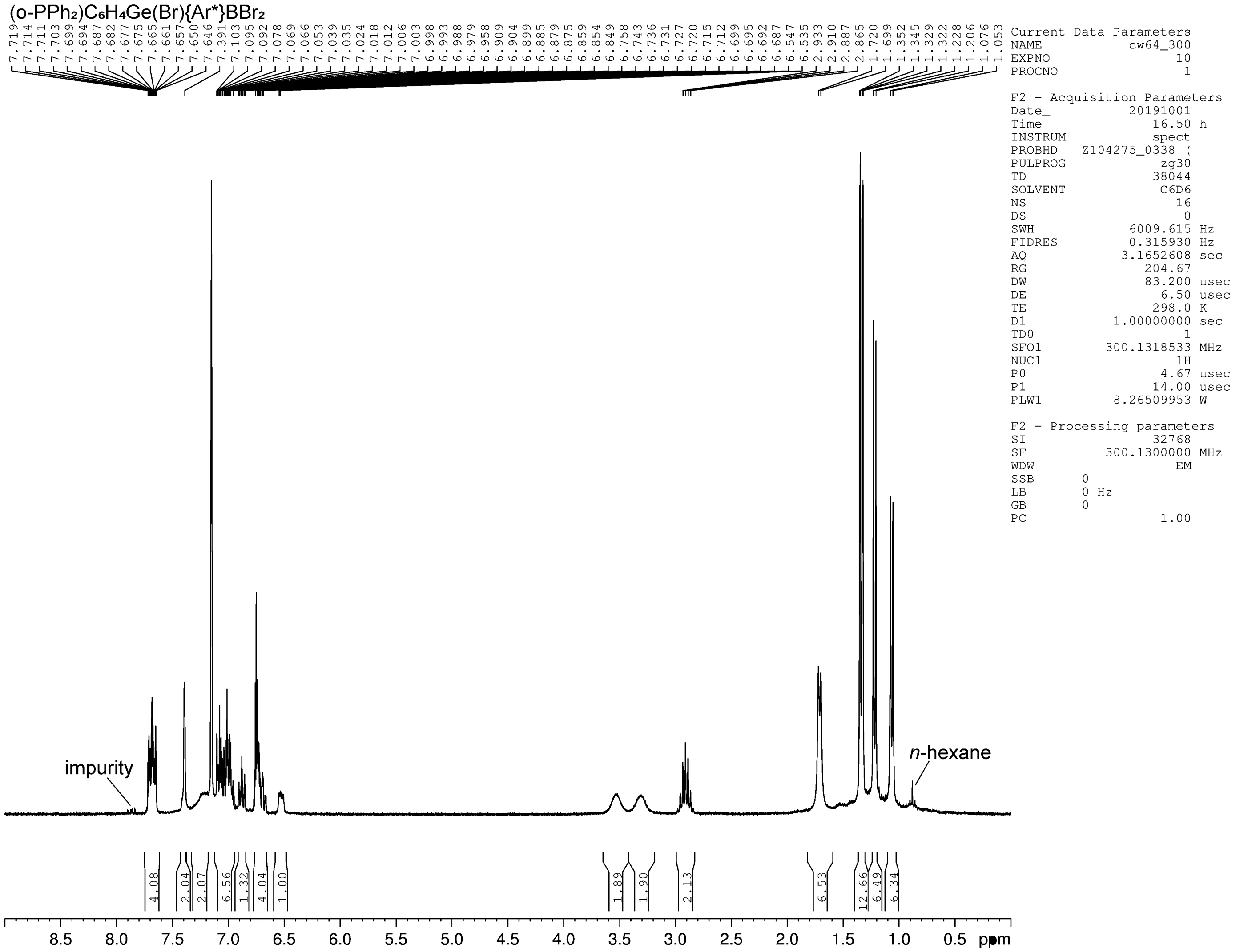

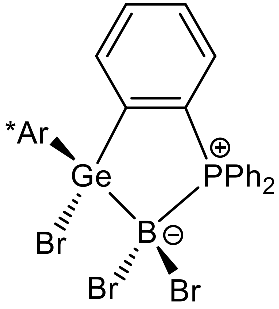

Figure S5. ^{1}H NMR of compound **3**.

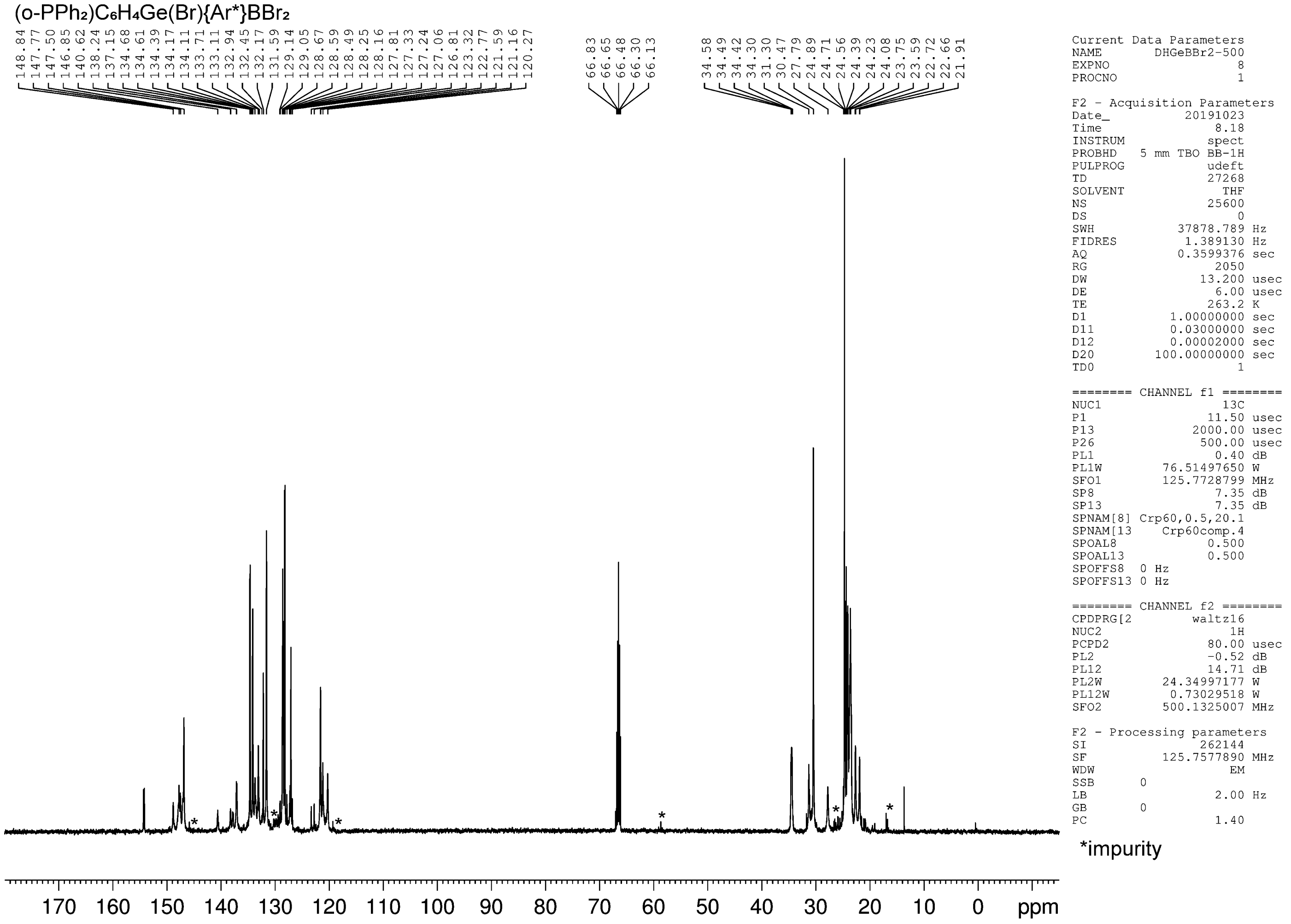

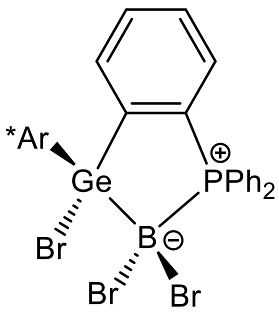

Figure S6. $^{13}C\{^{1}H\}$ NMR spectrum of compound **3**.

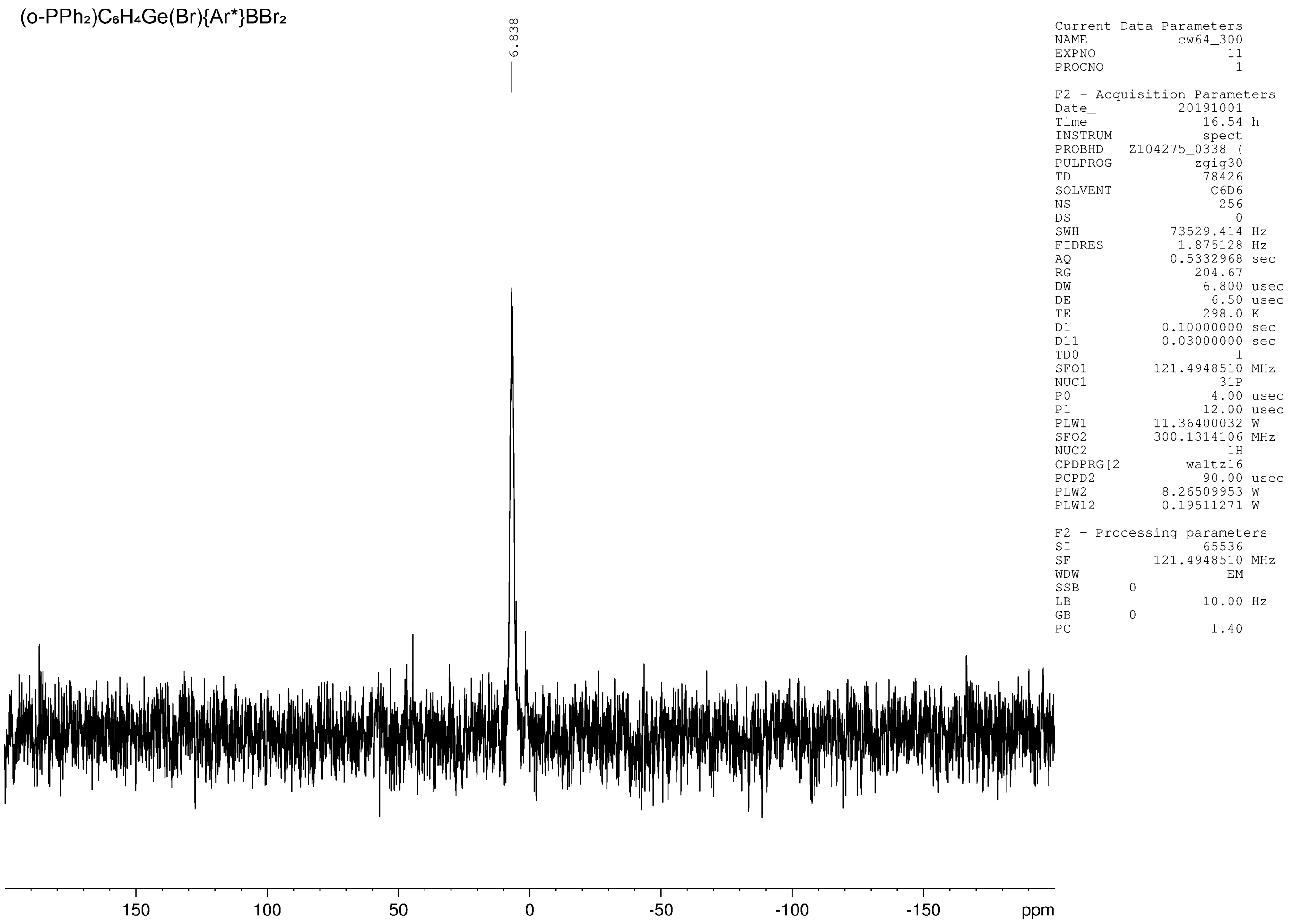

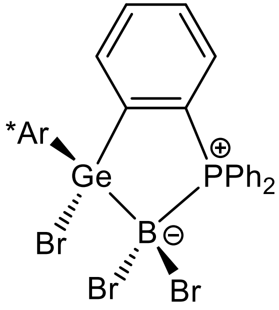

Figure S7. $^{31}P\{^{1}H\}$ NMR spectrum of compound **3**.

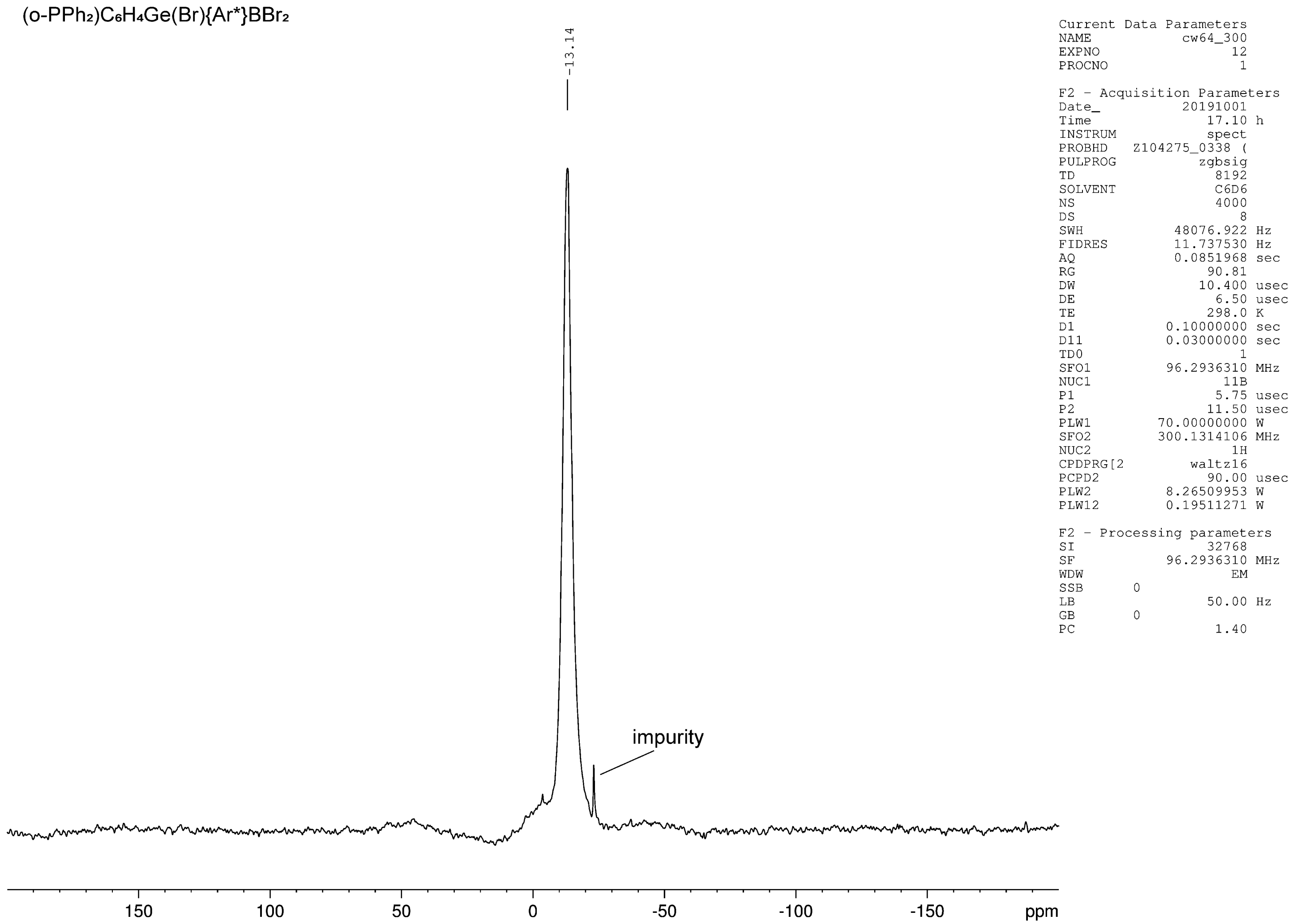

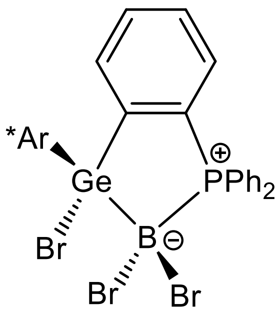

Figure S8. $^{11}B\{^{1}H\}$ NMR spectrum of compound **3**.

NMR spectra of compound **4**.

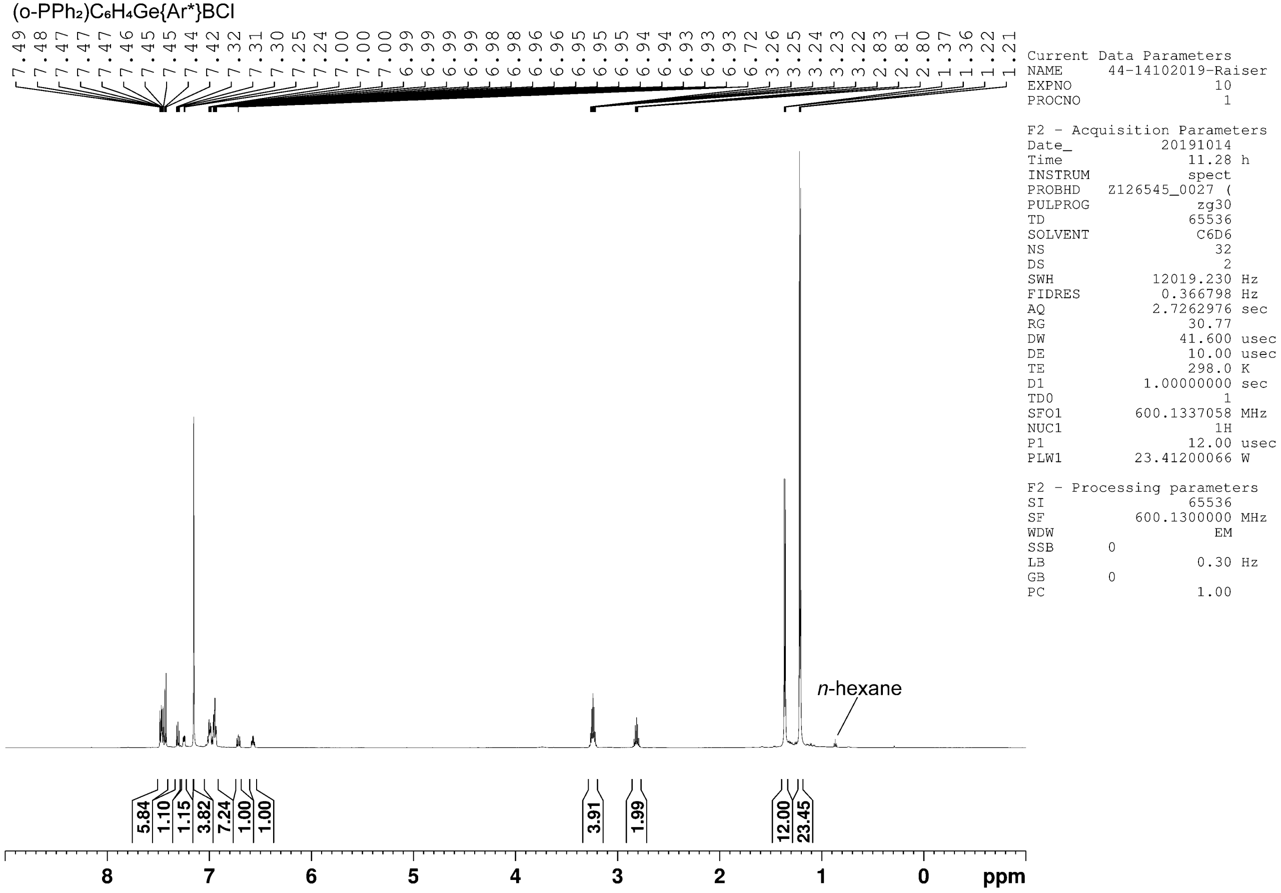

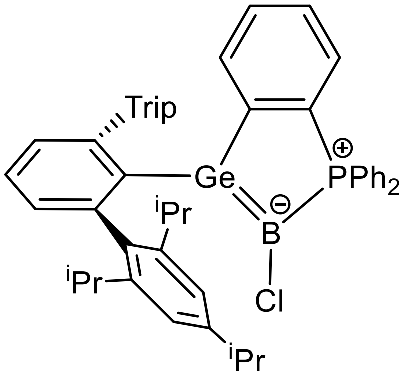

Figure S9. ^{1}H NMR spectrum of compound **4**.

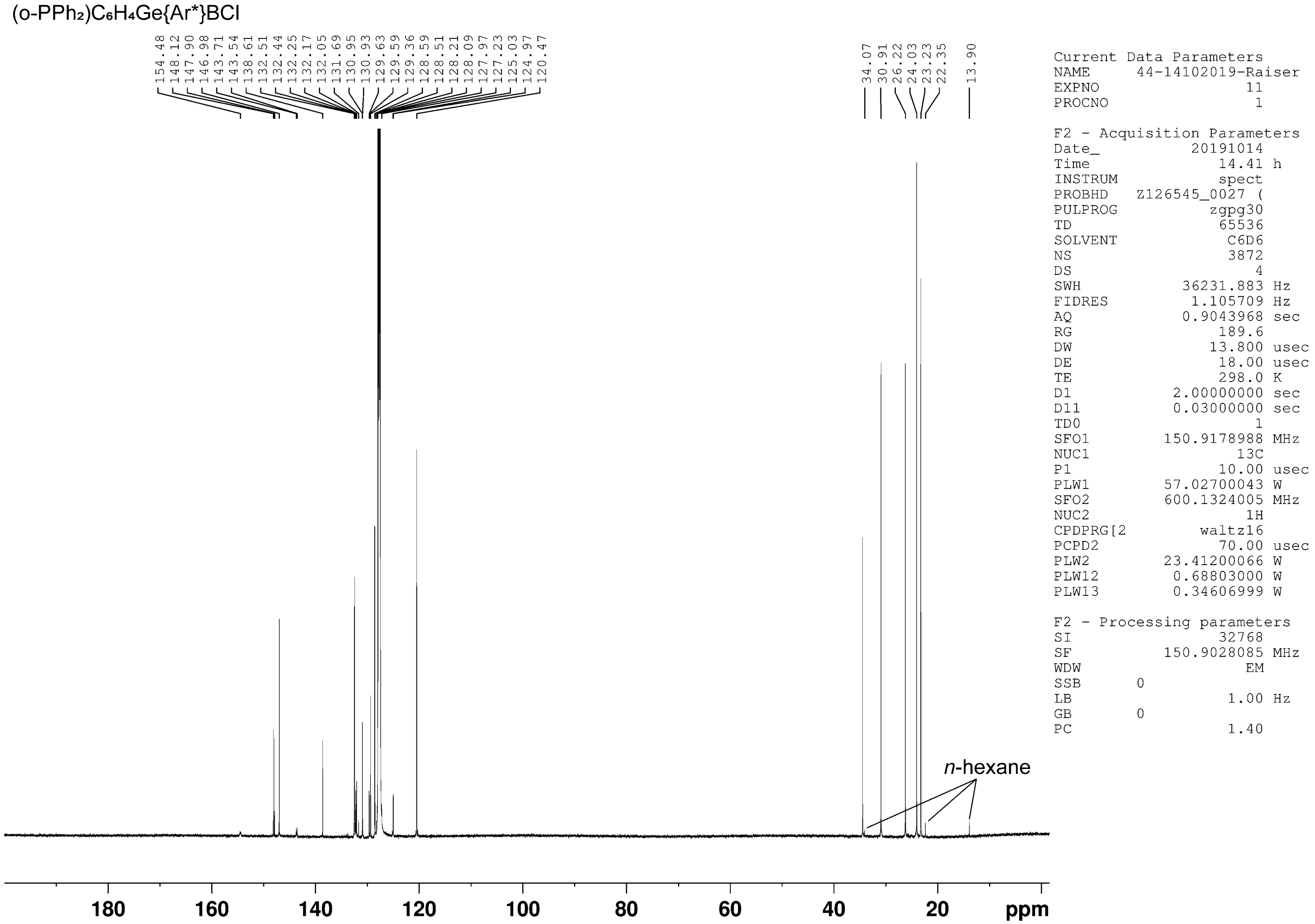

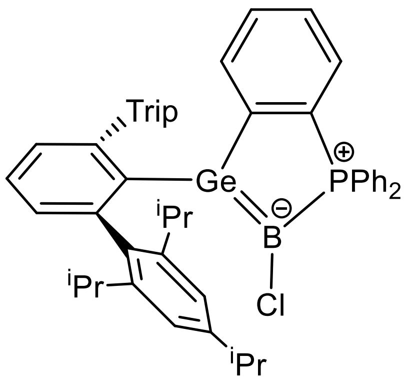

Figure S10. $^{13}C\{^{1}H\}$ NMR spectrum of compound **4**.

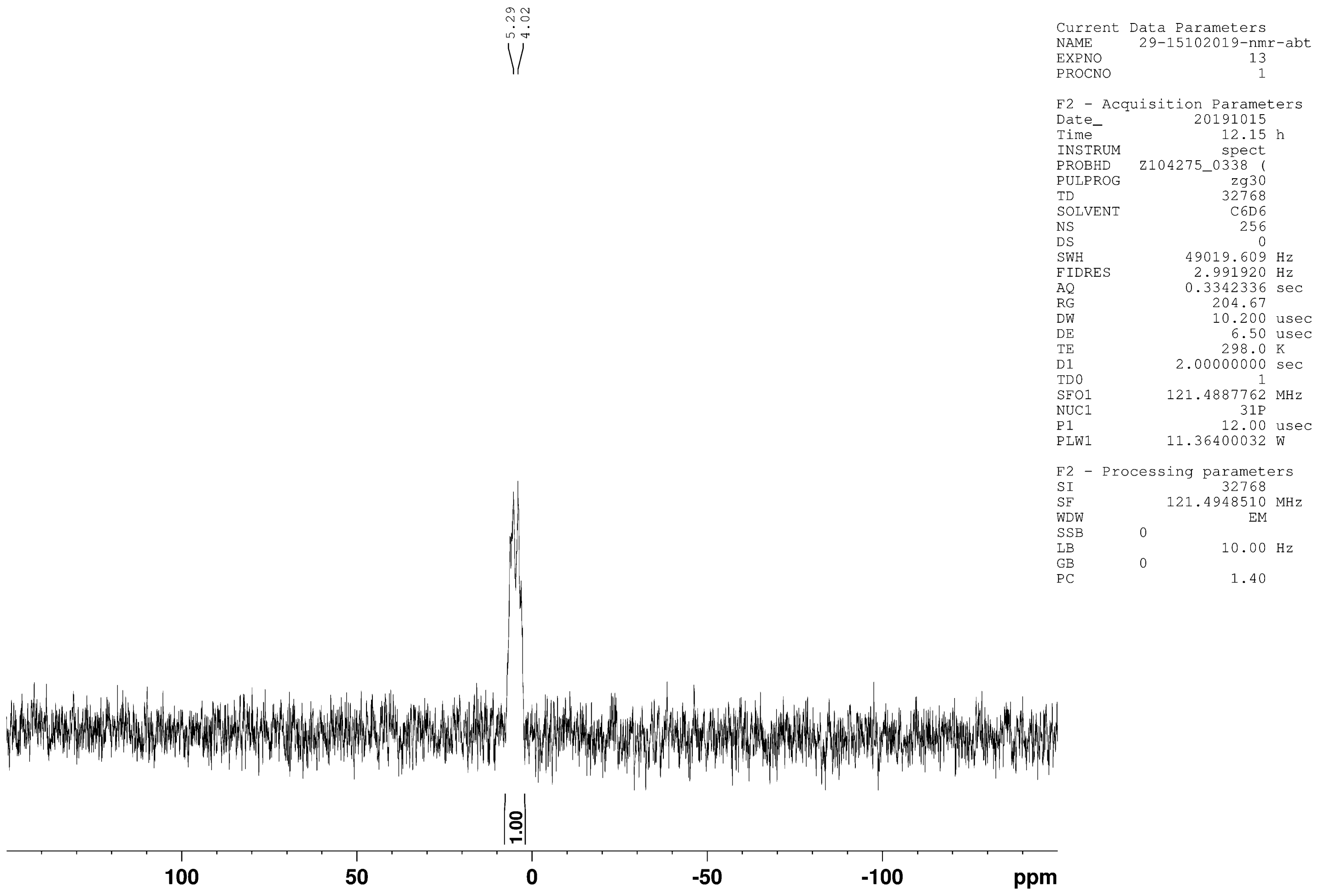

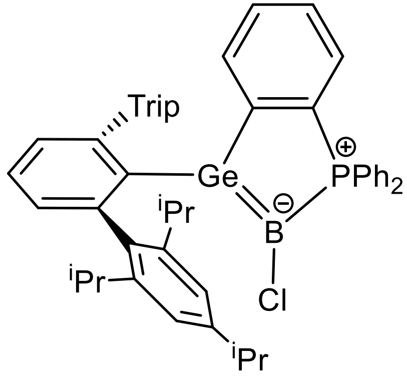

Figure S11. $^{31}P\{^{1}H\}$ NMR spectrum of compound **4**.

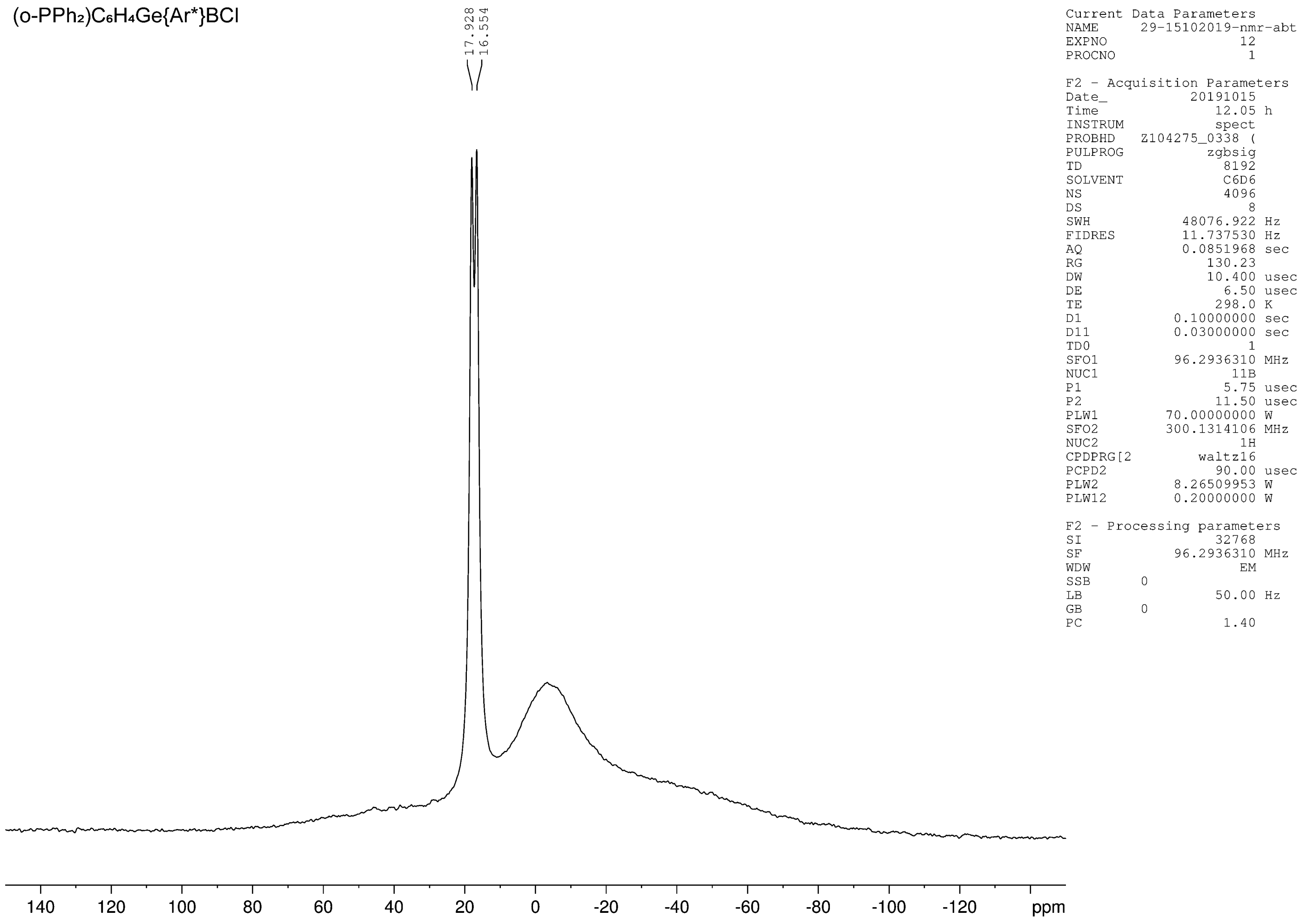


```
Current Data Parameters
NAME      29-15102019-nmr-abt
EXPNO                  12
PROCNO                  1

F2 - Acquisition Parameters
Date_            20191015
Time                12.05 h
INSTRUM             spect
PROBHD   Z104275_0338 (
PULPROG           zgbsig
TD                   8192
SOLVENT              C6D6
NS                   4096
DS                      8
SWH             48076.922 Hz
FIDRES          11.737530 Hz
AQ              0.0851968 sec
RG                 130.23
DW                 10.400 usec
DE                   6.50 usec
TE                  298.0 K
D1             0.10000000 sec
D11            0.03000000 sec
TD0                     1
SFO1           96.2936310 MHz
NUC1                  11B
P1                   5.75 usec
P2                  11.50 usec
PLW1          70.00000000 W
SFO2          300.1314106 MHz
NUC2                   1H
CPDPRG[2          waltz16
PCPD2               90.00 usec
PLW2           8.26509953 W
PLW12          0.20000000 W

F2 - Processing parameters
SI                  32768
SF             96.2936310 MHz
WDW                    EM
SSB      0
LB                  50.00 Hz
GB       0
PC                   1.40
```

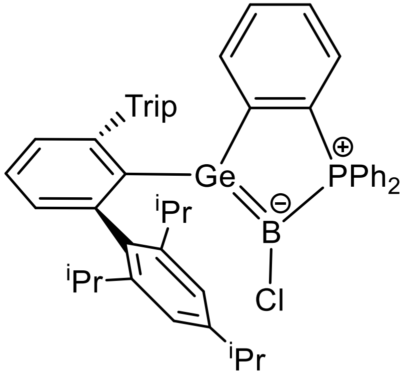

Figure S12. $^{11}B\{^{1}H\}$ NMR spectrum of compound **4**.

NMR spectra of compound **5**.

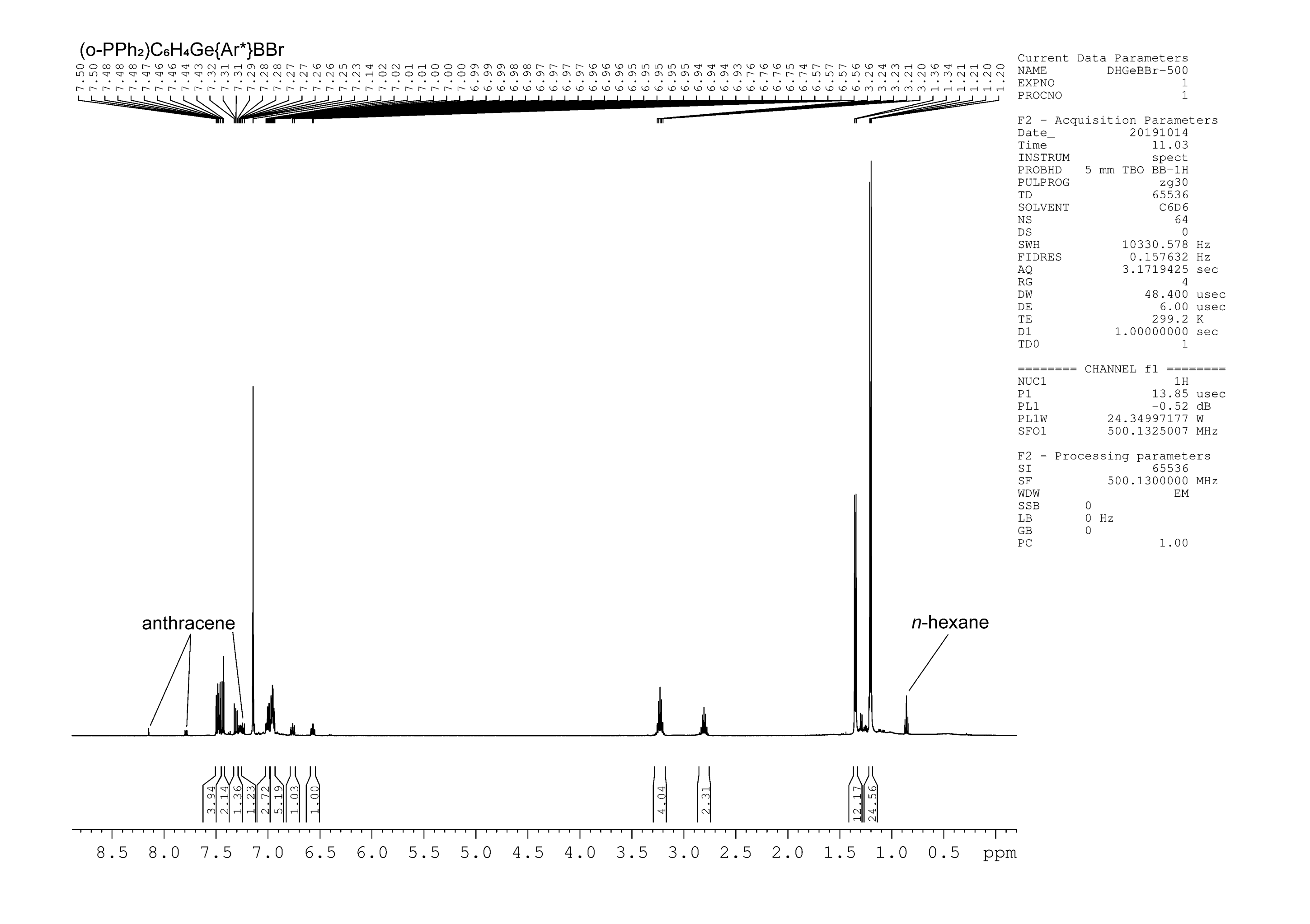

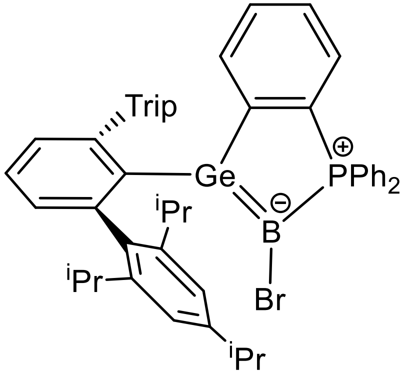

Figure S13. ^{1}H NMR spectrum of compound **5**.

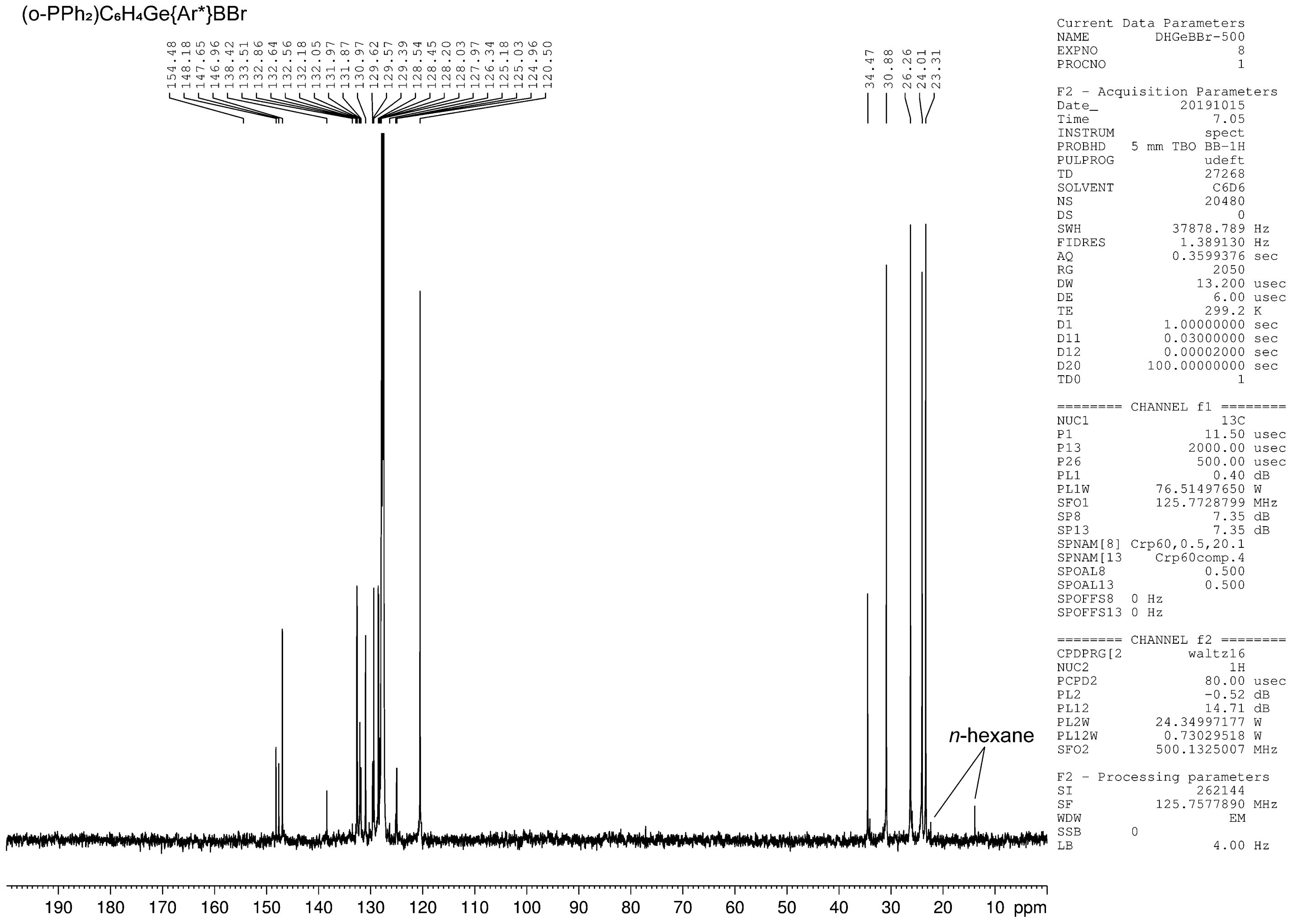

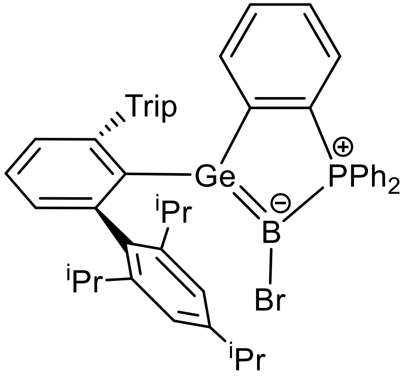

Figure S14. $^{13}C\{^{1}H\}$ NMR spectrum of compound **5**

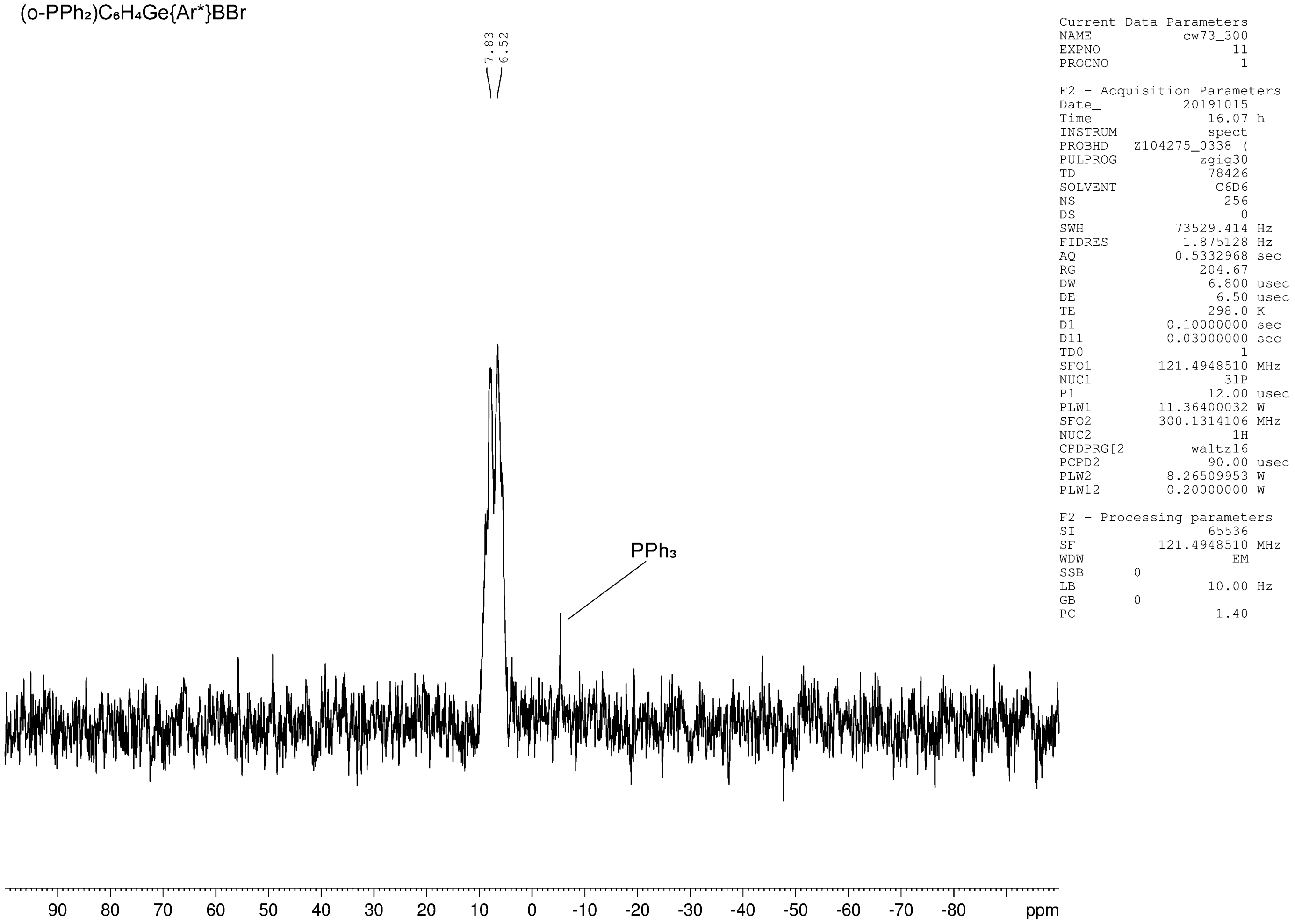

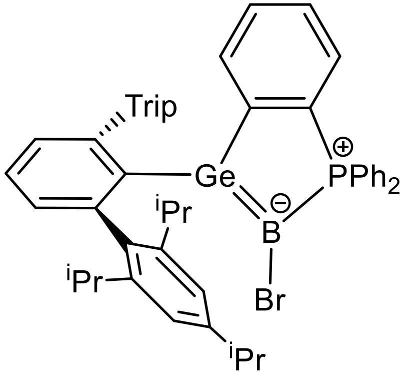

Figure S15. $^{31}P\{^{1}H\}$ NMR spectrum of compound **5**.

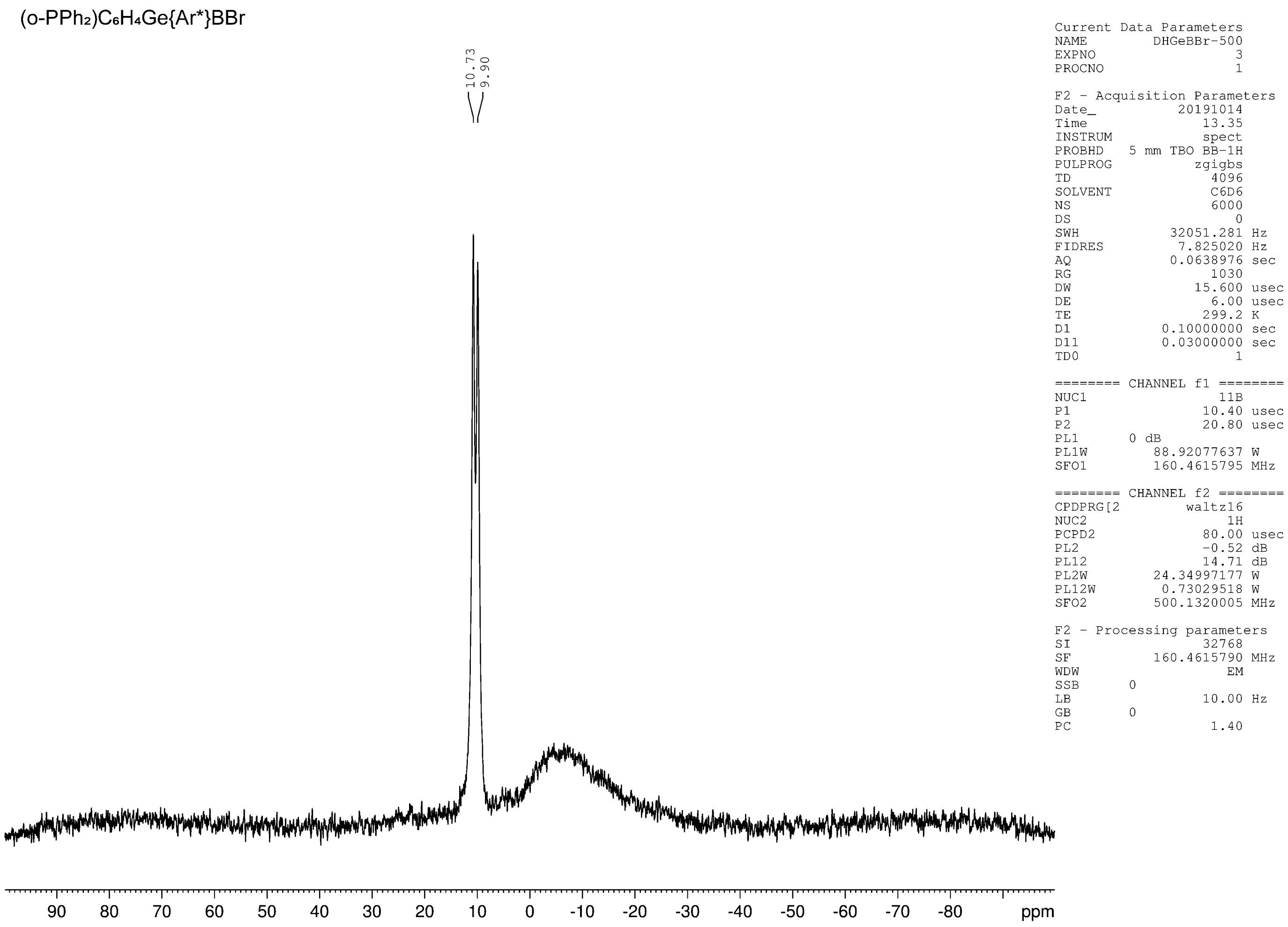


```
Current Data Parameters
NAME          DHGeBBr-500
EXPNO                   3
PROCNO                  1

F2 - Acquisition Parameters
Date_            20191014
Time                13.35
INSTRUM             spect
PROBHD   5 mm TBO BB-1H
PULPROG            zgigbs
TD                   4096
SOLVENT              C6D6
NS                   6000
DS                      0
SWH             32051.281 Hz
FIDRES           7.825020 Hz
AQ              0.0638976 sec
RG                   1030
DW                 15.600 usec
DE                   6.00 usec
TE                  299.2 K
D1             0.10000000 sec
D11            0.03000000 sec
TD0                     1

======== CHANNEL f1 ========
NUC1                  11B
P1                  10.40 usec
P2                  20.80 usec
PL1        0 dB
PL1W          88.92077637 W
SFO1          160.4615795 MHz

======== CHANNEL f2 ========
CPDPRG[2          waltz16
NUC2                   1H
PCPD2               80.00 usec
PL2                 -0.52 dB
PL12                14.71 dB
PL2W          24.34997177 W
PL12W          0.73029518 W
SFO2          500.1320005 MHz

F2 - Processing parameters
SI                  32768
SF            160.4615790 MHz
WDW                    EM
SSB        0
LB                  10.00 Hz
GB         0
PC                   1.40
```

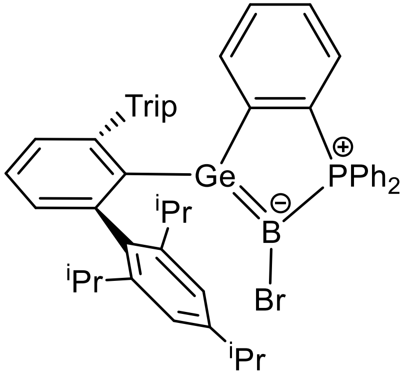

Figure S16. $^{11}B\{^{1}H\}$ NMR spectrum of compound **5**.

NMR spectra of compound **6**.

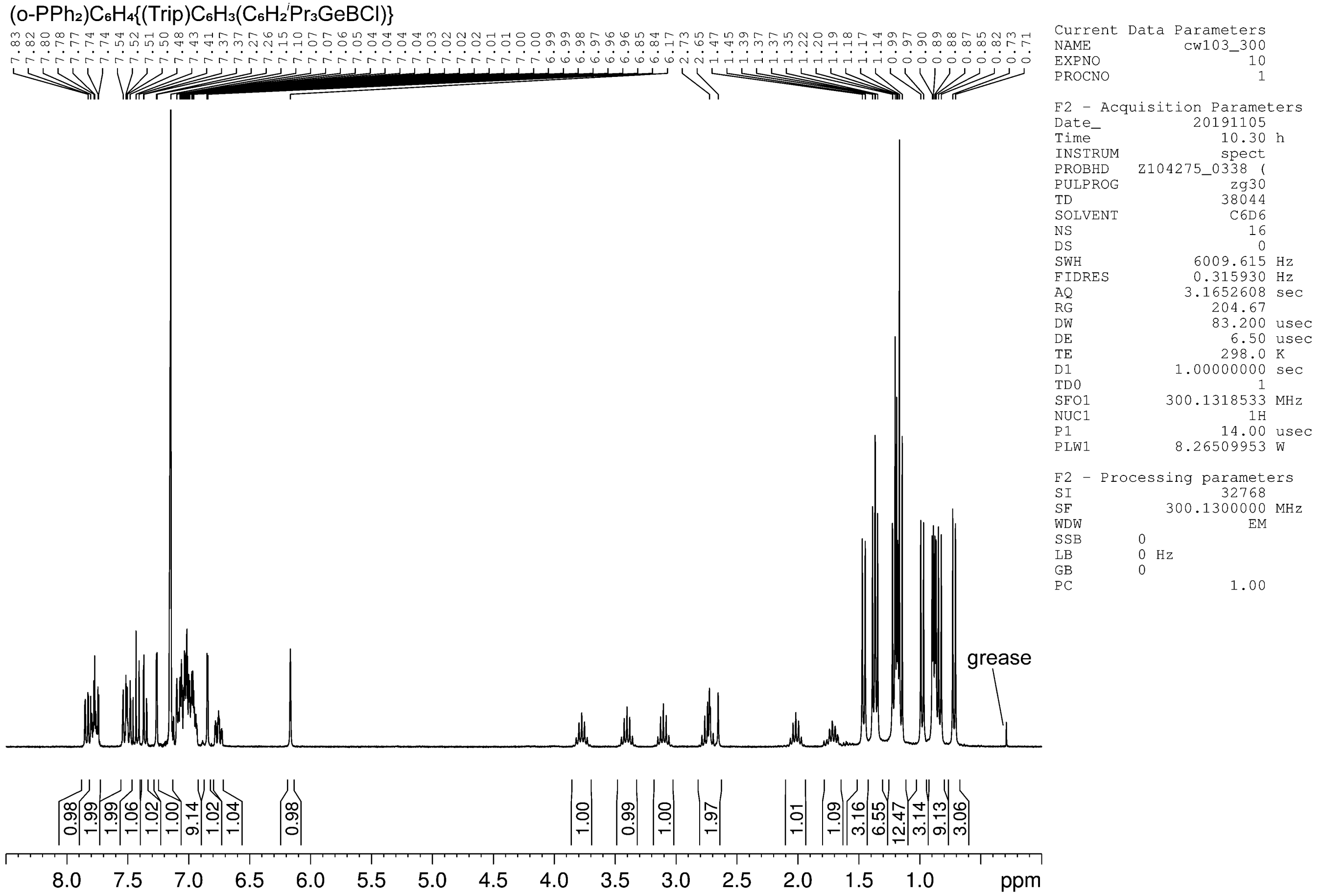

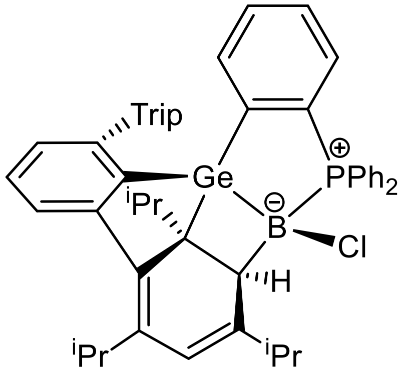

Figure S17. ^{1}H NMR spectrum of compound **6**.

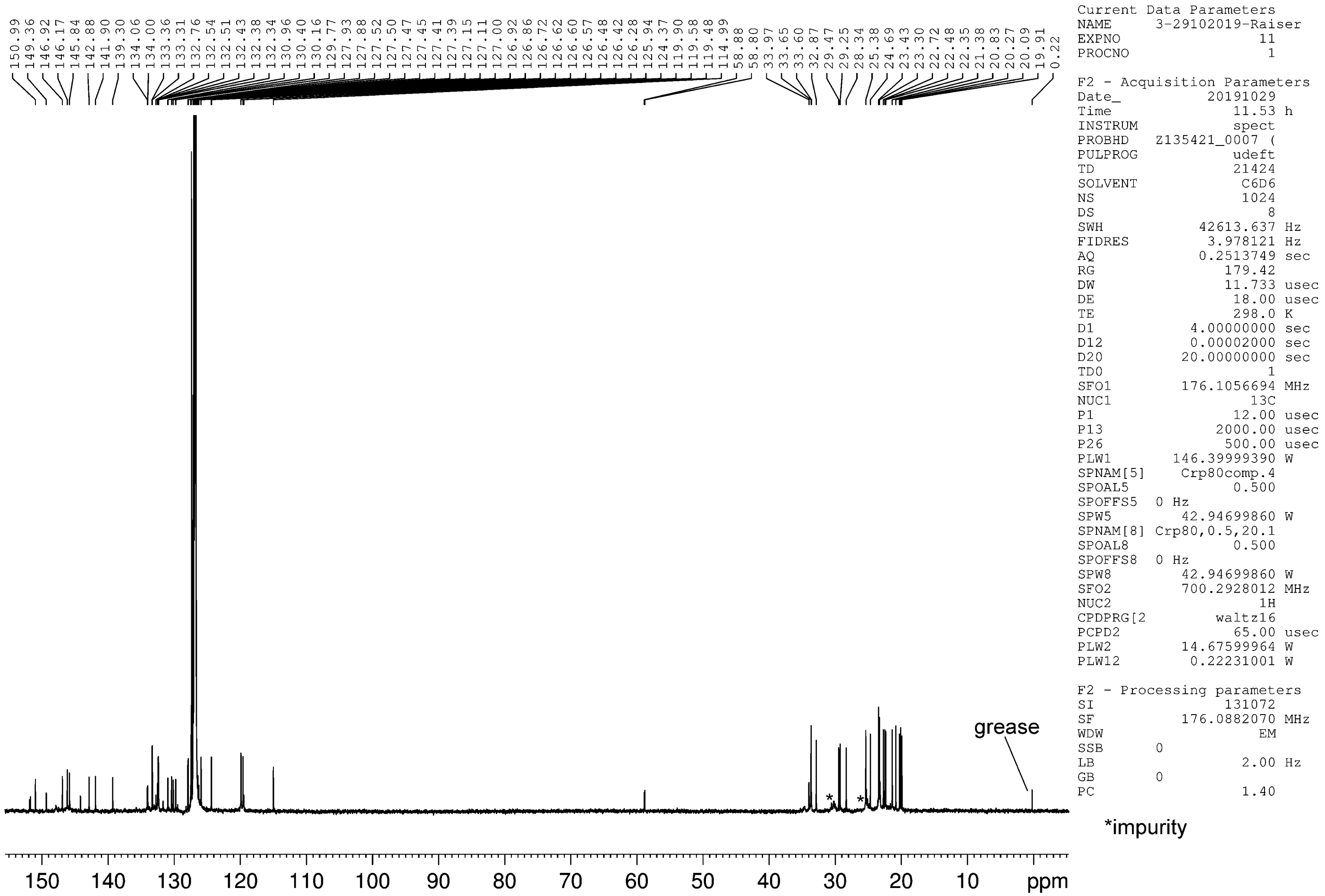

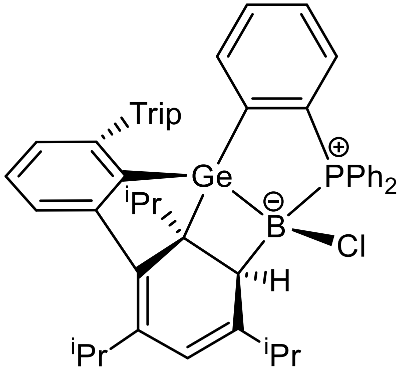

Figure S18. $^{13}C\{^{1}H\}$ NMR spectrum of compound **6.**

(o-PPh₂)C₆H₄{(Trip)C₆H₃(C₆H₂ⁱPr₃GeBCl)}

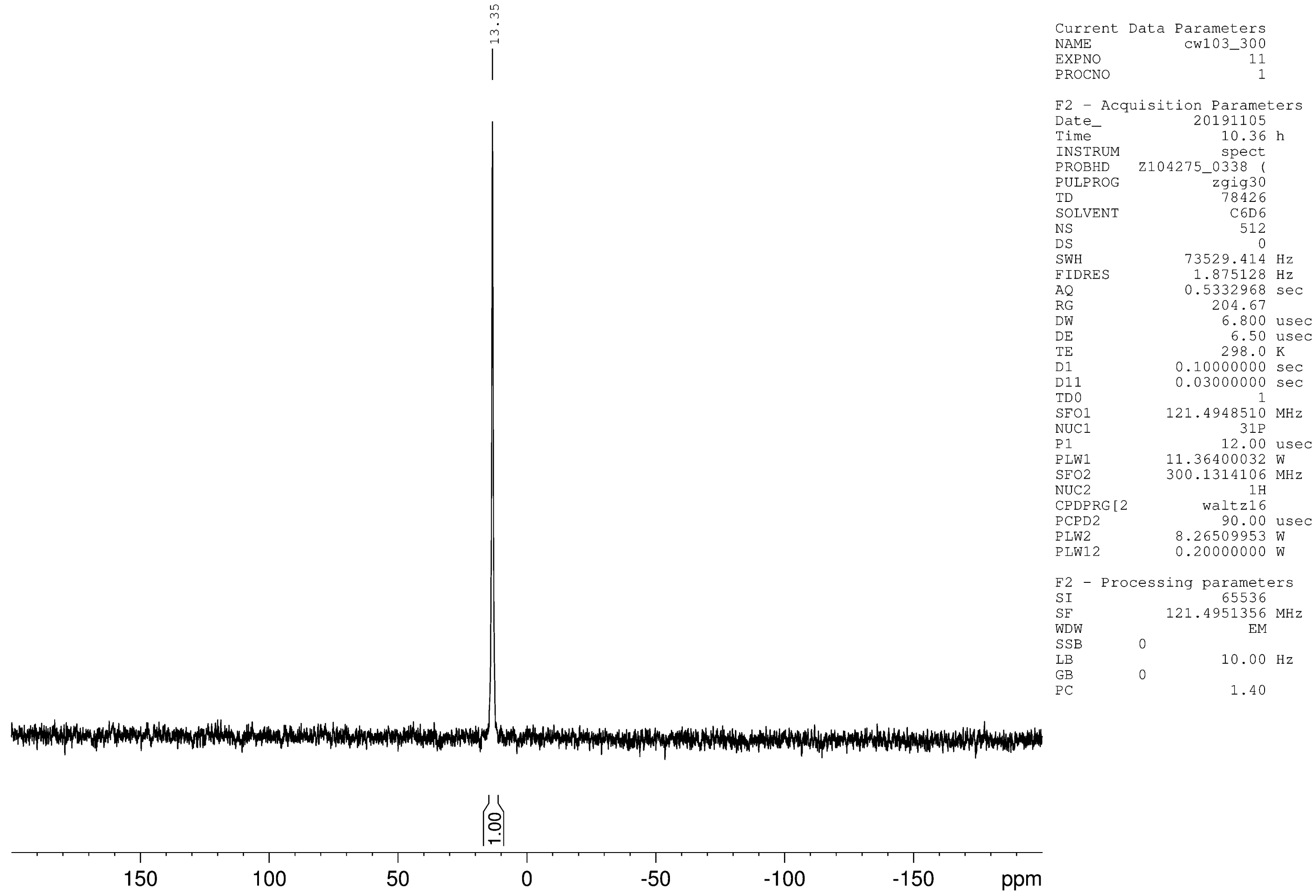

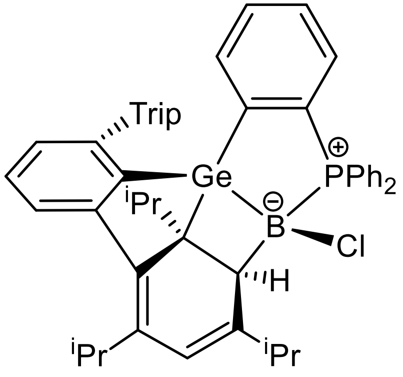

Figure S19. $^{31}P\{^1H\}$ NMR spectrum of compound **6**.

(o-PPh₂)C₆H₄{(Trip)C₆H₃(C₆H₂^{i}Pr₃GeBCl)}

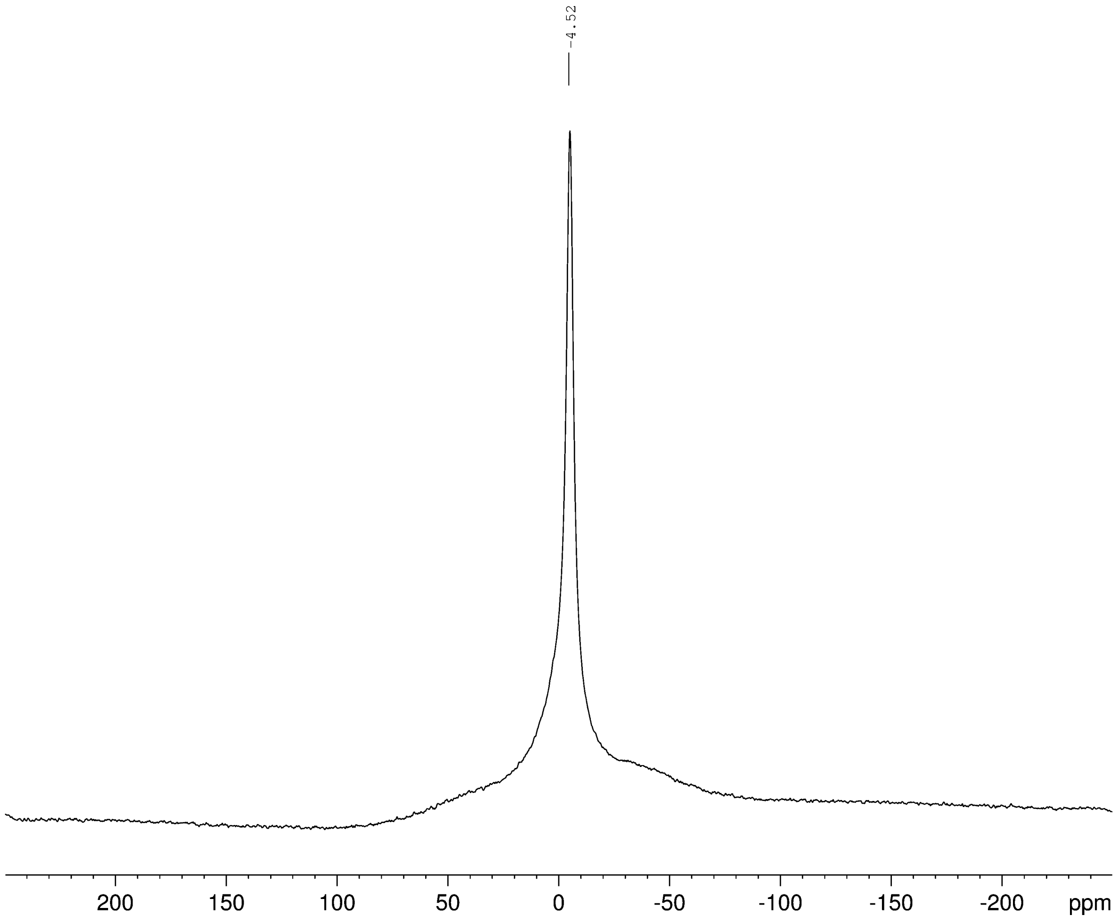


```
Current Data Parameters
NAME              cw103_300
EXPNO                    12
PROCNO                    1

F2 - Acquisition Parameters
Date_              20191105
Time                  10.52 h
INSTRUM               spect
PROBHD    Z104275_0338 (
PULPROG              zgbsig
TD                     8192
SOLVENT                C6D6
NS                     4000
DS                        8
SWH               48076.922 Hz
FIDRES            11.737530 Hz
AQ                0.0851968 sec
RG                   161.31
DW                   10.400 usec
DE                     6.50 usec
TE                    298.0 K
D1               0.10000000 sec
D11              0.03000000 sec
TD0                       1
SFO1             96.2936310 MHz
NUC1                    11B
P1                     5.75 usec
P2                    11.50 usec
PLW1            70.00000000 W
SFO2            300.1314106 MHz
NUC2                     1H
CPDPRG[2            waltz16
PCPD2                 90.00 usec
PLW2             8.26509953 W
PLW12            0.20000000 W

F2 - Processing parameters
SI                    32768
SF               96.2936310 MHz
WDW                      EM
SSB      0
LB                    50.00 Hz
GB       0
PC                     1.40
```

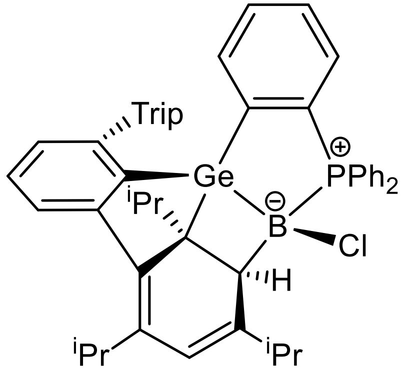

Figure S20. $^{11}B\{^{1}H\}$ NMR spectrum of compound **6**.

NMR spectra of compound **7**.

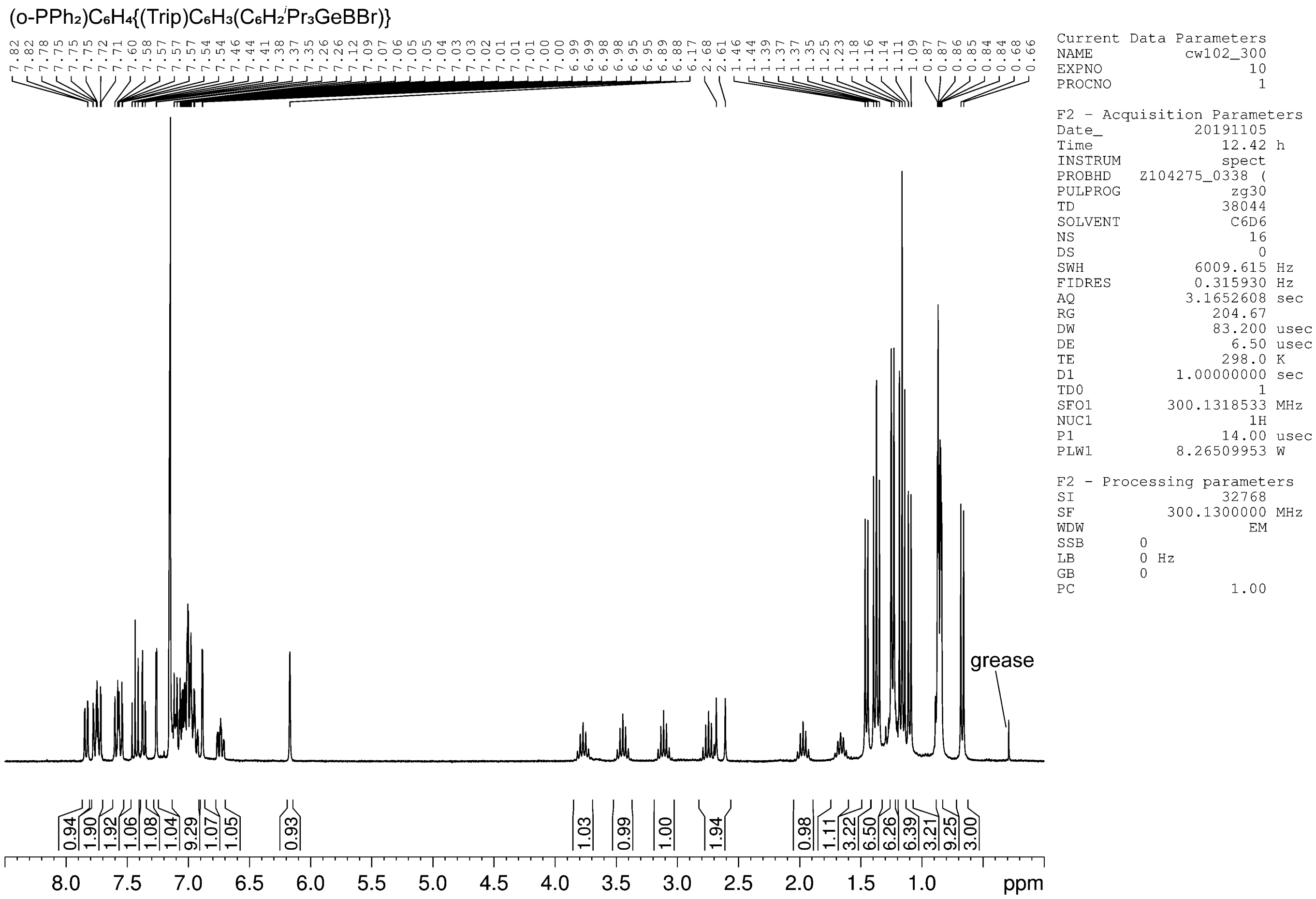

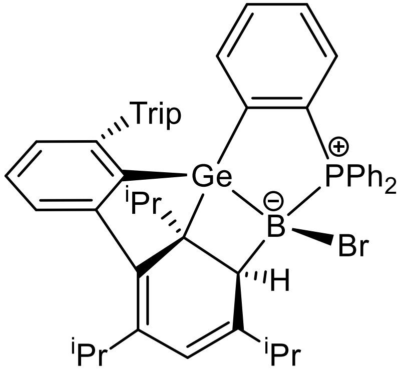

Figure S21. ^{1}H NMR spectrum of compound **7**.

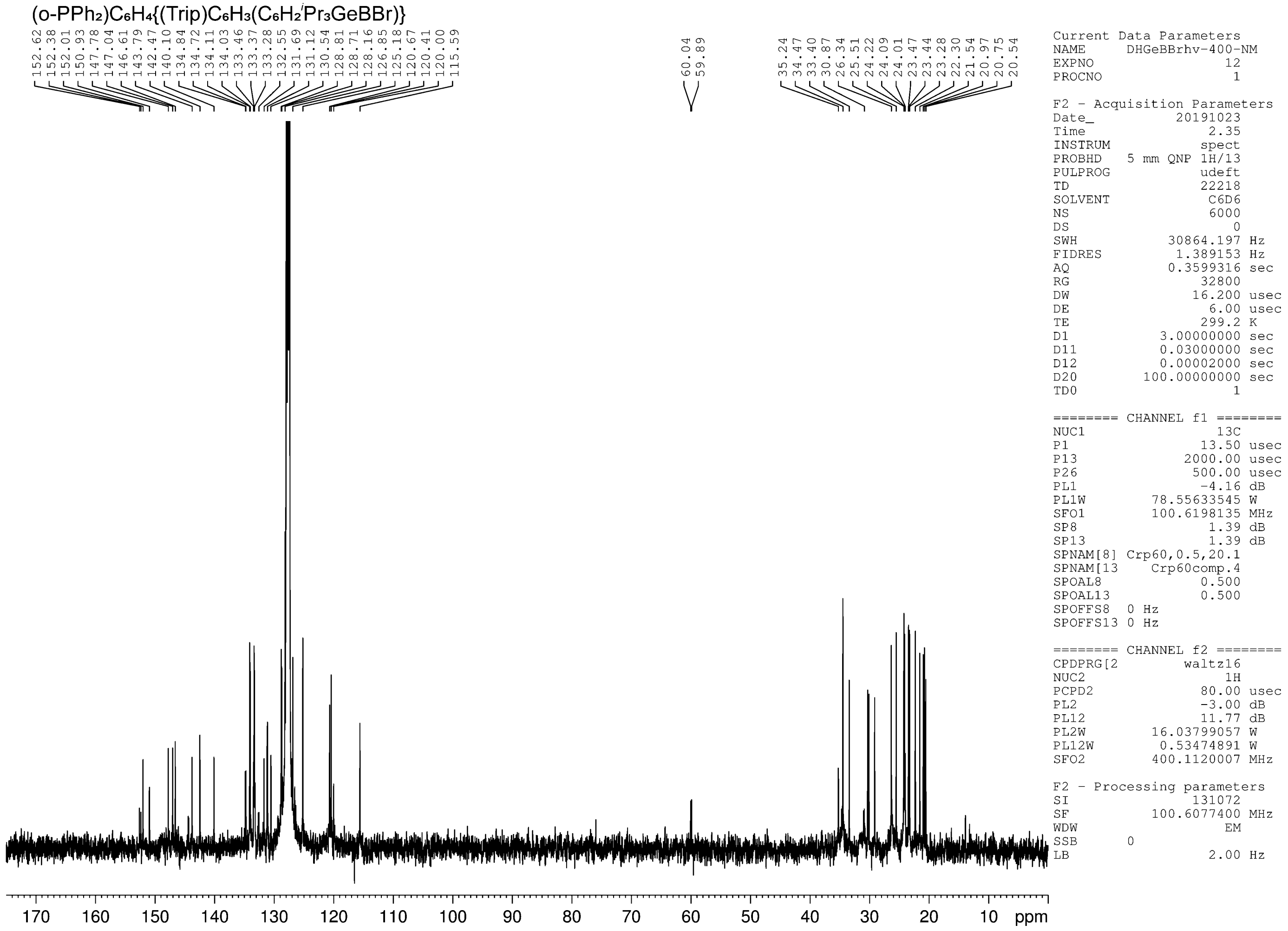

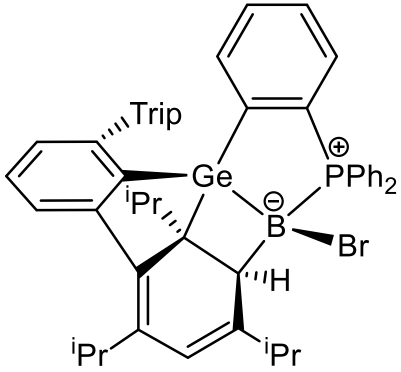

Figure S22. $^{13}C\{^{1}H\}$ NMR spectrum of compound **7.**

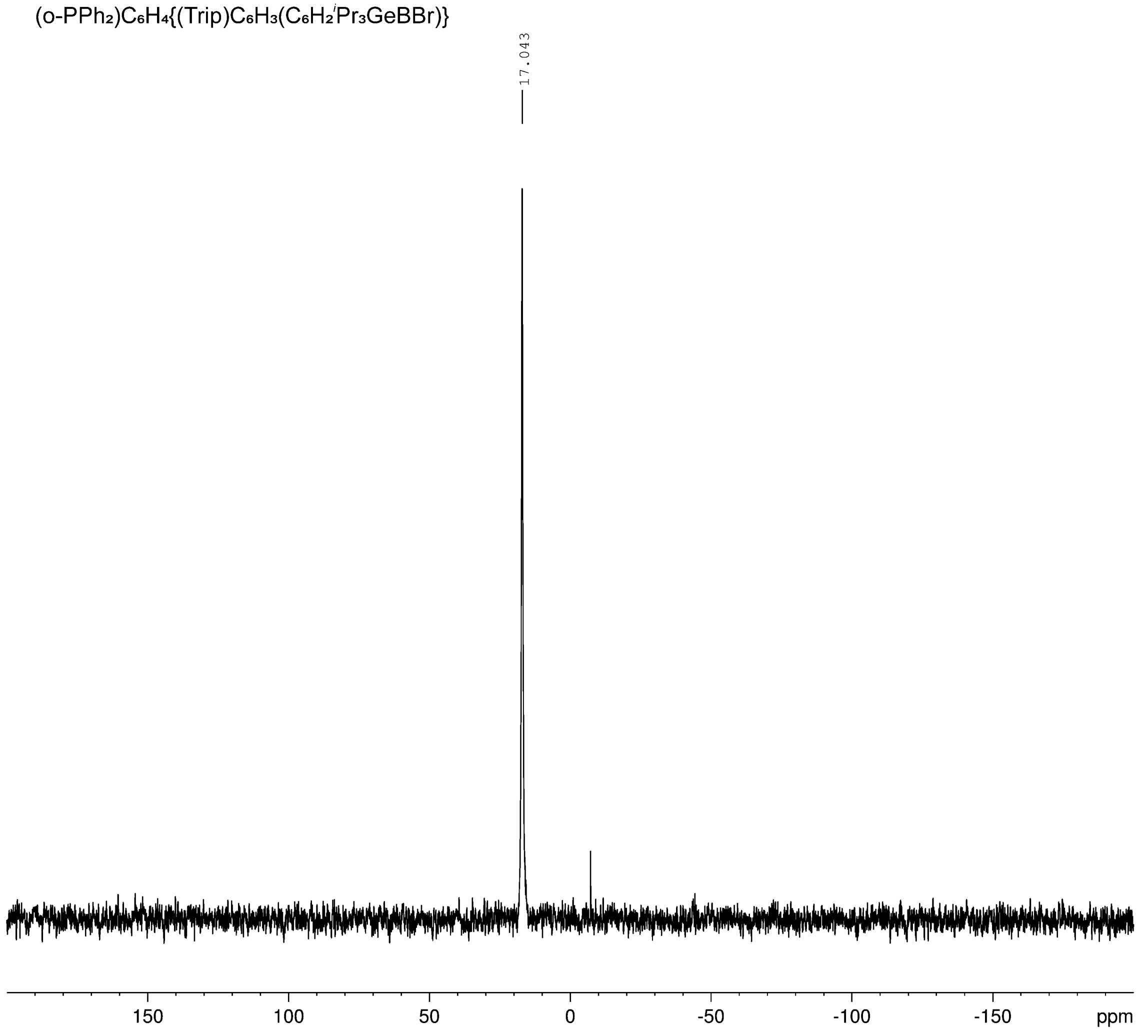


```
Current Data Parameters
NAME              cw98_300
EXPNO                   21
PROCNO                   1

F2 - Acquisition Parameters
Date_             20191030
Time                 13.50 h
INSTRUM              spect
PROBHD   Z104275_0338 (
PULPROG            zgig30
TD                   78426
SOLVENT               C6D6
NS                     512
DS                       0
SWH             73529.414 Hz
FIDRES           1.875128 Hz
AQ              0.5332968 sec
RG                  204.67
DW                   6.800 usec
DE                    6.50 usec
TE                   298.0 K
D1             0.10000000 sec
D11            0.03000000 sec
TD0                      1
SFO1        121.4948510 MHz
NUC1                   31P
P1                   12.00 usec
PLW1         11.36400032 W
SFO2        300.1314106 MHz
NUC2                    1H
CPDPRG[2          waltz16
PCPD2                90.00 usec
PLW2          8.26509953 W
PLW12         0.20000000 W

F2 - Processing parameters
SI                   65536
SF          121.4948510 MHz
WDW                     EM
SSB      0
LB                   10.00 Hz
GB       0
PC                    1.40
```

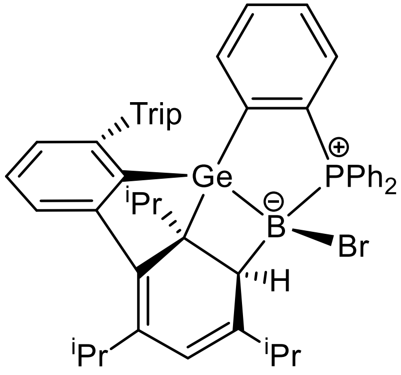

Figure S23. $^{31}P\{^{1}H\}$ NMR spectrum of compound **7**.

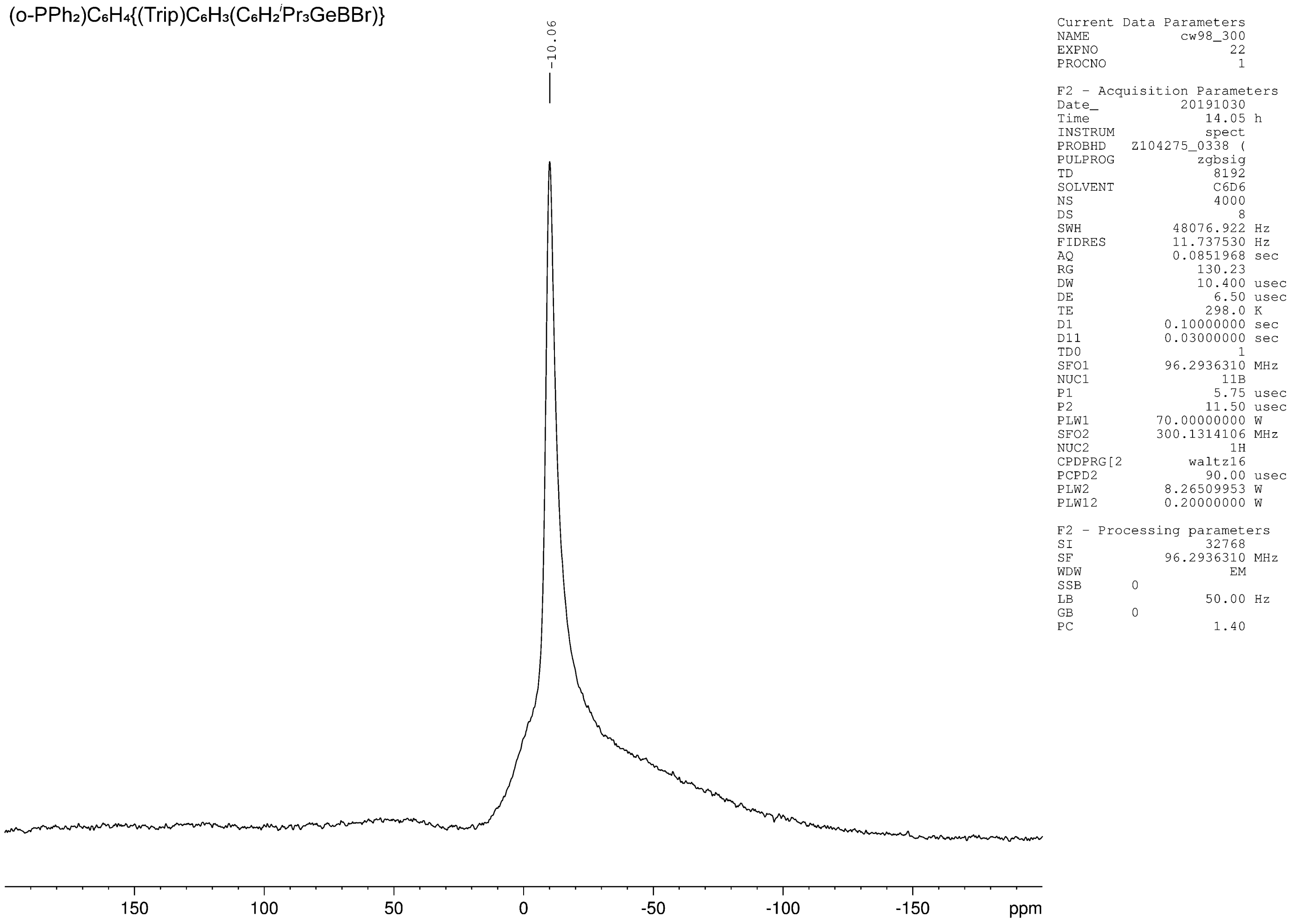

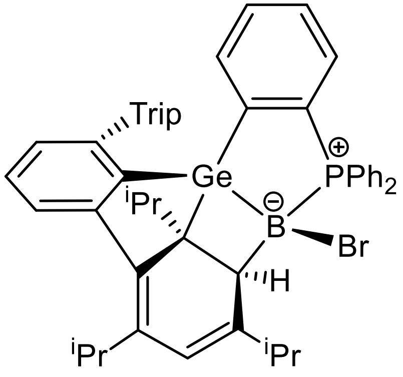

Figure S24. $^{11}B\{^{1}H\}$ NMR spectrum of compound **7**.

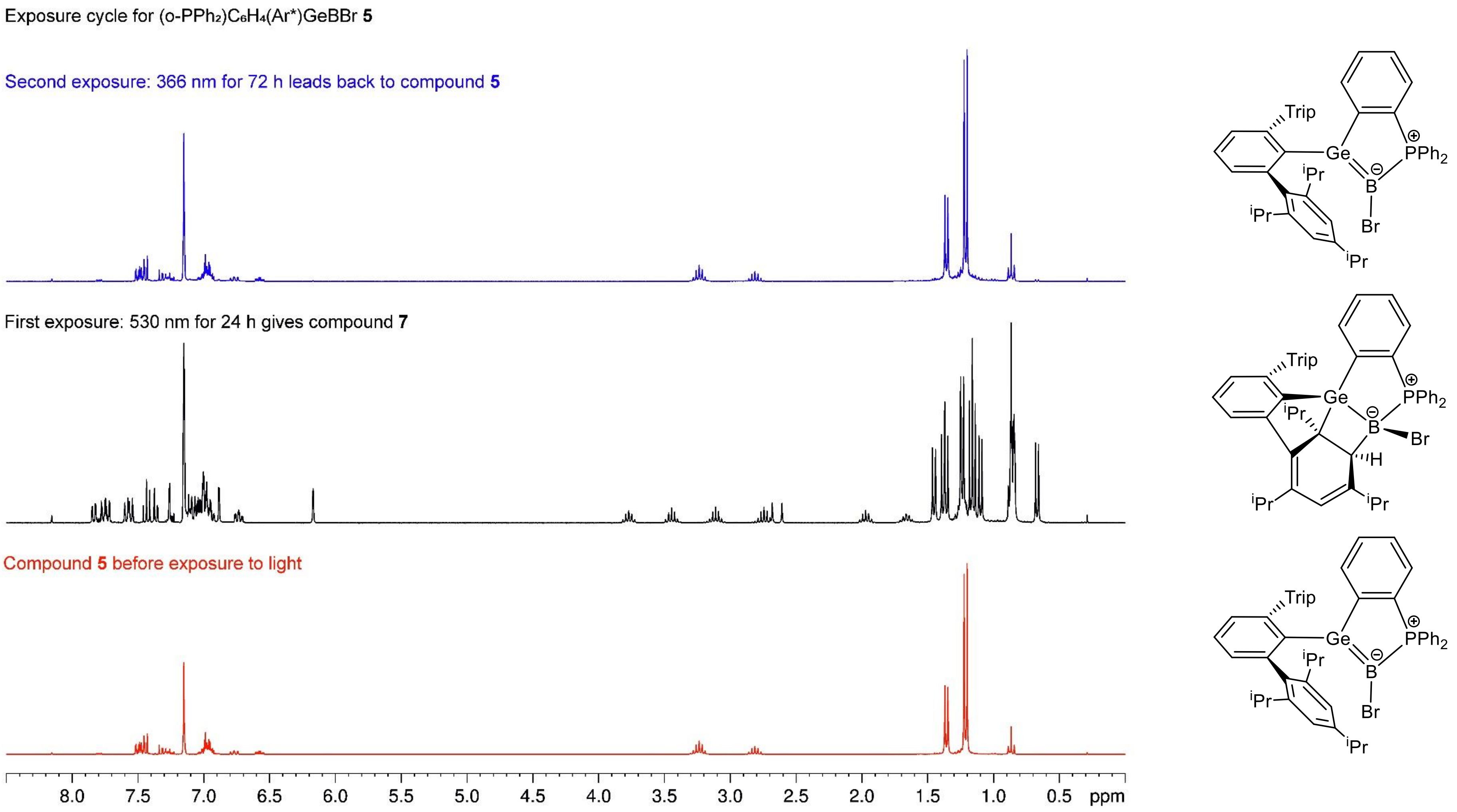

Figure S25. ^{1}H NMR spectra of reversible cycloaddition from **5** to **7** and reconversion to **5**.

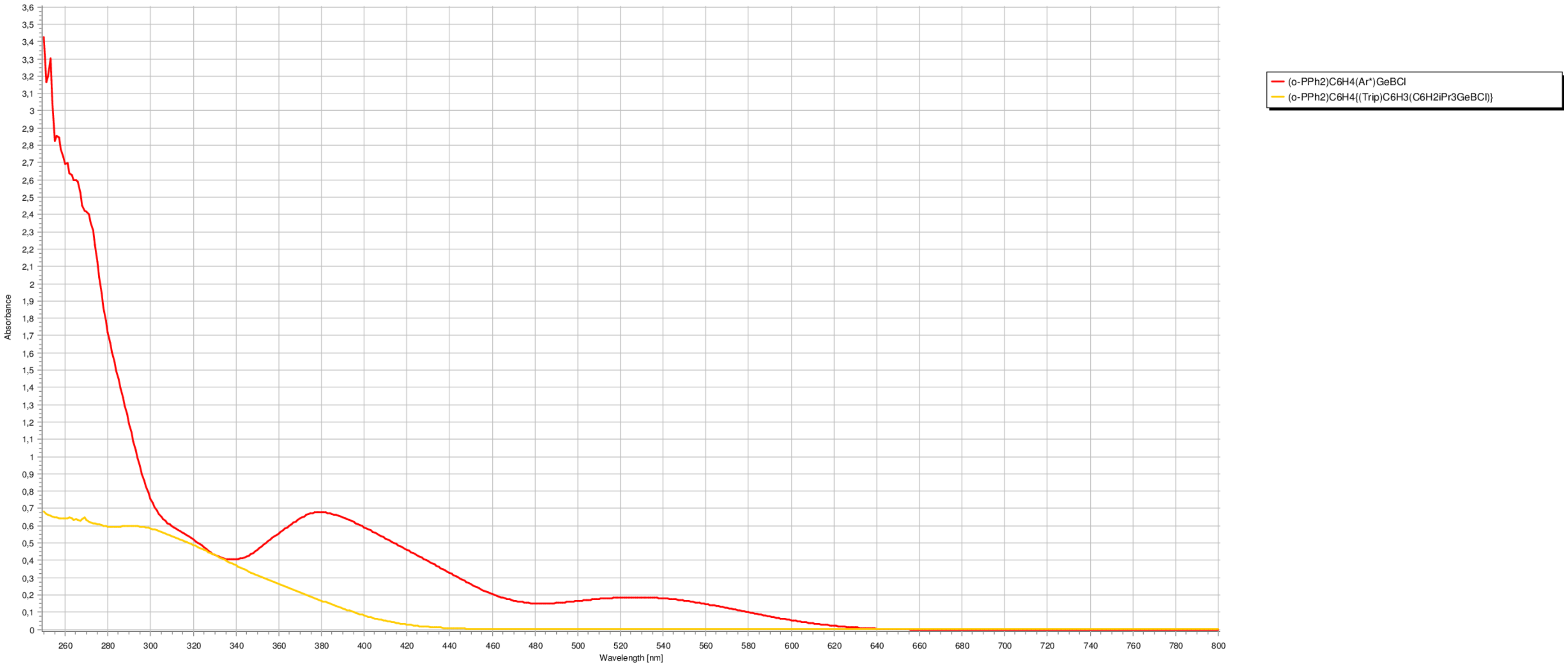

Figure S26. UV-Vis spectra of **4** (red, 1.68 x 10^{-4} mol/L) and **6** (yellow, 7.11 x 10^{-5} mol/L).

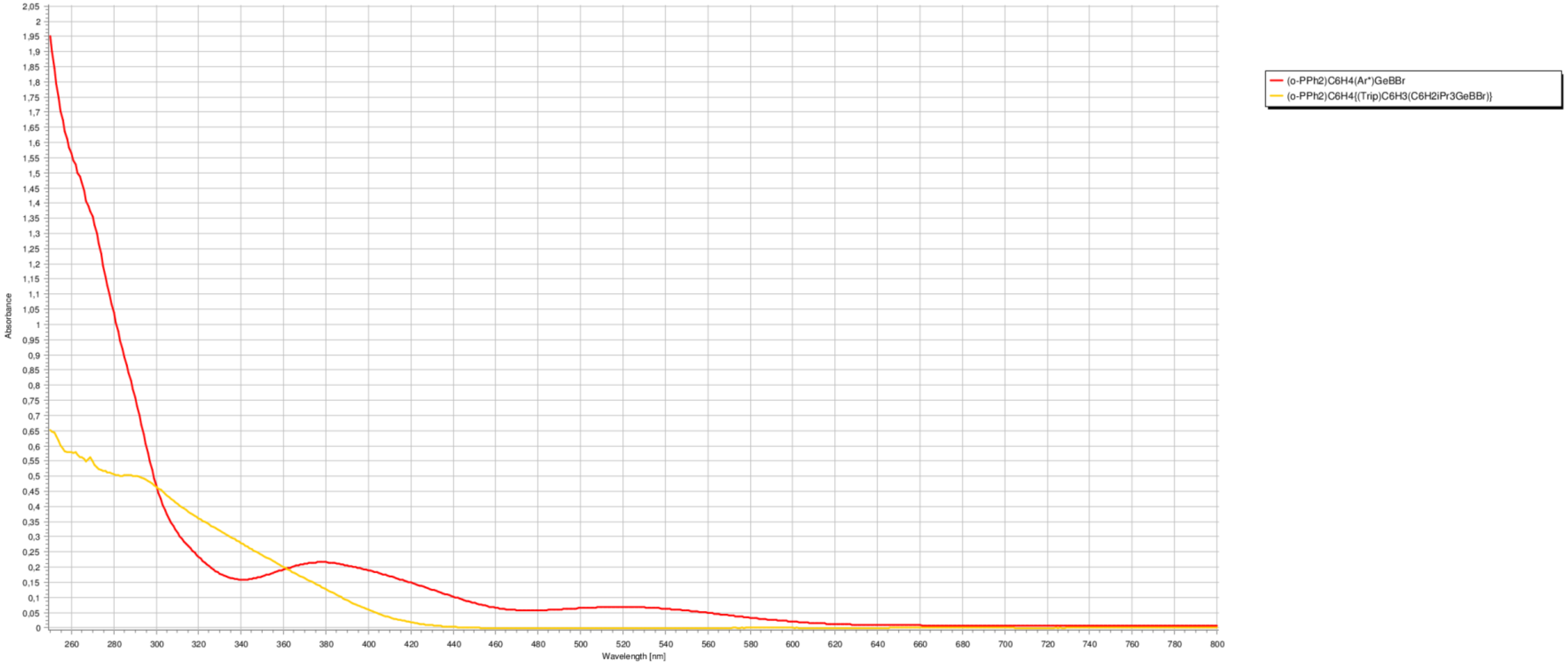

Figure S27. UV-Vis spectra of **5** (red, 1.34 x 10^{-4} mol/L) and **7** (yellow, 9.0 x 10^{-5} mol/L).

Table S1. Results of UV-Vis measurements.

Compound	Concentration [mol·L^{-1}]	Absorption max. [nm]	Absorbance	mol. Absorptivity [L·mol^{-1}·cm^{-1}]
**4**	1.68·10^{-4}	379.3	0.6804	4.05·10^{3}
	1.68·10^{-4}	527.8	0.1877	1.12·10^{3}
**5**	1.34·10^{-4}	378.5	0.2017	1.51·10^{3}
	1.34·10^{-4}	520.3	0.0635	4.74·10^{2}
**6**	0.71·10^{-4}	290.9	0.5996	8.43·10^{3}
**7**	0.9·10^{-4}	287.0	0.5026	5.58·10^{3}

Crystallography

X-ray data were collected with a Bruker Smart APEX II diffractometer with graphite-monochromated Mo Kα radiation or a Bruker APEX II Duo diffractometer with a Mo IµS microfocus tube and TRIUMPH monochromator. The programs used were Bruker's APEX2 v2011.8-0, including SADABS for absorption correction, SAINT for data reduction and SHELXS for structure solution, as well as the WinGX suite of programs version 1.70.01 or the GUI ShelXle, including SHELXL for structure refinement.[3]

Table S2. Selected crystallographic data for compound **2**-**5**.

	**2**	**3**	**4**	**5**
Empirical formula	$C_{54}H_{63}BCl_3GeP \cdot 2\ C_7H_{18}$	$C_{54}H_{63}BBr_3GeP$	$C_{54}H_{63}BClGeP$	$C_{54}H_{63}BBrGeP \cdot C_6H_{14}$
M [g/mol]	1117.03	1066.14	861.86	992.5
T [K]	100(2)	100(2)	100(2) K	100(2)
λ [Å]	0.71073	0.71073	0.71073 Å	0.71073
Crystal system	Triclinic	Monoclinic	Triclinic	Triclinic
Space group	P-1	C2/c	P-1	P-1
Z	2	8	2	2
a [Å]	10.4470(3)	26.8698(5)	9.1445(2)	10.0921(5)
b [Å]	16.4339(5)	19.1916(4)	13.3471(3)	17.7847(9)
c [Å]	17.8021(6)	20.7644(4)	20.0750(4)	18.3358(9)
α [°]	83.679(2)	90	76.8550(10)	118.287(2)
β [°]	88.301(2)	109.9780(10)	77.4760(10)	99.387(3)
γ [°]	83.878(2)	90	82.1960(10)	91.300(3)
V [Å^3]	3019.95(16)	10063.3(3)	2319.57(9)	2840.2(3)
D_c [g/cm^3]	1.228	1.407	1.234	1.161
μ [mm^{-1}]	0.706	3.057	0.788	1.305
F(000)	1180	4352	912	1048
Crystal size [mm]	0.14 x 0.13 x 0.11	0.18 x 0.17 x 0.15	0.22 x 0.20 x 0.17	0.18 x 0.15 x 0.13
θ range [°]	1.151 - 26.384	2.977 - 27.521	3.134 - 29.148	3.112 - 27.191
Limiting indices	-13<=h<=12	-34<=h<=34	-12<=h<=12	-12<=h<=12
	-20<=k<=20	-24<=k<=24	-18<=k<=18	-22<=k<=22
	-21<=l<=22	-27<=l<=26	-27<=l<=27	-23<=l<=23
Reflections coll.	30483	69079	71000	45944
Independent refl.	11508	11561	12436	12530
R_{int}	0.0500	0.0385	0.0216	0.0469
completeness	95.1	99.8	99.6	99.6
Absorption correction	multi-scan	multi-scan	multi-scan	multi-scan
Min. Max. transmis.	0.7454, 0.6628	0.7456, 0.6336	0.7458, 0.7043	0.7455, 0.6720
Parameters/restraints	697/0	553/0	535/0	523/0
R_1, wR_2 [I>2σI]	0.0533, 0.1319	0.0243, 0.0516	0.0325, 0.0865	0.0439, 0.1118
R_1, wR_2 (all data)	0.0926, 0.1775	0.0361, 0.0551	0.0382, 0.0895	0.0668, 0.1210
GooF	1.103	1.014	1.066	1.028
peak / hole [eÅ^{-3}]	0.997/-1.305	0.516/-0.288	1.097/-0.367	1.072/-0.847
Flack				
CCDC	1964301	1964298	1964297	1964300

Table S3. Selected crystallographic data for compound **6**, **7**.

	**6**	**7**
Empirical formula	$C_{57}H_{70}BClGeP$	$C_{54}H_{63}BBrGeP$
M [g/mol]	904.95	906.32
T [K]	100(2)	100(2) K
λ [Å]	0.71073	0.71073 Å
Crystal system	Monoclinic	Monoclinic
Space group	$P2_1/n$	$P2_1$
Z	4	2
a [Å]	10.4043(4)	10.7424(3)
b [Å]	17.7792(6)	18.1923(4)
c [Å]	26.5273(9)	12.7532(3)
α [°]	90	90
β [°]	98.114(2)	109.3410(10)
γ [°]	90	90
V [$Å^3$]	4857.9(3)	2351.69(10)
D_c [g/cm^3]	1.237	1.280
μ [mm^{-1}]	0.755	1.570
F(000)	1924	948
Crystal size [mm]	0.21 x 0.17 x 0.15	0.18 x 0.17 x 0.15
θ range [°]	2.601 – 30.078	3.109 - 27.141
Limiting indices	-14<=h<=14	-13<=h<=13
	-25<=k<=25	-23<=k<=21
	-37<=l<=37	-16<=l<=16
Reflections coll.	143499	25190
Independent refl.	14212	9965
R_{int}	0.0372	0.0419
completeness	99.8	99.6
Absorption correction	multi-scan	multi-scan
Min. Max. transmis.	0.7460, 0.6659	0.7455, 0.6607
Parameters/restraints	562/3	529/1
R_1, wR_2 [I>2σI]	0.0353, 0.0866	0.0402, 0.0741
R_1, wR_2 (all data)	0.0444, 0.0909	0.0520, 0.0774
GooF	1.083	1.039
peak / hole [$eÅ^{-3}$]	0.638/-0.538	1.058/-0.605
Flack		0.032(4)
CCDC	1964299	1964296

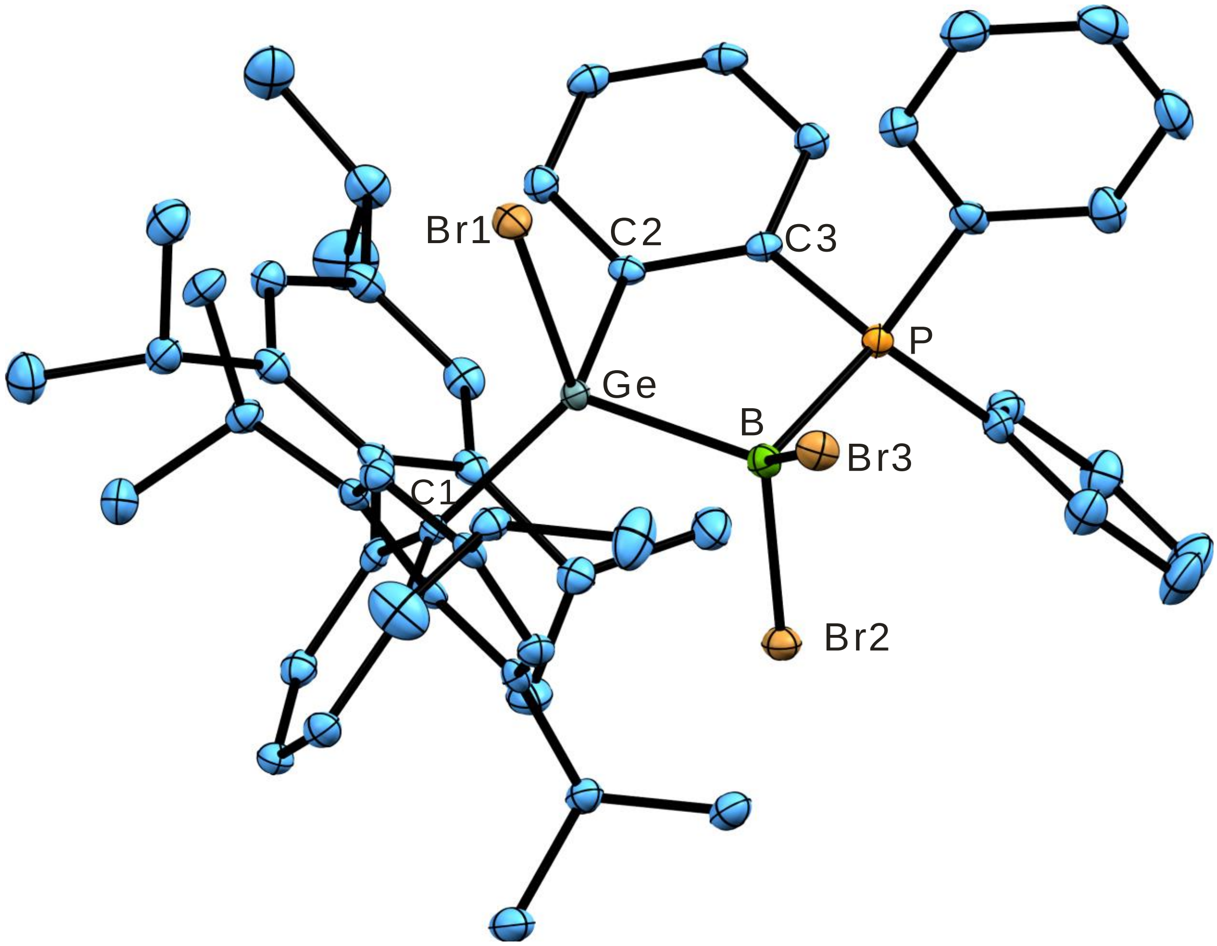

Figure S28. ORTEP of the molecular structure of **3**. Ellipsoids at 50% probability. Hydrogen atoms are omitted for clarity. Interatomic distances [Å] and angles [°]: Ge–B 2.089(2), Ge–Br1 2.3708(2), Ge–C1 1.9981(18), Ge–C2 1.9838(13), B–P 1.979(2), B–Br2 2.019(2), B–Br3 2.004(2), C2–C3 1.404(3), Ge–B–P 101.3(1), C2–Ge–B 98.3(1), B–P–C3 105.1(1), P–C3–C2 119.2(1), C3–C2–Ge 115.3(1), B–Ge–Br1 1 109.1(1), B–Ge–C2 98.3(1), P–B–Br2 106.7(1), P–B–Br3 109.6(1).

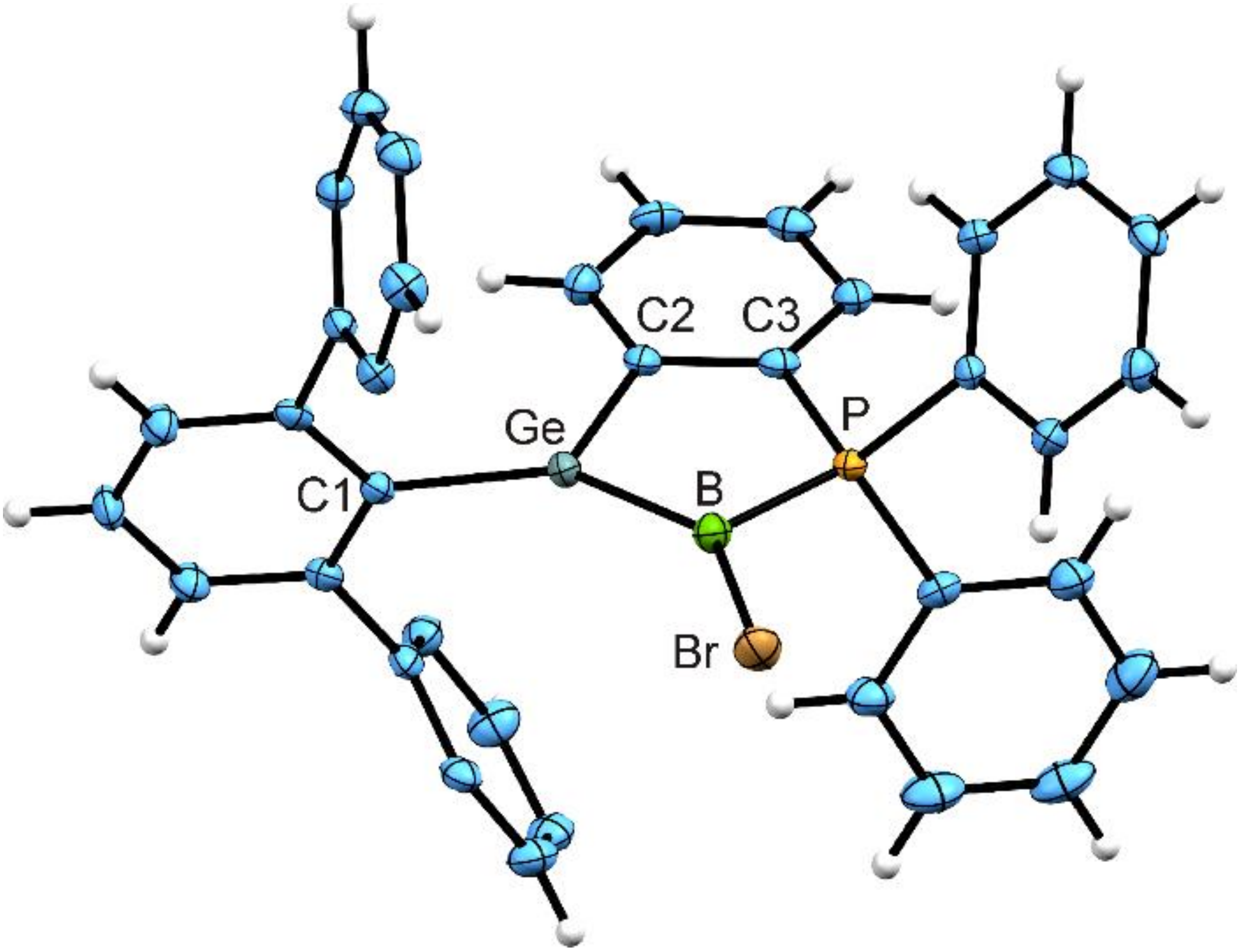

Figure S29. ORTEP of the molecular structure of **5**. Ellipsoids at 50% probability. ^{i}Pr groups of the aryl substituents are omitted for clarity. Interatomic distances [Å] and angles [°]: Ge–B 1.895(3), Ge–C1 1.949(3), Ge–C2 1.959(3), B–P 1.878(3), B–Br 1.958(3), C2–C3 1.403(4), C3–P 1.810(3), C1–Ge–C2 114.9(1), C2–Ge–B 101.7(1), C1–Ge–B 143.0(1), Ge–B–P 103.1(2), B–P–C3 116.9(1), P–C3–C2 115.2(2), C3–C2–Ge 113.4(2), Br–B–Ge 140.0(2), Br–B–P 116.8(2).

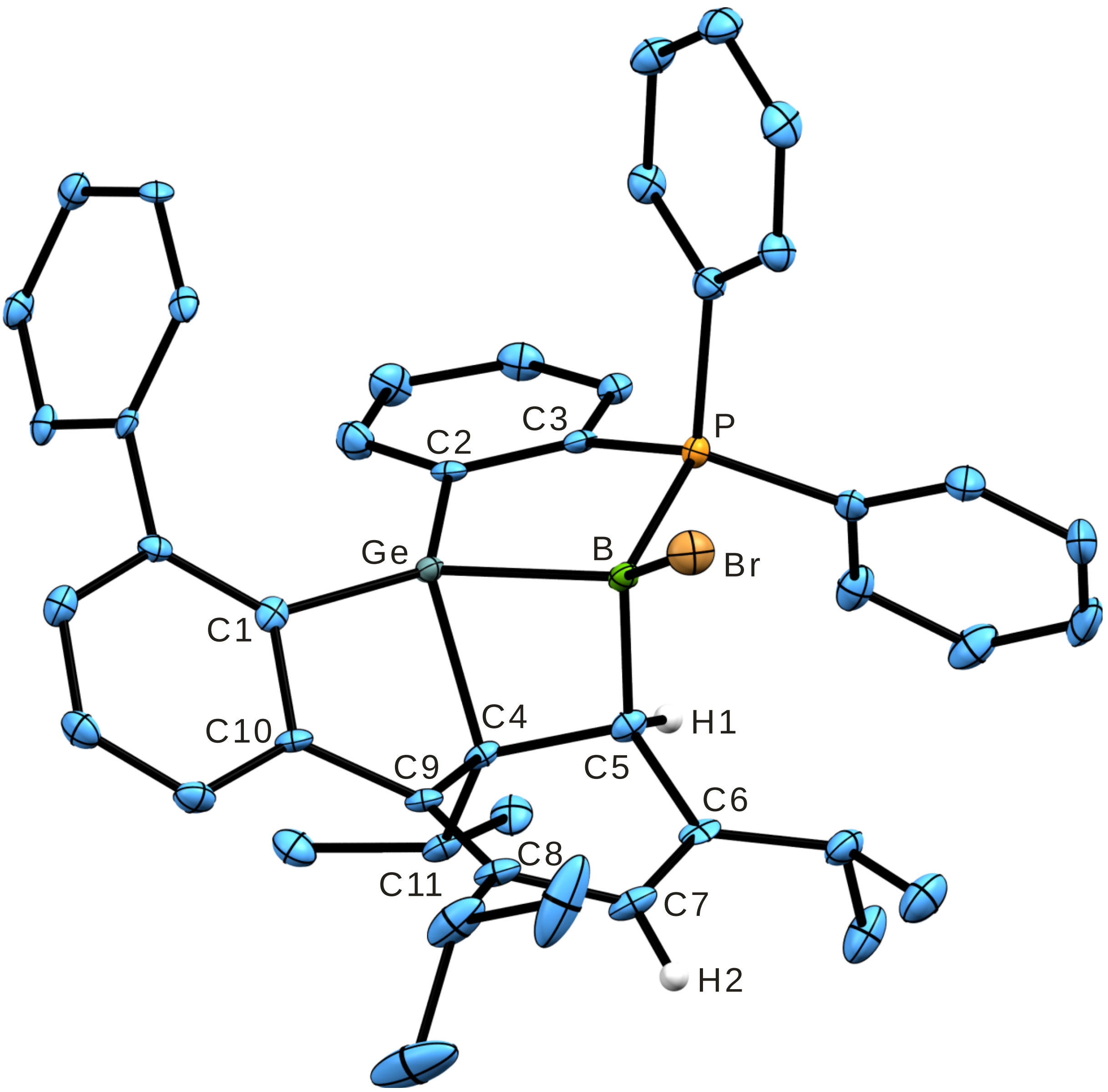

Figure S30. ORTEP of the molecular structure of **7**. Ellipsoids at 50% probability. ^{i}Pr groups of the aryl substituents are omitted for clarity. Interatomic distances [Å] and angles [°]:Ge–B 2.111(5), B–P 1.950(5), P–C3 1.826(4), C2–C3 1.402(6), Ge–C2 1.961(4), Ge–C1 1.939(4), Ge–C4 1.985(4), B–C5 1.662(7), B–Br 2.005(5), C4–C5 1.547(6), C4–C9 1.549(6), C9–C10 1.499(6), C1–C10 1.419(6), C5–C6 1.499(6), C6–C7 1.337(6), C7–C8 1.474(6), C8–C9 1.340(6), C1–Ge-C2 129.6(2), C1–Ge-B 128.1(2), C1–Ge–C4 92.3(2), C2–Ge–B 101.0(2), C4–Ge–B 74.4(2), C2–Ge–C4 113.5(2), C5–B–P 110.0(3), Ge–B–P 97.5(2), C5–B–Ge 86.4(3), Br–B–Ge 128.2(2), Br–B–P 113.1(2), Br–B–C5 117.8(3), B–P–C3 106.0(2), C3–C2–Ge 113.0(3), P–C3–C2 117.1(3), C10–C1–Ge 105.8(3), C4–C9–C10 113.4(3), C9–C10–C1 116.0(3), Ge–C4–C9 96.6(3), C4–C5–C6 113.0(3), C4–C5–B 101.1(3), C5–C6–C7 117.9(4), C6–C7–C8 122.6(4), C7–C8–C9 119.3(4), C8–C9–C4 119.0(4).

Computational Details

Structure Optimisation, NBO, NRT analyses

DFT calculations were carried out with Gaussian09.[4] The molecular structures of (*o*-PPh_2)C_6H_4(Ar*)Ge(BCl) (**4**), (*o*-PPh_2)C_6H_4(Ar*)Ge(BBr) (**5**) and products of 2+2 cycloaddition **6** and **7** were optimised using the BP86 functional, along with the implemented def2TZVP basis sets

for Ge, Br, Cl; P, C and H atoms.[5] Dispersion corrections were included by adding the D3 version of Grimme´s dispersion with Becke-Johnson damping.[6] The geometry optimization was performed without imposing any symmetry constraints, and the structure obtained was confirmed as a true minimum by calculating analytical frequencies, which gave one imaginary frequency (**5**: i15.5 cm^{-1}). Plots were generated with the software Chemcraft.[7]

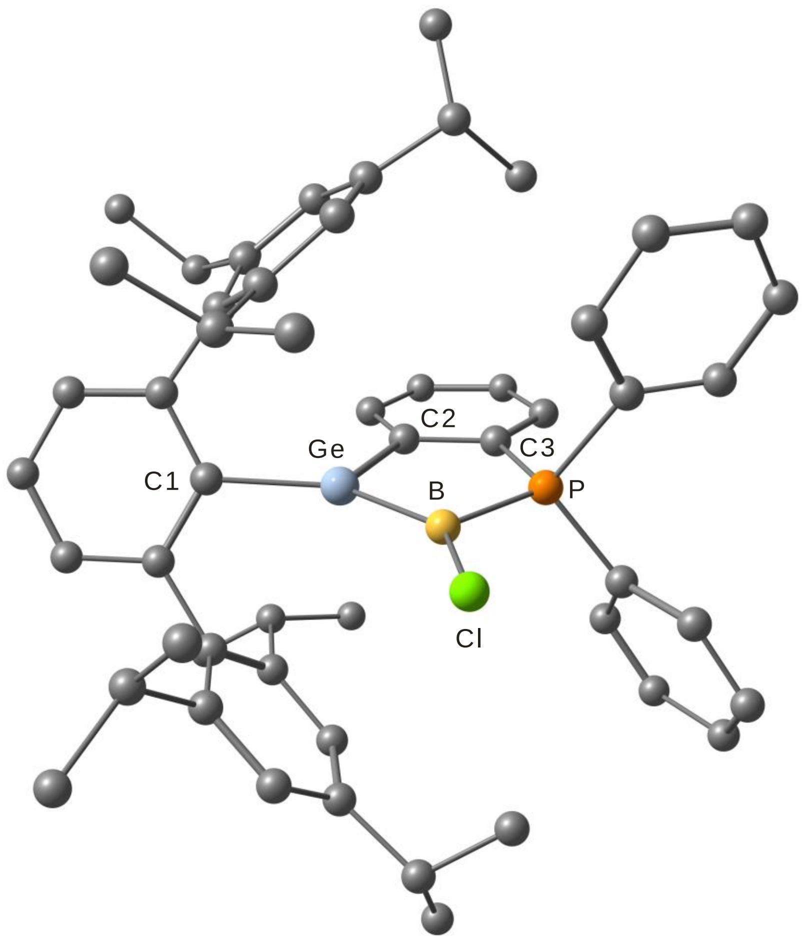

Figure S31. Optimized molecular structure **4**.

Table S4. Selected distances and angles of **4**, measured (molecular structure in the solid state) and calculated values

distance/angle	measured Å / °	calculated Å / °
Ge–B	1.886(2)	1.910
Ge–C1	1.944(1)	1.960
Ge–C2	1.948(1)	1.956
B–P	1.888(2)	1.873
C2–C3	1.411(2)	1.412
B–Cl	1.786(2)	1.790
C3–P	1.811(1)	1.814
C1–Ge–C2	112.9(1)	112.6
C2–Ge–B	102.0(1)	102.4
Ge–B–P	103.1(1)	102.1

The summary of the natural population analysis results with natural charges of 1.09 for Ge, –0.68 for B, 1.29 for P, –0.16 for Cl.

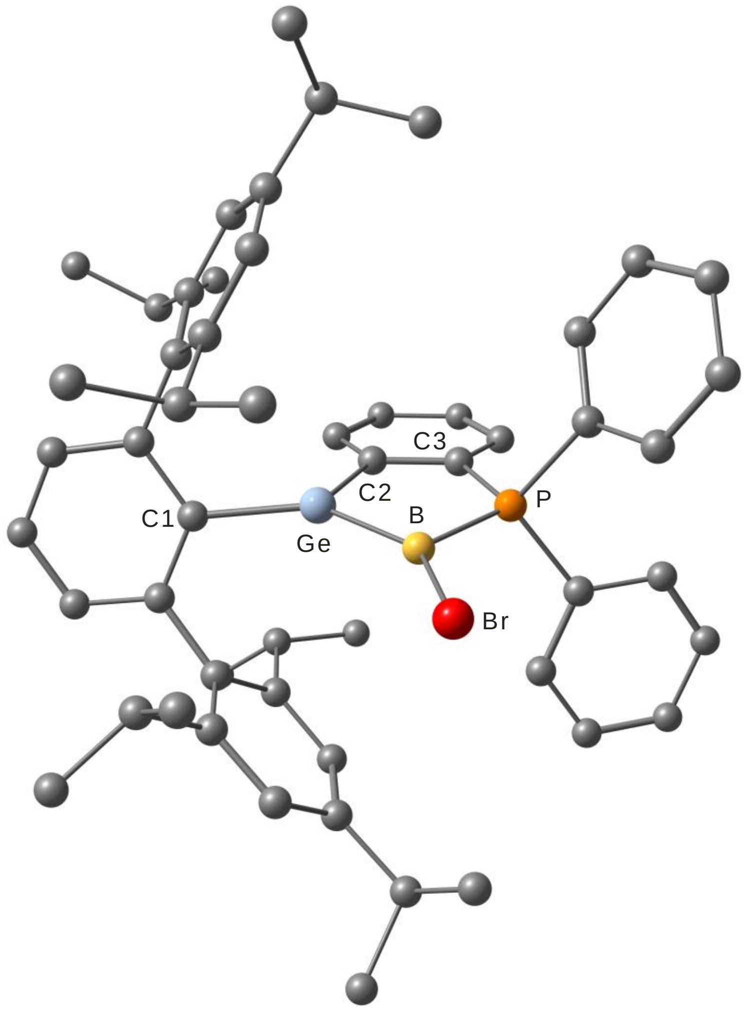

Figure S32. Optimized molecular structure **5**.

Table S5. Selected distances and angles of **5**, measured (molecular structure in the solid state) and calculated values

distance/angle	measured Å / °	calculated Å / °
Ge–B	1.895(3)	1.904
Ge–C1	1.949(3)	1.953
Ge–C2	1.959(3)	1.956
B–P	1.878(3)	1.874
C2–C3	1.403(4)	1.407
B–Br	1.958(3)	1.946
C3–P	1.810(3)	1.818
C1–Ge–C2	114.9(1)	112.0
C2–Ge–B	101.7(1)	102.2
Ge–B–P	103.1(2)	102.4

The summary of the natural population analysis results with natural charges of 1.10 for Ge, –0.77 for

B, 1.30 for P, –0.08 for Br. [8]

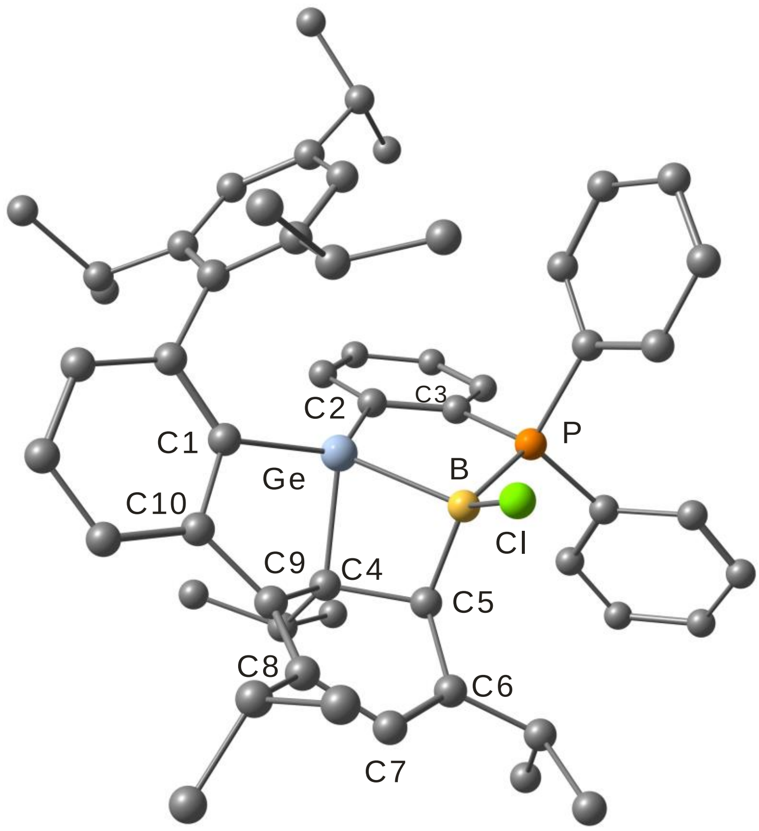

Figure S33. Optimized molecular structure **6**.

Table S6. Selected distances and angles of **6**, measured (molecular structure in the solid state) and calculated values

distance/angle	measured Å / °	calculated Å / °
Ge–B	2.1229(16)	2.118
Ge–C1	1.9393(14)	1.934
Ge–C2	1.9509(14)	1.959
B–P	1.9766(16)	1.952
C2–C3	1.407(2)	1.412
B–Cl	1.8395(16)	1.836
C3–P	1.8227(14)	1.827
B–C5	1.663(2)	1.664
C4–C5	1.5622(19)	1.552
C5–C6	1.5098(19)	1.493
C6–C7	1.342(2)	1.352
C7–C8	1.471(2)	1.465
C8–C9	1.3588(19)	1.368
C9–C4	1.5442(19)	1.538
C1–Ge–C2	127.7(1)	129.1
C2–Ge–B	102.2(1)	102.6
Ge–B–P	96.2(1)	96.1

The summary of the natural population analysis results with natural charges of 1.36 for Ge, –0.33 for B, 1.32 for P, –0.19 for Cl.

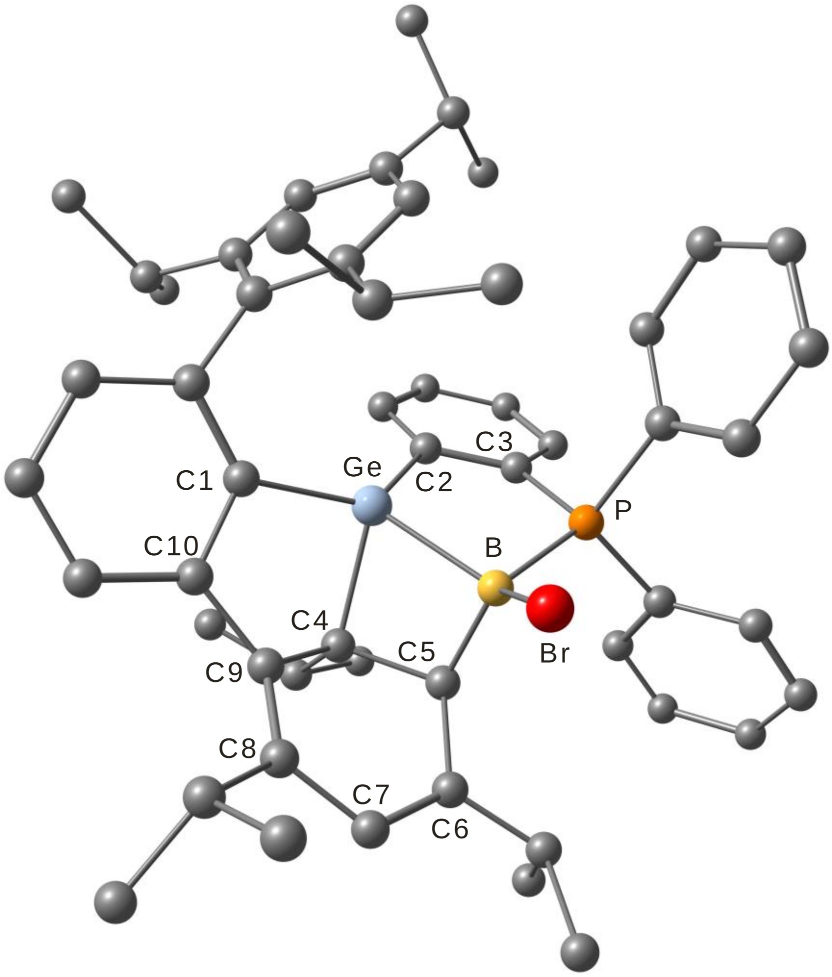

Figure S34. Optimized molecular structure **7**.

Table S7. Selected distances and angles of **7**, measured (molecular structure in the solid state) and calculated values

distance/angle	measured Å / °	calculated Å / °
Ge–B	2.111(5)	2.113
Ge–C1	1.939(4)	1.933
Ge–C2	1.961(4)	1.958
B–P	1.950(5)	1.942
C2–C3	1.402(6)	1.412
B–Br	2.005(5)	2.005
C3–P	1.826(4)	1.828
B–C5	1.662(7)	1.662
C4–C5	1.547(6)	1.552
C5–C6	1.499(6)	1.493
C6–C7	1.337(6)	1.352
C7–C8	1.474(6)	1.464
C8–C9	1.340(6)	1.368
C9–C4	1.549(6)	1.539

C1–Ge–C2	129.6(2)	129.3
C2–Ge–B	101.0(2)	102.2
Ge–B–P	97.5(2)	96.6

The summary of the natural population analysis results with natural charges of 1.37 for Ge, –0.39 for B, 1.32 for P, –0.13 for Br.

Compound **4** Gaussian optimized geometry
121

BGeC54PH63Cl

Ge	-0.034734000	-0.539404000	-0.083977000
Cl	-1.749629000	1.054350000	-2.648885000
P	-0.542449000	2.357437000	-0.054338000
C	0.943191000	-5.177931000	0.144812000
H	1.161961000	-6.246070000	0.201692000
C	-0.377396000	-4.733013000	0.238007000
H	-1.195021000	-5.445717000	0.363402000
C	2.754956000	-1.056228000	-1.477603000
C	1.620788000	0.785225000	3.666662000
H	2.106341000	0.376544000	4.555710000
C	1.988739000	-4.267929000	-0.038419000
H	3.010195000	-4.634948000	-0.138966000
C	-2.206144000	-3.519369000	3.340246000
H	-3.284013000	-3.513385000	3.565412000
H	-1.655690000	-3.622664000	4.288318000
H	-1.994219000	-4.402689000	2.721383000
C	2.029480000	-1.566869000	-2.717734000
H	1.106997000	-2.067975000	-2.386509000
C	0.728996000	2.680735000	2.456406000
H	0.528548000	3.751967000	2.385360000
C	-2.537172000	-2.121532000	1.282434000
C	0.344095000	1.817152000	1.433273000
C	4.369031000	-0.284890000	0.673273000
H	4.995614000	0.019127000	1.513312000
C	1.372523000	2.156983000	3.588383000
H	1.671247000	2.819572000	4.401898000
C	0.615292000	0.432712000	1.483602000
C	-0.663378000	-3.364459000	0.142496000
C	-1.803475000	-2.223473000	2.612326000
H	-0.727232000	-2.299780000	2.386461000
C	1.255909000	-0.071329000	2.618984000
H	1.464421000	-1.140056000	2.691085000
C	-1.996586000	-1.002541000	3.518813000
H	-1.773111000	-0.071492000	2.979626000
H	-1.319851000	-1.062617000	4.383506000
H	-3.024677000	-0.941730000	3.908177000
C	1.735679000	-2.890730000	-0.110115000
C	2.781746000	-1.838223000	-0.292719000
C	3.916904000	-2.438763000	1.923449000
H	2.985530000	-3.001965000	2.101417000
C	3.494117000	0.130885000	-1.518780000
H	3.452930000	0.756015000	-2.412332000
C	0.516033000	3.619447000	-0.837948000
C	-2.250801000	-3.454165000	-2.326811000
H	-1.375819000	-4.054534000	-2.037187000
C	-2.043425000	-2.793632000	0.137831000
C	4.857475000	1.957836000	-0.412991000
H	4.682058000	2.397399000	-1.410293000
C	3.672807000	-1.495055000	0.754039000
C	4.245057000	0.570662000	-0.426151000
C	-2.738823000	4.007393000	-0.470456000
H	-2.317386000	4.161522000	-1.465367000
C	1.414268000	3.180751000	-1.820273000
H	1.398428000	2.128966000	-2.113127000
C	5.017161000	-3.450958000	1.538769000
H	4.787635000	-3.973967000	0.601463000
H	5.153707000	-4.201930000	2.332381000
H	5.973905000	-2.924987000	1.394971000
C	-2.759440000	-2.748810000	-1.077754000
C	2.899502000	-2.640457000	-3.401391000
H	3.856025000	-2.207598000	-3.733490000
H	2.382696000	-3.052440000	-4.281706000
H	3.121561000	-3.468397000	-2.713196000
C	0.395396000	-2.450281000	-0.016393000
C	-1.782922000	-2.441688000	-3.383178000
H	-2.616130000	-1.810243000	-3.724829000
H	-1.360927000	-2.960287000	-4.258003000
H	-1.018308000	-1.765804000	-2.976425000
C	-2.602240000	3.012790000	1.742048000
H	-2.074984000	2.392528000	2.468163000
C	-2.043266000	3.230334000	0.472153000
C	0.519680000	4.961884000	-0.431564000

H	-0.194006000	5.306704000	0.319201000
C	1.627579000	-0.478396000	-3.714716000
H	1.008713000	0.293242000	-3.235907000
H	1.034061000	-0.918361000	-4.529140000
H	2.504852000	0.003321000	-4.174087000
C	2.319996000	4.078289000	-2.386811000
H	3.016437000	3.735224000	-3.154014000
C	4.319343000	-1.748096000	3.233465000
H	5.335113000	-1.328899000	3.169868000
H	4.322983000	-2.478195000	4.056439000
H	3.635998000	-0.931730000	3.496062000
C	-3.300491000	-4.417482000	-2.902263000
H	-3.620689000	-5.151927000	-2.148668000
H	-2.889318000	-4.963302000	-3.765227000
H	-4.195470000	-3.876583000	-3.245709000
C	6.373388000	1.938004000	-0.164976000
H	6.607832000	1.531143000	0.830676000
H	6.787391000	2.956873000	-0.212014000
H	6.891260000	1.318616000	-0.911850000
C	-3.714233000	-1.376880000	1.171477000
H	-4.080430000	-0.835149000	2.043359000
C	-3.831631000	3.585420000	2.070173000
H	-4.258189000	3.416601000	3.060558000
C	1.431385000	5.854920000	-0.997806000
H	1.438280000	6.898931000	-0.679153000
C	2.332485000	5.413721000	-1.973342000
H	3.041426000	6.115717000	-2.416237000
C	-4.403720000	-1.263472000	-0.040761000
C	-3.925617000	-1.978325000	-1.141114000
H	-4.461434000	-1.898316000	-2.091264000
C	-4.517172000	4.367046000	1.134024000
H	-5.480786000	4.809281000	1.392354000
C	-3.970926000	4.571530000	-0.136457000
H	-4.508721000	5.169406000	-0.874330000
C	-5.054061000	1.074919000	-0.600986000
H	-5.884657000	1.773586000	-0.785907000
H	-4.425373000	1.025707000	-1.500561000
H	-4.434131000	1.487085000	0.208418000
C	-5.580342000	-0.318729000	-0.208738000
H	-6.184751000	-0.702606000	-1.049682000
C	-6.487851000	-0.233609000	1.024748000
H	-5.963607000	0.227686000	1.875916000
H	-6.838438000	-1.227622000	1.339400000
H	-7.367714000	0.390955000	0.808763000
B	-0.876495000	0.840523000	-1.101242000
C	4.131214000	2.838116000	0.620299000
H	4.268077000	2.438852000	1.636954000
H	3.051762000	2.868692000	0.420999000
H	4.516844000	3.868885000	0.598456000

Compound **5** Gaussian optimized geometry
121

BGeC54PH63Br

C	0.147720000	1.872161000	1.834402000
C	0.627408000	0.543415000	1.823919000
C	1.225959000	0.047952000	2.986225000
H	1.587328000	-0.981676000	3.012798000
C	1.347987000	0.860317000	4.122699000
H	1.815290000	0.459008000	5.024782000
C	0.870234000	2.172495000	4.117135000
H	0.959901000	2.795908000	5.007908000
C	0.254127000	2.682170000	2.962858000
H	-0.149367000	3.697250000	2.956611000
C	-2.357230000	2.961212000	0.850347000
C	-3.375449000	2.003254000	0.964825000
H	-3.168385000	0.967688000	0.682248000
C	-4.633595000	2.382322000	1.432814000
H	-5.425044000	1.635706000	1.518618000
C	-4.882248000	3.714567000	1.778370000
H	-5.869902000	4.010464000	2.137049000
C	-3.869243000	4.671456000	1.656253000
H	-4.064746000	5.712509000	1.920002000
C	-2.605680000	4.298131000	1.192587000
H	-1.816295000	5.044246000	1.084473000
C	0.119839000	3.819546000	-0.388204000
C	1.417062000	4.179743000	0.007423000
H	1.903983000	3.640662000	0.821308000
C	2.080446000	5.220497000	-0.644508000
H	3.088097000	5.497978000	-0.330824000
C	1.459450000	5.901144000	-1.696613000
H	1.981421000	6.713636000	-2.205258000
C	0.169981000	5.537707000	-2.099076000
H	-0.314390000	6.061770000	-2.924972000
C	-0.501860000	4.501435000	-1.450147000
H	-1.504004000	4.211590000	-1.770739000
C	0.719484000	-2.292610000	0.260237000
C	2.069776000	-2.693301000	0.185604000

C	2.371333000	-4.055970000	0.328522000
H	3.406265000	-4.388063000	0.239842000
C	1.359074000	-4.990296000	0.561595000
H	1.613400000	-6.045335000	0.680497000
C	0.022281000	-4.587503000	0.604799000
H	-0.773299000	-5.322456000	0.741513000
C	-0.311441000	-3.238015000	0.427324000
C	-1.722587000	-2.769604000	0.280670000
C	-2.312696000	-2.839864000	-1.002443000
C	-4.266203000	-1.640907000	-0.157138000
C	-3.671571000	-1.603338000	1.107760000
H	-4.207911000	-1.108086000	1.920607000
C	-2.403302000	-2.144078000	1.348127000
C	-3.575445000	-2.272143000	-1.195376000
H	-4.019716000	-2.300664000	-2.192686000
C	3.107338000	-1.660847000	-0.109659000
C	4.072004000	-1.288168000	0.854854000
C	4.943016000	-0.229367000	0.565833000
H	5.677404000	0.075532000	1.315043000
C	4.892021000	0.467446000	-0.641536000
C	3.962840000	0.054005000	-1.601679000
H	3.921193000	0.571358000	-2.560483000
C	3.075290000	-0.999602000	-1.369783000
C	5.793749000	1.664834000	-0.880240000
H	6.541325000	1.671504000	-0.067867000
C	6.549128000	1.581640000	-2.215274000
H	7.124984000	0.647666000	-2.288208000
H	5.855600000	1.617281000	-3.069068000
H	7.245895000	2.427184000	-2.319687000
C	4.980513000	2.968242000	-0.782255000
H	4.480574000	3.043676000	0.194245000
H	5.629464000	3.848646000	-0.911570000
H	4.200045000	3.001053000	-1.557370000
C	5.319425000	-3.093769000	2.084302000
H	5.132084000	-3.803245000	1.267374000
H	6.293397000	-2.617365000	1.892375000
H	5.395484000	-3.661764000	3.024457000
C	4.215068000	-2.022999000	2.180567000
H	3.262182000	-2.539766000	2.379085000
C	4.511641000	-1.089892000	3.366003000
H	4.414016000	-1.639920000	4.314339000
H	5.540268000	-0.700546000	3.322929000
H	3.827737000	-0.231794000	3.388404000
C	2.178554000	-1.489311000	-2.503700000
H	1.255884000	-1.892794000	-2.062518000
C	2.865499000	-2.658415000	-3.234683000
H	3.084854000	-3.484597000	-2.543017000
H	2.217554000	-3.043846000	-4.036890000
H	3.814565000	-2.329422000	-3.685717000
C	1.762216000	-0.387326000	-3.482774000
H	1.318425000	0.464958000	-2.948942000
H	2.611388000	-0.024687000	-4.082632000
H	1.009319000	-0.774863000	-4.183855000
C	-1.597478000	-3.513473000	-2.165760000
H	-0.546710000	-3.657457000	-1.872685000
C	-1.596059000	-2.663078000	-3.442962000
H	-0.991101000	-3.152129000	-4.221680000
H	-2.610509000	-2.528782000	-3.848522000
H	-1.183034000	-1.663151000	-3.252976000
C	-2.196666000	-4.906926000	-2.424919000
H	-1.657712000	-5.418686000	-3.237162000
H	-2.142920000	-5.535919000	-1.524400000
H	-3.256145000	-4.826220000	-2.714854000
C	-1.768002000	-2.059221000	2.727629000
H	-0.676642000	-2.032002000	2.577180000
C	-2.083766000	-3.327339000	3.541199000
H	-1.597293000	-3.288826000	4.528287000
H	-3.169630000	-3.424949000	3.696160000
H	-1.733042000	-4.229531000	3.020081000
C	-2.151260000	-0.794360000	3.504096000
H	-1.544615000	-0.715557000	4.417637000
H	-1.975371000	0.111123000	2.907109000
H	-3.208562000	-0.805970000	3.810170000
C	-5.562438000	0.068136000	-1.498413000
H	-4.789282000	0.820791000	-1.291195000
H	-5.324124000	-0.386328000	-2.471975000
H	-6.532013000	0.580399000	-1.595851000
C	-6.695388000	-2.056816000	-0.700304000
H	-6.760367000	-2.801824000	0.105999000
H	-7.684059000	-1.587916000	-0.822135000
H	-6.458518000	-2.591400000	-1.633116000
C	-5.621673000	-0.999167000	-0.393623000
H	-5.905545000	-0.498230000	0.549172000
B	-0.805708000	0.869901000	-0.795650000
P	-0.717478000	2.374750000	0.317174000
Ge	0.247592000	-0.400876000	0.153565000
Br	-1.733040000	1.049334000	-2.496867000

Compound **6** Gaussian optimized geometry
121

BGeC54PH63Cl

Cl	-2.005947000	0.027776000	2.329332000
Ge	0.098861000	0.631465000	-0.413941000
P	-1.216562000	-2.054783000	0.071936000
C	-1.490486000	1.596084000	-1.186171000
C	-1.637938000	1.964359000	-2.674832000
H	-2.554634000	2.580790000	-2.768602000
C	-0.456173000	2.804016000	-3.177126000
H	-0.592790000	3.073227000	-4.235719000
H	0.485226000	2.236086000	-3.091351000
H	-0.334594000	3.730944000	-2.601821000
C	-1.806525000	0.732613000	-3.575173000
H	-1.838717000	1.032350000	-4.633759000
H	-2.736195000	0.184872000	-3.364789000
H	-0.961942000	0.036226000	-3.448591000
C	-1.394829000	2.832242000	-0.275361000
C	-0.011730000	3.320220000	-0.035341000
C	0.419426000	4.641606000	0.189758000
H	-0.280601000	5.473179000	0.097405000
C	1.760112000	4.898440000	0.497863000
H	2.080648000	5.929166000	0.663589000
C	2.698721000	3.863950000	0.605287000
H	3.731490000	4.090198000	0.876198000
C	2.307008000	2.538969000	0.358508000
C	0.974312000	2.302933000	0.008830000
C	3.174724000	1.325601000	0.445017000
C	4.126430000	1.039066000	-0.559886000
C	4.533010000	2.102155000	-1.571790000
H	3.717572000	2.840876000	-1.617707000
C	5.795678000	2.829768000	-1.069539000
H	6.072757000	3.651431000	-1.747999000
H	6.643936000	2.129573000	-1.011705000
H	5.642343000	3.247002000	-0.064645000
C	4.764468000	1.573653000	-2.993922000
H	4.999845000	2.408192000	-3.671135000
H	3.877182000	1.062079000	-3.392758000
H	5.608819000	0.869196000	-3.038712000
C	4.728025000	-0.225993000	-0.583328000
H	5.447067000	-0.456926000	-1.372531000
C	4.413536000	-1.208973000	0.358896000
C	5.007838000	-2.603818000	0.278962000
H	4.665417000	-3.146108000	1.177806000
C	4.481289000	-3.357004000	-0.953860000
H	4.861836000	-4.390056000	-0.978415000
H	4.799573000	-2.855682000	-1.880863000
H	3.382736000	-3.389308000	-0.963583000
C	6.544520000	-2.584544000	0.300966000
H	6.948426000	-3.608609000	0.304741000
H	6.921026000	-2.059544000	1.190660000
H	6.945837000	-2.071445000	-0.586509000
C	3.516739000	-0.883529000	1.381028000
H	3.288212000	-1.639353000	2.134534000
C	2.897011000	0.368314000	1.456163000
C	2.002623000	0.723908000	2.639963000
H	1.124601000	1.262853000	2.250829000
C	1.481894000	-0.484492000	3.419707000
H	0.753574000	-0.154255000	4.173023000
H	2.291482000	-1.014531000	3.945968000
H	0.971172000	-1.199240000	2.763342000
C	2.751419000	1.689687000	3.579302000
H	2.109636000	1.974683000	4.426932000
H	3.050896000	2.607141000	3.054389000
H	3.659097000	1.210248000	3.978723000
C	-2.503663000	3.219082000	0.426059000
C	-3.751632000	2.463650000	0.296894000
H	-4.659861000	2.924395000	0.690236000
C	-3.782737000	1.185098000	-0.142170000
C	-5.005090000	0.298449000	-0.157783000
H	-4.651255000	-0.706544000	0.145774000
C	-5.564358000	0.168974000	-1.588584000
H	-6.369575000	-0.580740000	-1.628064000
H	-4.784149000	-0.138107000	-2.299414000
H	-5.969585000	1.134918000	-1.927450000
C	-6.105429000	0.726379000	0.817328000
H	-6.925444000	-0.007565000	0.818494000
H	-6.535978000	1.698884000	0.531624000
H	-5.714004000	0.812785000	1.840838000
C	-2.500693000	0.573001000	-0.600983000
H	-2.739264000	-0.171211000	-1.378718000
C	-2.503292000	4.336160000	1.456670000
H	-1.455603000	4.601543000	1.659533000
C	-3.115523000	3.885528000	2.794442000
H	-2.974800000	4.667149000	3.557067000
H	-2.640378000	2.959729000	3.147954000

H	-4.195639000	3.694603000	2.708897000
C	-3.211495000	5.588421000	0.912047000
H	-3.195255000	6.402020000	1.654129000
H	-4.264905000	5.372870000	0.672783000
H	-2.729596000	5.947499000	-0.009666000
C	0.755639000	-0.868554000	-1.488703000
C	-0.001122000	-2.044016000	-1.292259000
C	-2.652451000	-2.976926000	-0.553284000
C	-3.082320000	-2.723237000	-1.869468000
H	-2.465464000	-2.119801000	-2.537144000
C	-4.289121000	-3.251083000	-2.328121000
H	-4.613763000	-3.044176000	-3.349187000
C	-5.079125000	-4.036675000	-1.483916000
H	-6.025378000	-4.444820000	-1.842736000
C	-4.648964000	-4.304477000	-0.180943000
H	-5.255146000	-4.928050000	0.478566000
C	-3.443543000	-3.777088000	0.285931000
H	-3.115850000	-3.994785000	1.302729000
C	-0.430517000	-3.001932000	1.407522000
C	-1.076557000	-3.132561000	2.650539000
H	-2.054745000	-2.680220000	2.809731000
C	-0.437249000	-3.788238000	3.703131000
H	-0.941792000	-3.877111000	4.666557000
C	0.850656000	-4.305312000	3.534092000
H	1.351208000	-4.806448000	4.364418000
C	1.501431000	-4.163331000	2.306344000
H	2.512684000	-4.550598000	2.170455000
C	0.867445000	-3.512438000	1.247247000
H	1.392540000	-3.380254000	0.301963000
C	0.223113000	-3.184986000	-2.077977000
H	-0.372958000	-4.086467000	-1.919917000
C	1.215906000	-3.162265000	-3.059054000
H	1.397639000	-4.048846000	-3.668699000
C	1.981463000	-2.005423000	-3.251792000
H	2.763678000	-1.992665000	-4.013553000
C	1.749228000	-0.869340000	-2.475343000
H	2.342826000	0.029320000	-2.637483000
B	-1.590350000	-0.201282000	0.556207000

Compound **7** Gaussian optimized geometry
121

BGeC54PH63Br

Br	-2.065737000	0.033716000	2.315451000
Ge	0.191161000	0.626478000	-0.489734000
P	-1.124087000	-2.068219000	-0.056816000
C	-1.373242000	1.581077000	-1.322585000
C	-1.467739000	1.948231000	-2.816235000
H	-2.380692000	2.564337000	-2.942116000
C	-0.269735000	2.788863000	-3.276362000
H	-0.373628000	3.066062000	-4.336594000
H	0.667650000	2.218490000	-3.166743000
H	-0.164905000	3.711226000	-2.690546000
C	-1.605522000	0.715915000	-3.721390000
H	-1.593554000	1.014412000	-4.780739000
H	-2.545813000	0.173508000	-3.547763000
H	-0.770636000	0.015413000	-3.559763000
C	-1.321854000	2.819640000	-0.411312000
C	0.047517000	3.314014000	-0.113701000
C	0.463610000	4.638334000	0.122703000
H	-0.235477000	5.466562000	-0.000321000
C	1.790368000	4.903174000	0.479886000
H	2.098890000	5.936133000	0.654221000
C	2.730667000	3.874709000	0.622029000
H	3.752527000	4.107699000	0.926256000
C	2.355913000	2.546871000	0.364147000
C	1.036806000	2.302294000	-0.027851000
C	3.233512000	1.341829000	0.467307000
C	4.214029000	1.074284000	-0.515273000
C	4.632482000	2.147873000	-1.511355000
H	3.809649000	2.876948000	-1.574321000
C	5.873423000	2.887664000	-0.974199000
H	6.156853000	3.716984000	-1.640624000
H	6.728648000	2.197598000	-0.899556000
H	5.690210000	3.296486000	0.029108000
C	4.905098000	1.630583000	-2.930378000
H	5.148796000	2.471907000	-3.596148000
H	4.033248000	1.113167000	-3.354945000
H	5.757375000	0.934891000	-2.958501000
C	4.831482000	-0.183222000	-0.532241000
H	5.572012000	-0.399977000	-1.305401000
C	4.505964000	-1.176496000	0.395141000
C	5.116996000	-2.564257000	0.318221000
H	4.766932000	-3.114778000	1.209052000
C	4.616258000	-3.315883000	-0.926351000
H	5.007459000	-4.344919000	-0.951439000
H	4.942726000	-2.805421000	-1.845458000

H	3.518326000	-3.359208000	-0.952306000
C	6.652780000	-2.529358000	0.362880000
H	7.067077000	-3.549221000	0.370821000
H	7.010923000	-2.002364000	1.258956000
H	7.062171000	-2.010804000	-0.517705000
C	3.580199000	-0.869084000	1.396811000
H	3.342814000	-1.632592000	2.139635000
C	2.941980000	0.373853000	1.464371000
C	2.006691000	0.706333000	2.623181000
H	1.121281000	1.213031000	2.207559000
C	1.506651000	-0.513274000	3.399175000
H	0.753313000	-0.199321000	4.134614000
H	2.321563000	-1.014181000	3.945642000
H	1.030361000	-1.248609000	2.739451000
C	2.695890000	1.701200000	3.577639000
H	2.021064000	1.965585000	4.405886000
H	2.977578000	2.627265000	3.058359000
H	3.607963000	1.254378000	4.004038000
C	-2.463470000	3.207585000	0.234746000
C	-3.698200000	2.440651000	0.059890000
H	-4.624531000	2.894724000	0.416892000
C	-3.702341000	1.158194000	-0.368970000
C	-4.914885000	0.259445000	-0.415846000
H	-4.558854000	-0.740608000	-0.099143000
C	-5.433682000	0.120755000	-1.860833000
H	-6.227446000	-0.639709000	-1.920590000
H	-4.630481000	-0.176859000	-2.549898000
H	-5.842446000	1.080717000	-2.212236000
C	-6.043885000	0.678820000	0.529636000
H	-6.854705000	-0.065098000	0.514668000
H	-6.478940000	1.644683000	0.228704000
H	-5.677274000	0.774136000	1.561514000
C	-2.400146000	0.552362000	-0.778348000
H	-2.607858000	-0.190016000	-1.566796000
C	-2.517039000	4.339590000	1.247710000
H	-1.481294000	4.615069000	1.493114000
C	-3.186135000	3.908823000	2.564500000
H	-3.090621000	4.708317000	3.315498000
H	-2.716491000	2.997356000	2.959652000
H	-4.258540000	3.701497000	2.433391000
C	-3.207536000	5.577810000	0.650754000
H	-3.229629000	6.403144000	1.379566000
H	-4.247924000	5.351311000	0.368458000
H	-2.686788000	5.924944000	-0.254185000
C	0.889349000	-0.865662000	-1.548006000
C	0.125405000	-2.042076000	-1.390761000
C	-2.540306000	-2.993532000	-0.720356000
C	-2.931415000	-2.745210000	-2.049478000
H	-2.294365000	-2.145534000	-2.701328000
C	-4.125866000	-3.272545000	-2.540006000
H	-4.420670000	-3.069987000	-3.570927000
C	-4.942050000	-4.051241000	-1.714674000
H	-5.878994000	-4.458293000	-2.098318000
C	-4.550403000	-4.313487000	-0.398569000
H	-5.177499000	-4.931170000	0.246721000
C	-3.357307000	-3.787162000	0.099916000
H	-3.060631000	-4.000340000	1.126819000
C	-0.352737000	-3.021683000	1.284479000
C	-1.026752000	-3.204212000	2.505954000
H	-2.020844000	-2.783432000	2.652436000
C	-0.397197000	-3.870837000	3.557550000
H	-0.924483000	-3.999003000	4.504198000
C	0.907934000	-4.349618000	3.409620000
H	1.399810000	-4.860247000	4.239342000
C	1.586173000	-4.156794000	2.203990000
H	2.610870000	-4.512721000	2.084350000
C	0.962758000	-3.493157000	1.146534000
H	1.510027000	-3.319212000	0.221060000
C	0.373031000	-3.172901000	-2.183962000
H	-0.226760000	-4.076489000	-2.055272000
C	1.395085000	-3.137862000	-3.134283000
H	1.594214000	-4.016193000	-3.750402000
C	2.168306000	-1.980168000	-3.286913000
H	2.973817000	-1.958595000	-4.023795000
C	1.914172000	-0.854581000	-2.502156000
H	2.514919000	0.044545000	-2.632062000
B	-1.525968000	-0.224111000	0.402130000

NBO and NRT analyses were performed on model complexes with reduced organic complexity directly derived from the X-ray structures of **4** and **5**. NBO and NRT analyses were performed with NBO7 programme[9] on a single point calculation using PBE0[10] functional and def-SVP

basis set[5c, 5d] for all elements on a structure optimised using BP86-D3BJ functional and def2svp basis set.[5b, 6, 11] The general geometric characteristics of the central heterocycles are virtually not affected by the computational ligand reduction.

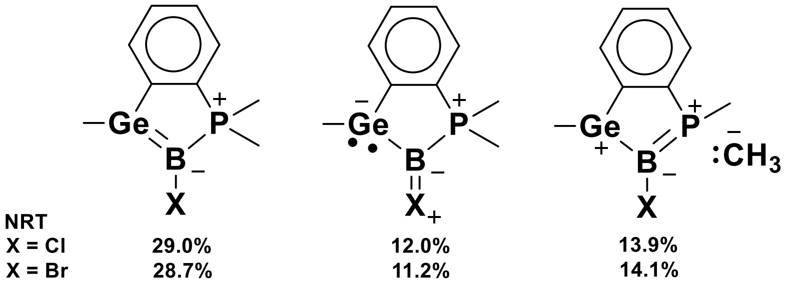

Figure S35. Results of NRT analyses.

XYZ coordinates (**4***)

Ge	7.831158000	12.489016000	14.356643000
Cl	11.239320000	12.333888000	14.939700000
P	9.140503000	9.976144000	15.209326000
C	4.597063000	9.608462000	14.576022000
H	3.508675000	9.552024000	14.416485000
C	6.693507000	8.539908000	15.186010000
H	7.250332000	7.643807000	15.504774000
C	7.354561000	9.757334000	14.979464000
C	5.299461000	8.463102000	14.982594000
H	4.770162000	7.511914000	15.142180000
C	6.658613000	10.929808000	14.566998000
C	5.267077000	10.830976000	14.369376000
H	4.691882000	11.714779000	14.050354000
C	9.987484000	8.754024000	14.151626000
C	6.957261000	14.146164000	13.778795000
C	9.544625000	9.460038000	16.912525000
B	9.540852000	11.773508000	14.811854000
H	7.709732000	14.951715000	13.688971000
H	6.467571000	14.002483000	12.795794000
H	6.189072000	14.454921000	14.514642000
H	10.622245000	9.646123000	17.089744000
H	8.956275000	10.082293000	17.612569000
H	9.321282000	8.388720000	17.082201000
H	11.080299000	8.915018000	14.234818000
H	9.743167000	7.714916000	14.446097000
H	9.680042000	8.929636000	13.103866000

XYZ coordinates (**5***)

C	1.142292000	0.644239000	2.852833000
C	0.251284000	0.471999000	3.950860000
C	-1.009287000	1.096770000	3.878205000
H	-1.726545000	0.988299000	4.707536000
C	-1.366907000	1.863588000	2.751326000
H	-2.357523000	2.343783000	2.713014000
C	-0.476289000	2.022184000	1.677862000
H	-0.761713000	2.621581000	0.800511000
C	0.791555000	1.405678000	1.730715000
H	1.494414000	1.527830000	0.890639000
B	2.726306000	-1.071492000	4.691873000
P	2.743004000	-0.192197000	3.033620000
Ge	1.002631000	-0.643430000	5.380464000
Br	4.305694000	-2.089521000	5.226635000
C	-0.141880000	-0.935924000	6.943311000
H	-1.099736000	-1.400361000	6.637130000
H	0.370062000	-1.607987000	7.657154000
H	-0.360379000	0.024963000	7.449238000
C	2.964583000	-1.284472000	1.590196000
H	3.907935000	-1.849589000	1.721570000

H	2.122465000	-2.000325000	1.562687000
H	3.004075000	-0.713428000	0.642811000
C	4.047020000	1.070461000	2.856069000
H	3.891639000	1.842379000	3.632528000
H	5.028153000	0.585264000	3.026693000
H	4.034093000	1.538542000	1.852733000

TDDFT calculations

TDDFT calculations were performed in ORCA4.1.[12] A TDDFT single-point calculation with CAM-B3LYP functional[13] and RIJCOSX approximation[14] with def2-SVP basis set on all atoms was performed on structures obtained BP86-D3BJ optimisations with def2-TZVP basis sets. [5b, 5d, 6, 11] Difference Density plots were obtained using the orca_plot programme that is included in the ORCA4.1 distribution. Graphical depictions were produced with ChemCraft.[15]

The simulated TDDFT spectra of **4** and **6** are in good agreement with the experimental spectra of **4** and **6.**

Table S8. Summary of TDDFT results for 4 (CAM-B3LYP/def2SVP // BP86-D3BJ/def2TZVP)

excitation	energy (cm^{-1})	wavelength (nm)	F_{osc}	orbital contributions (HOMO: 227a)
1	19863.2	503.4	0.052744534	227a -> 228a : 0.931371 (c= -0.96507559)<br>227a -> 229a : 0.016008 (c= 0.12652098)<br>227a -> 230a : 0.010752 (c= -0.10369045)
2	24648.4	405.7	0.01683572	227a -> 228a : 0.020662 (c= -0.14374286)<br>227a -> 229a : 0.112272 (c= -0.33506958)<br>227a -> 230a : 0.698987 (c= 0.83605422)<br>227a -> 231a : 0.037588 (c= 0.19387732)<br>227a -> 232a : 0.018048 (c= 0.13434117)<br>227a -> 233a : 0.031730 (c= 0.17812814)<br>227a -> 240a : 0.022512 (c= -0.15004137)<br>227a -> 242a : 0.013429 (c= -0.11588308)
3	25632.4	390.1	0.098013396	227a -> 229a : 0.843685 (c= 0.91852302)<br>227a -> 230a : 0.103121 (c= 0.32112514)
4	28070.3	356.2	0.003391853	227a -> 230a : 0.096680 (c= 0.31093454)<br>227a -> 231a : 0.681839 (c= -0.82573563)<br>227a -> 232a : 0.053179 (c= -0.23060522)<br>227a -> 233a : 0.129020 (c= -0.35919367)<br>227a -> 234a : 0.012243 (c= -0.11065008)
5	28853.2	346.6	0.036632219	227a -> 232a : 0.587759 (c= -0.76665435)<br>227a -> 233a : 0.322272 (c= 0.56769049)<br>227a -> 234a : 0.033557 (c= 0.18318649)<br>227a -> 235a : 0.016281 (c= 0.12759514)
6	29281.8	341.5	0.031300013	227a -> 230a : 0.018246 (c= 0.13507840)<br>227a -> 231a : 0.236145 (c= 0.48594704)<br>227a -> 232a : 0.312308 (c= -0.55884519)<br>227a -> 233a : 0.362110 (c= -0.60175583)<br>227a -> 234a : 0.032618 (c= -0.18060548)<br>227a -> 235a : 0.015243 (c= -0.12346431)
7	31114.0	321.4	0.045998744	227a -> 233a : 0.070566 (c= -0.26564192)<br>227a -> 234a : 0.871832 (c= 0.93371936)<br>227a -> 235a : 0.021507 (c= -0.14665434)
8	32615.3	306.6	0.028920477	227a -> 233a : 0.037228 (c= -0.19294477)<br>227a -> 234a : 0.011704 (c= 0.10818641)<br>227a -> 235a : 0.908448 (c= 0.95312553)

9	34170.1	292.7	0.007066158	227a -> 236a : 0.895889 (c= -0.94651415)<br>227a -> 238a : 0.024411 (c= -0.15624102)<br>227a -> 239a : 0.018812 (c= -0.13715638)<br>227a -> 240a : 0.010454 (c= 0.10224497)
10	34970.9	286.0	0.016358208	227a -> 237a : 0.656886 (c= 0.81048483)<br>227a -> 238a : 0.259612 (c= 0.50952100)<br>227a -> 239a : 0.029950 (c= -0.17306118)

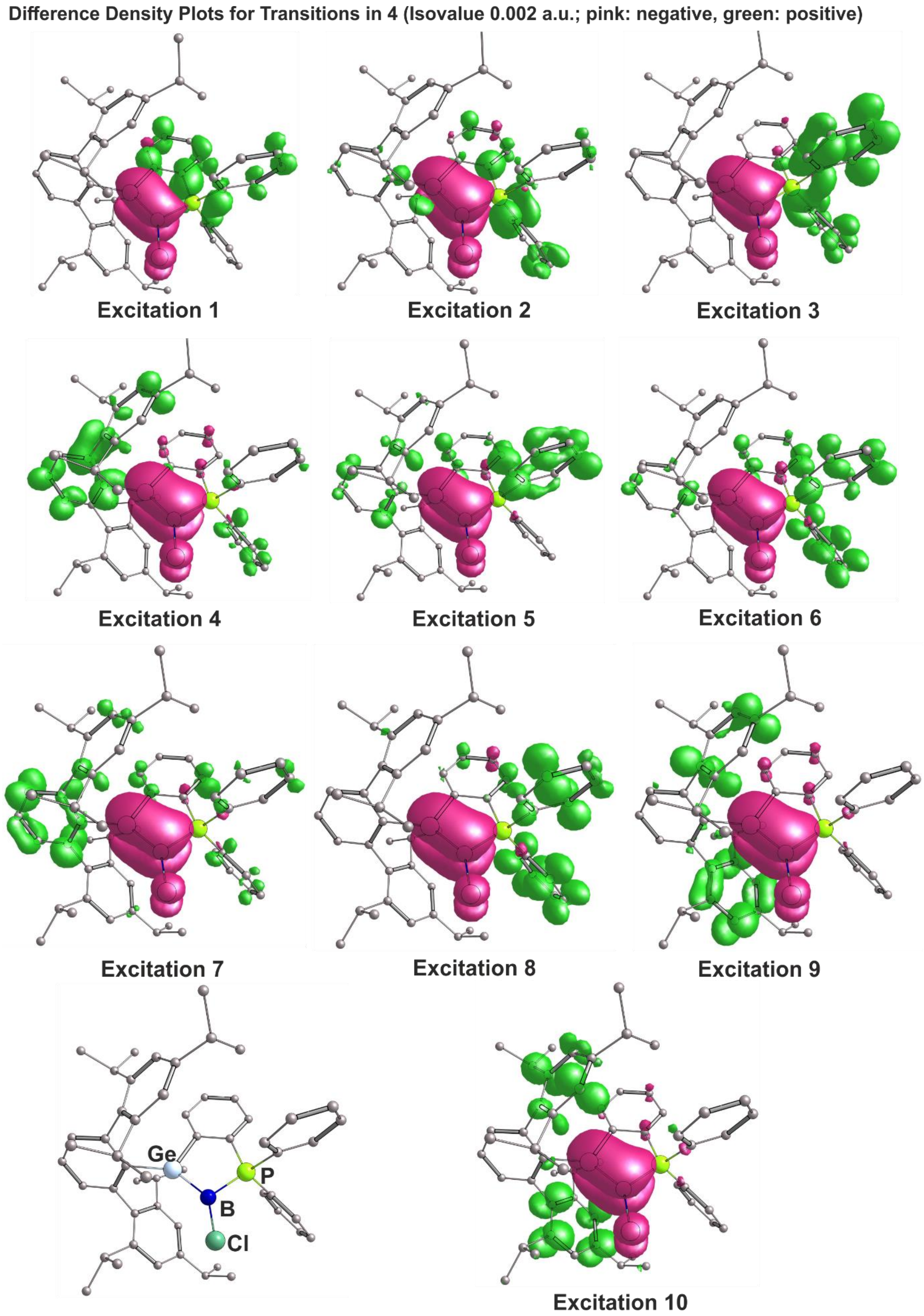

Figure S 36. Density plots for Transitions in **4**.

Table S9. Summary of TDDFT results for 6 (CAM-B3LYP/def2SVP // BP86-D3BJ/def2TZVP)

excitation	energy (cm^{-1})	wavelength (nm)	F_{osc}	orbital contributions (HOMO: 227a)
1	31211.5	320.4	0.190329279	226a -> 229a : 0.012824 (c= 0.11324401)<br>226a -> 230a : 0.013899 (c= -0.11789609)<br>227a -> 228a : 0.188329 (c= 0.43396851)<br>227a -> 229a : 0.175921 (c= 0.41942924)<br>227a -> 230a : 0.159997 (c= -0.39999677)<br>227a -> 231a : 0.045495 (c= -0.21329450)<br>227a -> 232a : 0.232766 (c= 0.48245836)<br>227a -> 233a : 0.059106 (c= -0.24311651)<br>227a -> 236a : 0.030827 (c= -0.17557657)
2	31379.9	318.7	0.005520074	226a -> 228a : 0.036155 (c= -0.19014503)<br>226a -> 229a : 0.273445 (c= 0.52292003)<br>226a -> 230a : 0.025235 (c= -0.15885436)<br>227a -> 228a : 0.093751 (c= -0.30618753)<br>227a -> 229a : 0.379522 (c= 0.61605332)<br>227a -> 232a : 0.086719 (c= -0.29448112)<br>227a -> 233a : 0.012922 (c= 0.11367633)<br>227a -> 234a : 0.017127 (c= 0.13087110)<br>227a -> 235a : 0.022783 (c= 0.15093987)
3	32466.5	308.0	0.016609380	226a -> 228a : 0.265928 (c= -0.51568181)<br>226a -> 232a : 0.011289 (c= -0.10625202)<br>227a -> 228a : 0.320404 (c= -0.56604243)<br>227a -> 229a : 0.017873 (c= -0.13368838)<br>227a -> 230a : 0.077521 (c= -0.27842643)<br>227a -> 232a : 0.111764 (c= 0.33431167)<br>227a -> 234a : 0.058261 (c= -0.24137336)<br>227a -> 235a : 0.056106 (c= -0.23686675)
4	34073.6	293.5	0.006719637	225a -> 229a : 0.016711 (c= 0.12927012)<br>226a -> 228a : 0.111616 (c= 0.33408997)<br>226a -> 229a : 0.227655 (c= 0.47713172)<br>226a -> 230a : 0.147817 (c= -0.38446987)<br>226a -> 231a : 0.016685 (c= -0.12916901)<br>226a -> 234a : 0.013540 (c= 0.11636078)<br>227a -> 229a : 0.158168 (c= -0.39770369)<br>227a -> 230a : 0.121283 (c= -0.34825730)<br>227a -> 233a : 0.023852 (c= 0.15444220)<br>227a -> 235a : 0.090195 (c= -0.30032542)<br>227a -> 237a : 0.010556 (c= -0.10274046)
5	35209.0	284.0	0.019329829	225a -> 228a : 0.032237 (c= 0.17954645)<br>225a -> 229a : 0.019530 (c= -0.13975122)<br>226a -> 228a : 0.314851 (c= 0.56111612)<br>226a -> 229a : 0.156181 (c= -0.39519745)<br>227a -> 228a : 0.216376 (c= -0.46516220)<br>227a -> 229a : 0.084207 (c= 0.29018518)<br>227a -> 230a : 0.111813 (c= -0.33438473)
6	35788.0	279.4	0.082291447	222a -> 235a : 0.011044 (c= 0.10508929)<br>225a -> 229a : 0.016610 (c= -0.12887873)<br>226a -> 228a : 0.053715 (c= -0.23176533)<br>226a -> 229a : 0.072121 (c= -0.26855348)<br>226a -> 231a : 0.024830 (c= 0.15757536)<br>226a -> 232a : 0.071816 (c= -0.26798443)<br>226a -> 233a : 0.022175 (c= 0.14891292)<br>227a -> 228a : 0.081227 (c= 0.28500266)<br>227a -> 229a : 0.017666 (c= 0.13291245)<br>227a -> 230a : 0.161521 (c= -0.40189727)

				227a -> 232a : 0.148748 (c= -0.38567865)<br>227a -> 233a : 0.106516 (c= 0.32636853)<br>227a -> 234a : 0.021891 (c= 0.14795731)<br>227a -> 235a : 0.097533 (c= -0.31230303)
7	36479.1	274.1	0.188887121	226a -> 228a : 0.076337 (c= -0.27629133)<br>226a -> 229a : 0.065548 (c= -0.25602283)<br>226a -> 230a : 0.191613 (c= -0.43773629)<br>226a -> 231a : 0.011704 (c= -0.10818348)<br>226a -> 232a : 0.077768 (c= 0.27886911)<br>226a -> 235a : 0.010240 (c= -0.10119274)<br>227a -> 229a : 0.051044 (c= -0.22592912)<br>227a -> 230a : 0.091760 (c= -0.30291976)<br>227a -> 231a : 0.020024 (c= -0.14150543)<br>227a -> 232a : 0.027494 (c= -0.16581366)<br>227a -> 234a : 0.062820 (c= 0.25063853)<br>227a -> 235a : 0.199121 (c= 0.44622956)<br>227a -> 237a : 0.013084 (c= 0.11438726)
8	36850.9	271.4	0.025403705	226a -> 230a : 0.114849 (c= 0.33889352)<br>226a -> 231a : 0.170720 (c= -0.41318264)<br>226a -> 234a : 0.014578 (c= 0.12073993)<br>227a -> 228a : 0.019571 (c= -0.13989564)<br>227a -> 231a : 0.566622 (c= -0.75274266)<br>227a -> 234a : 0.048427 (c= 0.22006058)
9	37799.7	264.6	0.120904129	225a -> 230a : 0.020194 (c= 0.14210675)<br>226a -> 228a : 0.012498 (c= 0.11179486)<br>226a -> 229a : 0.089998 (c= 0.29999721)<br>226a -> 230a : 0.206381 (c= 0.45429125)<br>226a -> 231a : 0.106253 (c= 0.32596455)<br>226a -> 232a : 0.047963 (c= -0.21900537)<br>226a -> 235a : 0.024870 (c= 0.15770383)<br>227a -> 229a : 0.064116 (c= -0.25321049)<br>227a -> 230a : 0.134724 (c= -0.36704702)<br>227a -> 232a : 0.013272 (c= -0.11520311)<br>227a -> 233a : 0.019671 (c= -0.14025328)<br>227a -> 234a : 0.018155 (c= 0.13474111)<br>227a -> 235a : 0.166241 (c= 0.40772675)
10	38823.4	257.6	0.008605512	224a -> 232a : 0.016900 (c= -0.12999894)<br>225a -> 229a : 0.012441 (c= 0.11153797)<br>225a -> 230a : 0.040186 (c= -0.20046435)<br>226a -> 228a : 0.013436 (c= 0.11591473)<br>226a -> 229a : 0.014845 (c= -0.12183971)<br>226a -> 230a : 0.081354 (c= -0.28522564)<br>226a -> 232a : 0.388534 (c= -0.62332470)<br>226a -> 233a : 0.065291 (c= 0.25552166)<br>226a -> 234a : 0.023772 (c= 0.15418181)<br>226a -> 236a : 0.019506 (c= 0.13966356)<br>227a -> 231a : 0.041313 (c= -0.20325536)<br>227a -> 232a : 0.012950 (c= -0.11379764)<br>227a -> 233a : 0.077047 (c= -0.27757398)<br>227a -> 234a : 0.014396 (c= -0.11998208)<br>227a -> 235a : 0.029153 (c= 0.17074369)<br>227a -> 238a : 0.013695 (c= 0.11702739)

Difference Density Plots for Transitions in 6 (Isovalue 0.002 a.u.; pink: negative, green: positive)

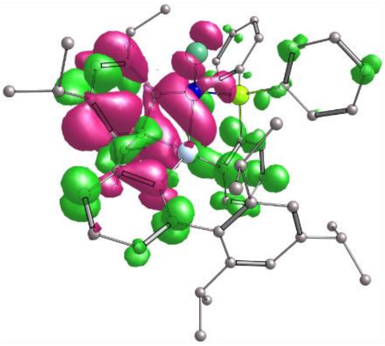

Excitation 1 (isovalue 0.001 a.u)

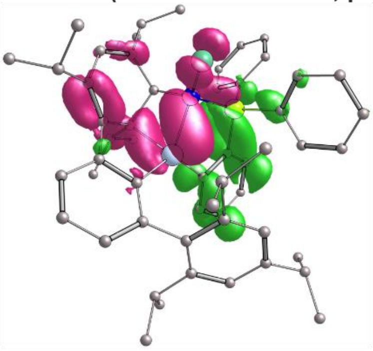

Excitation 2

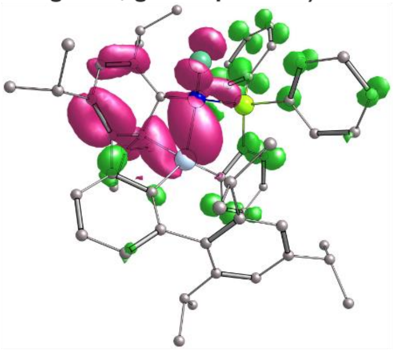

Excitation 3

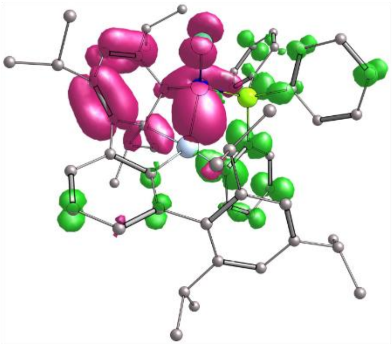

Excitation 4

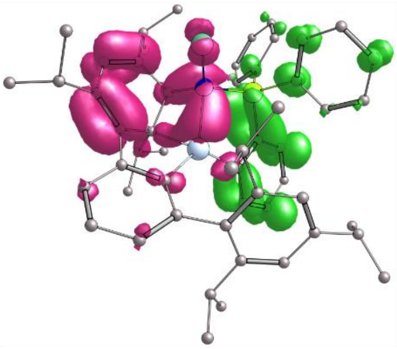

Excitation 5

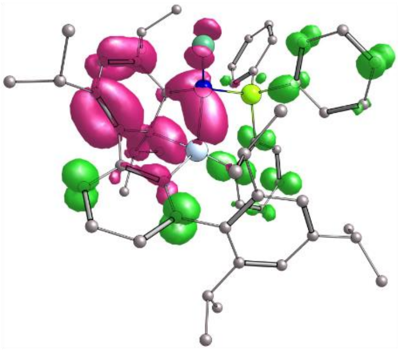

Excitation 6

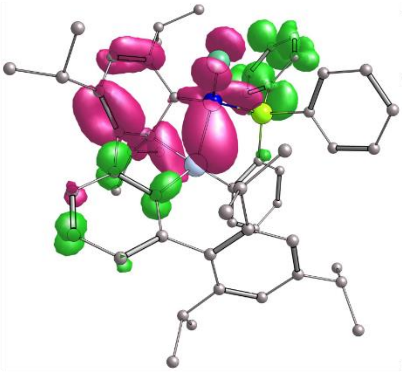

Excitation 7

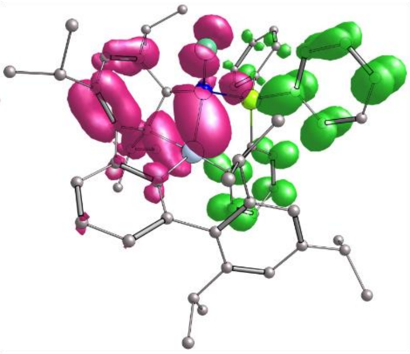

Excitation 8

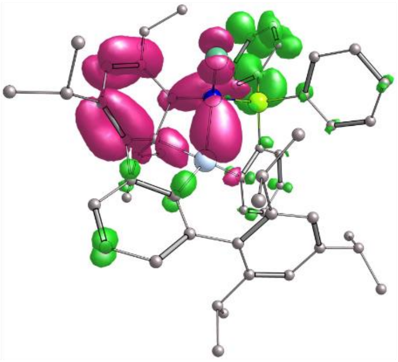

Excitation 9

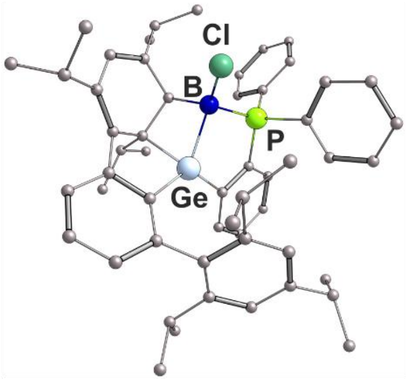

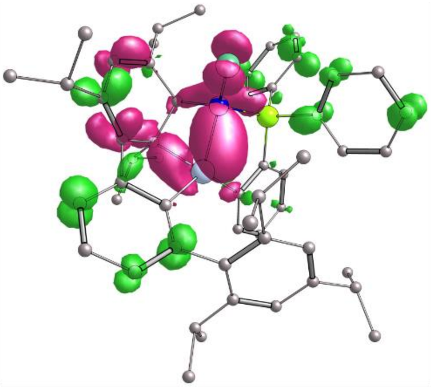

Excitation 10

Figure S 37. Density plots for Transitions in **6**.

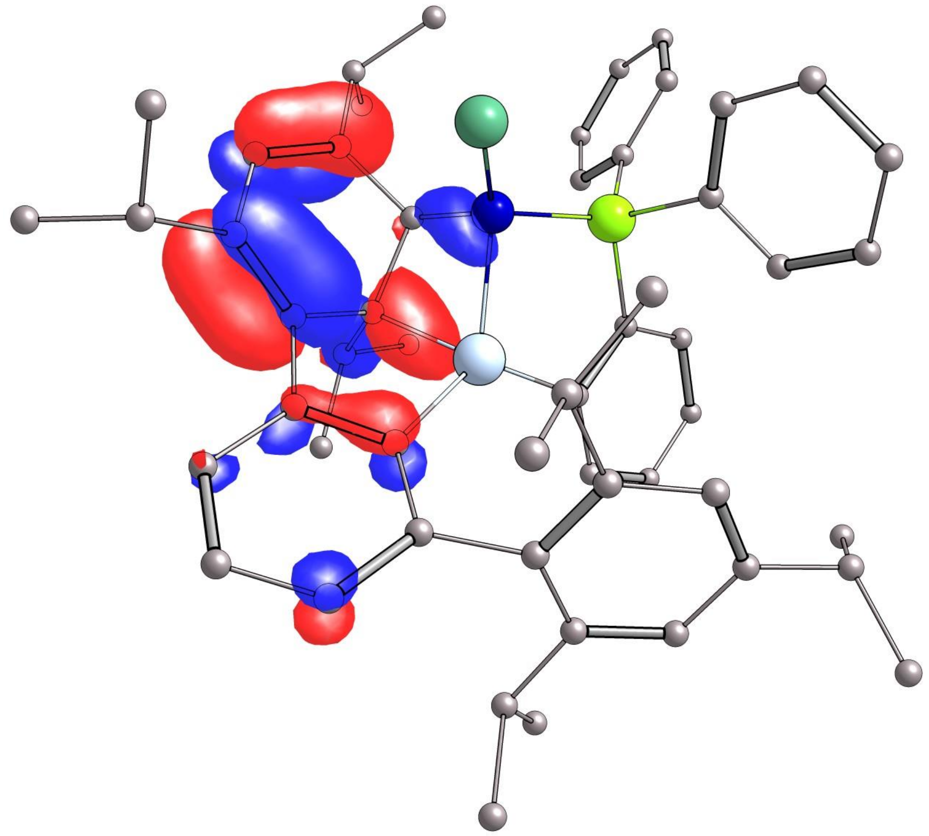

Figure S38. HOMO of 6.

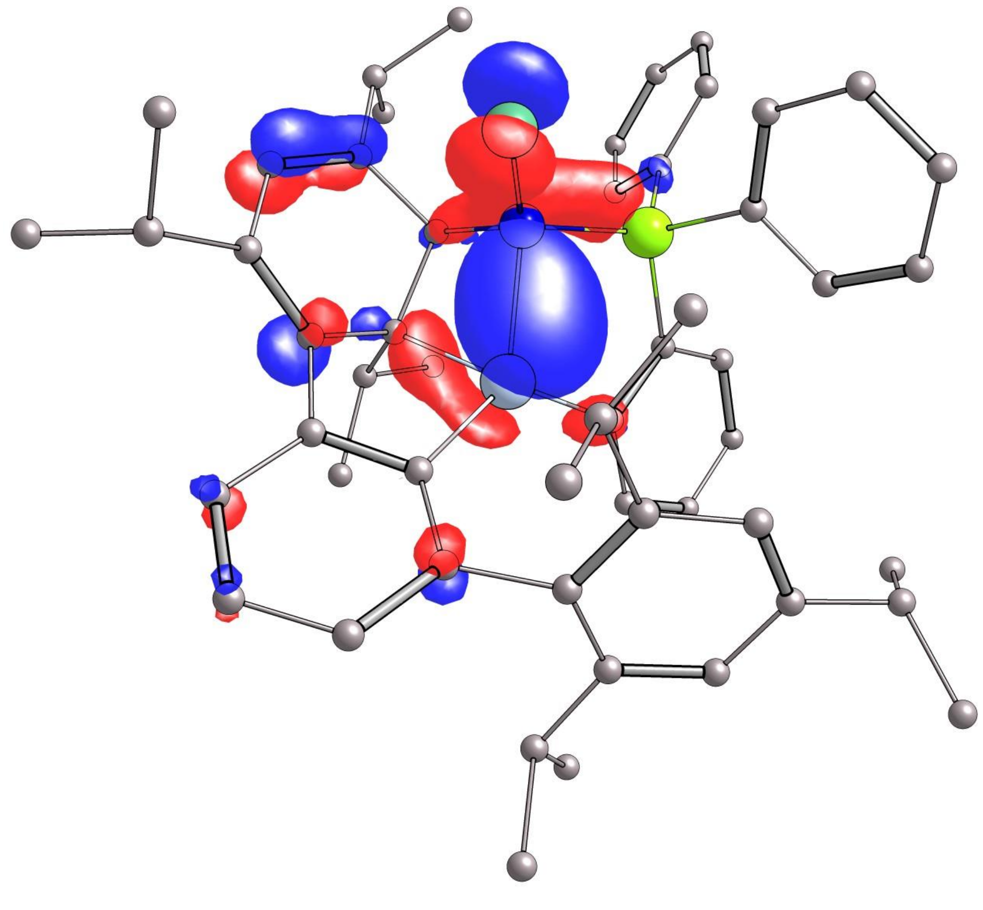

Figure S39. HOMO-1 of 6.

XYZ coordinates (4) (TDDFT calculations)

Ge	7.906855857	13.067492932	14.219285845
Cl	11.100508818	13.342815917	15.548873317
P	9.376094792	10.734642018	15.228931165
C	5.264767517	16.672510295	12.635664718
H	4.654662133	17.503528130	12.278306030
C	5.554697381	16.559131424	13.996671031
H	5.176325594	17.296939449	14.706127326
C	8.492570403	13.439100247	11.168301715
C	5.154683303	9.793862767	13.715554084
H	4.136938396	9.603993970	13.369045117
C	5.757967592	15.736169254	11.722441843
H	5.539603659	15.853027466	10.661370020
C	3.710744331	14.501098130	16.563618318
H	3.629863293	14.660558286	17.649452461
H	2.785347205	14.017439121	16.216029300
H	3.785736156	15.485179426	16.081384383
C	9.405853604	14.615110956	11.493331907
H	8.990247847	15.131853069	12.371021059
C	7.226084672	8.979813530	14.661716126
H	7.836207519	8.160078812	15.044898134
C	6.210912625	14.335483114	16.683836276
C	7.724070626	10.279750671	14.631553023
C	6.810183634	11.411476653	10.231473831
H	6.154465288	10.619961900	9.867583508
C	5.924549415	8.734954750	14.199278182
H	5.518574684	7.723403334	14.222846149
C	6.966945515	11.356748613	14.124981167
C	6.356395622	15.506477223	14.456438216
C	4.937911134	13.630181918	16.238860123
H	4.984340617	13.532902206	15.142581079
C	5.671639203	11.094955632	13.671606972
H	5.057645425	11.912175700	13.291807993
C	4.776199330	12.221345920	16.816819048
H	5.672737246	11.614326697	16.632933129
H	3.924745198	11.713705126	16.342608498
H	4.585719333	12.243888871	17.900275598
C	6.538498743	14.656392073	12.156539170
C	7.084155629	13.579385136	11.274954744
C	4.738204403	12.820052826	10.587046017
H	4.425371218	13.464707134	11.424458959
C	9.014213755	12.246436859	10.657257783
H	10.095561005	12.113549868	10.605568808
C	10.541370612	9.726891682	14.253846940
C	8.576411659	17.134227499	15.483467009
H	7.912398459	17.332784329	14.630100960
C	6.793805598	15.339396915	15.874543284
C	8.778223457	9.839236012	9.909688554
H	9.875309496	9.955398885	9.906976521
C	6.237532357	12.592519838	10.713318821
C	8.190665786	11.193678940	10.254248561
C	10.796585919	10.023454784	17.520092437

H	11.688641704	10.148186624	16.904980507
C	11.077390652	10.311634659	13.098673114
H	10.836151973	11.352694057	12.876725063
C	4.447363295	13.575980939	9.274267860
H	5.033438211	14.499926003	9.193943516
H	3.380048239	13.832589041	9.198287532
H	4.711449202	12.944242259	8.412731844
C	7.928911435	16.046131123	16.327124243
C	9.364541794	15.618239458	10.324518538
H	9.744688971	15.152657143	9.402667957
H	9.985947759	16.497905087	10.548867995
H	8.338179765	15.960700219	10.133725797
C	6.836558357	14.557461858	13.534728926
C	9.923840871	16.667200531	14.914509919
H	10.644025706	16.458222085	15.718165056
H	10.354482670	17.435593414	14.254946041
H	9.811448915	15.739187152	14.338667174
C	8.378979860	10.024207803	17.745891311
H	7.390462471	10.150293808	17.303747314
C	9.523231997	10.160217998	16.943573404
C	10.825555728	8.387871626	14.557760314
H	10.423141831	7.936789826	15.466022152
C	10.845735902	14.227989223	11.830941171
H	10.877752713	13.513969832	12.664913932
H	11.410602982	15.119185095	12.137922185
H	11.368011622	13.791679935	10.966030591
C	11.883350886	9.558298986	12.245150958
H	12.300763783	10.017191971	11.347925641
C	3.889725306	11.542436910	10.622616782
H	4.027710391	10.942838869	9.710641707
H	2.823003458	11.802369833	10.679719770
H	4.138420092	10.907097709	11.480360714
C	8.725017860	18.448085178	16.264837435
H	7.756058333	18.788744619	16.656704300
H	9.131954635	19.237168362	15.615218385
H	9.410263767	18.333783421	17.117764266
C	8.351204405	9.338868760	8.522558877
H	7.264102353	9.176263704	8.476256397
H	8.838793614	8.380804464	8.289163664
H	8.616800583	10.062330790	7.738843667
C	6.813218465	14.018348247	17.904737620
H	6.380019682	13.228207767	18.515721439
C	8.508966431	9.735207703	19.103415637
H	7.615852820	9.624831912	19.719751554
C	11.628910822	7.637025731	13.698429908
H	11.845722679	6.593655416	13.931863222
C	12.155510978	8.220176414	12.541253146
H	12.784352941	7.630140606	11.873062092
C	7.980586652	14.653110116	18.337599318
C	8.501444705	15.682043947	17.548702405
H	9.407723716	16.192713221	17.884388638
C	9.778146732	9.591032832	19.673816323
H	9.876756796	9.371216836	20.737441426

C	10.918697803	9.739205637	18.881282374
H	11.910223036	9.639805512	19.324763601
C	9.932739515	13.337689248	19.154504525
H	10.525213772	13.029754128	20.029106457
H	10.589431019	13.866877081	18.452047657
H	9.575613665	12.432862829	18.644293518
C	8.737506443	14.211369882	19.578147426
H	9.136678600	15.124169945	20.054802775
C	7.875486758	13.477949795	20.609835625
H	7.537413406	12.504672711	20.223496544
H	6.988203623	14.062775852	20.892021509
H	8.460179463	13.279494039	21.519488670
B	9.560572860	12.587529168	15.042508899
C	8.419346559	8.821809670	11.007320705
H	7.330848538	8.670062802	11.056832645
H	8.747741789	9.173720478	11.993630895
H	8.895355783	7.849410491	10.811819874

XYZ coordinates (6) (TDDFT calculations)

Ge	3.693239148	5.911391423	8.510308424
Cl	3.997769542	7.872035464	11.375892129
P	1.575083381	7.909116502	9.308049477
C	7.858340357	3.823249531	8.216918799
H	8.796432446	3.265876131	8.196683590
C	2.100568032	9.617331883	8.991327898
C	5.850730247	7.878517356	6.741404350
C	6.930479381	3.563485416	9.229870947
H	7.143094333	2.793665342	9.972057914
C	3.745811320	4.401591580	12.560886151
H	3.886087213	4.135662550	13.612281969
C	-0.096119756	7.960415473	10.024741743
C	4.771624592	8.511437688	4.639357268
C	2.342451248	6.320522696	7.159164388
C	5.998746415	6.553125731	6.257033597
C	7.613968420	4.788399511	7.232049558
H	8.369658403	4.993645141	6.473160698
C	-0.872894806	6.788840328	9.962440647
H	-0.523988137	5.934319614	9.381177540
C	6.428551585	8.281778467	8.093560703
H	6.205111255	7.477921712	8.810817600
C	4.877572228	4.226760413	11.654066850
C	4.995767141	7.220337807	4.154767436
H	4.665047236	6.965244739	3.146358274
C	2.892385474	11.748183141	9.834189778
H	3.154782247	12.389158080	10.676483851
C	-0.571062157	9.062329726	10.750314661
H	0.013193976	9.981303699	10.791634937
C	1.097724227	6.113915493	5.085527245
H	1.010339071	5.708246627	4.076163447
C	2.429141065	10.454573901	10.072119511
H	2.362810284	10.079795756	11.092515596
C	3.041684368	12.212978040	8.524456507
H	3.415484064	13.221514204	8.343224412

C	1.330143108	7.165755290	7.655740614
C	2.209670093	5.800298597	5.867160671
H	2.983792377	5.143433524	5.474376212
C	5.463048644	5.188725275	8.220083808
C	7.961970725	8.383714409	7.985436561
H	8.403131510	7.434272527	7.654578541
H	8.398860520	8.642661475	8.961190724
H	8.244568839	9.164119616	7.262275633
C	5.714206221	4.270011128	9.267585939
C	5.230035497	8.825546720	5.921141513
H	5.085203388	9.840426661	6.293429350
C	3.223718167	4.427107449	9.786132017
C	5.882236402	4.852574424	4.358455167
H	5.951942275	4.149996374	5.203531655
C	6.397639696	5.486926180	7.223686441
C	4.655751179	4.208459347	10.306234934
C	2.578491289	4.967981632	12.181749174
C	1.455384017	5.193352401	13.166590898
H	1.826493376	4.863791394	14.152423241
C	2.538936348	3.114279020	9.360577728
H	2.518440255	2.455622153	10.251323799
C	6.454145243	5.348763292	13.278565862
H	6.231167413	6.310006357	12.796071042
H	7.493933557	5.367550672	13.638284151
H	5.797620431	5.256654171	14.155767112
C	1.090752317	3.323727375	8.898709708
H	1.045834164	4.044441388	8.067248422
H	0.444122061	3.689381446	9.707836009
H	0.661392185	2.375447037	8.543183561
C	-1.795098348	8.987414632	11.416696987
H	-2.156316326	9.850315925	11.977625929
C	4.033709144	9.549404575	3.814135511
H	4.060055292	10.490151546	4.390701112
C	2.727649422	11.379409041	7.449274702
H	2.855216064	11.730493804	6.424543597
C	0.091188882	6.945165637	5.591356664
H	-0.778978015	7.187053616	4.980101378
C	6.255102629	4.186662536	12.291041209
H	6.997132431	4.316232115	11.490850066
C	-2.554050179	7.815732593	11.367370788
H	-3.506624262	7.758215788	11.895138838
C	-2.092997093	6.719803335	10.632404974
H	-2.685059120	5.805371290	10.580551953
C	0.203396155	7.472245757	6.878650703
H	-0.578864876	8.120462486	7.277137576
C	5.605274555	6.232971378	4.937991569
C	3.322789144	2.386982043	8.260967513
H	2.828123248	1.441333303	7.993863709
H	4.350936088	2.159122023	8.567992449
H	3.378534263	3.007047072	7.351507617
C	2.261900416	10.084763672	7.678833380
H	2.046128922	9.428838947	6.836976407
C	2.427358267	5.366191216	10.742842678

H	1.360627951	5.297669641	10.479582818
C	1.074213940	6.678804269	13.276595206
H	1.951497213	7.295264543	13.512190209
H	0.315977027	6.824889470	14.060314120
H	0.648472851	7.046714751	12.333980768
C	4.803126514	4.330075392	3.401222208
H	3.808460085	4.311404431	3.866191207
H	5.045189651	3.303920271	3.089416156
H	4.735866498	4.939703458	2.488081356
C	0.221630015	4.339722293	12.824258727
H	-0.216033966	4.648315181	11.863641679
H	-0.557241106	4.459407370	13.592415067
H	0.481984164	3.274182097	12.755537123
C	2.560144185	9.155032584	3.625086410
H	2.007715946	9.938237667	3.084363043
H	2.064916302	8.983018756	4.590450178
H	2.477987300	8.221753891	3.048403332
C	6.518781598	2.829432961	12.963512249
H	5.794281322	2.639826107	13.770227090
H	7.526430332	2.801496107	13.405291113
H	6.433100393	2.004674235	12.241201179
C	5.844786528	9.572384626	8.669145738
H	6.120819669	10.453231907	8.069491605
H	6.227248327	9.729654517	9.686394806
H	4.751653443	9.526067858	8.733852474
C	7.246127789	4.861170379	3.641246425
H	7.506605765	3.854612279	3.281093152
H	8.047060839	5.208330234	4.306576217
H	7.216854762	5.540048167	2.775285958
C	4.714875624	9.809526943	2.462249039
H	4.703192449	8.906116602	1.834456964
H	5.763261481	10.109020628	2.598860380
H	4.195379144	10.606248801	1.909260931
B	2.947797212	6.867442729	10.255971563

References

[1] a) R. S. Simons, L. Pu, M. M. Olmstead, P. P. Power, *Organometallics* **1997**, *16*, 1920-1925; b) S. Akiyama, K. Yamada, M. Yamashita, *Angew. Chem. Int. Ed.* **2019**, *58*, 11806-11810; c) J. Schneider, K. M. Krebs, S. Freitag, K. Eichele, H. Schubert, L. Wesemann, *Chem.-Eur. J.* **2016**, *22*, 9812-9826.

[2] R. K. Harris, E. D. Becker, S. M. Cabral de Menezes, R. Goodfellow, P. Granger, *Pure Appl. Chem.* **2001**, *73*, 1795-1818.

[3] a) L. J. Farrugia, *J. Appl. Crystallogr.* **1999**, *32*, 837-838; b) C. B. Hübschle, G. M. Sheldrick, B. Dittrich, *J. Appl. Crystallogr.* **2011**, *44*, 1281-1284; c) G. Sheldrick, *Acta Cryst., Sect. A* **2008**, *64*, 112-122; d) *Bruker AXS Inc. Madison, Wisconsin, USA, 2007* **2007**; e) G. Sheldrick, *SADABS, University of Göttingen, Germany, 2008*.

[4] M. J. Frisch, G. W. Trucks, H. B. Schlegel, G. E. Scuseria, M. A. Robb, J. R. Cheeseman, G. Scalmani, V. Barone, B. Mennucci, G. A. Petersson, H. Nakatsuji, M. Caricato, X. Li, H. P. Hratchian, A. F. Izmaylov, J. Bloino, G. Zheng, J. L. Sonnenberg, M. Hada, M. Ehara, K. Toyota, R. Fukuda, J. Hasegawa, M. Ishida, T. Nakajima, Y. Honda, O. Kitao, H. Nakai, T. Vreven, J. J. A.

Montgomery, J. E. Peralta, F. Ogliaro, M. Bearpark, J. J. Heyd, E. Brothers, K. N. Kudin, V. N. Staroverov, R. Kobayashi, J. Normand, K. Raghavachari, A. Rendell, J. C. Burant, S. S. Iyengar, J. Tomasi, M. Cossi, N. Rega, J. M. Millam, M. Klene, J. E. Knox, J. B. Cross, V. Bakken, C. Adamo, J. Jaramillo, R. Gomperts, R. E. Stratmann, O. Yazyev, A. J. Austin, R. Cammi, C. Pomelli, J. W. Ochterski, R. L. Martin, K. Morokuma, V. G. Zakrzewski, G. A. Voth, P. Salvador, J. J. D. S. Dapprich, A. D. Daniels, Ö. Farkas, J. B. Foresman, J. V. Ortiz, J. Cioslowski, D. J. Fox, *Gaussian 09, Revision D.01, Gaussian, Inc., Wallingford CT* **2009**.

[5] a) J. P. Perdew, *Phys. Rev. B* **1986**, *33*, 8822-8824; b) A. D. Becke, *Phys. Rev. A* **1988**, *38*, 3098-3100; c) F. Weigend, R. Ahlrichs, *Phys. Chem. Chem. Phys.* **2005**, *7*, 3297-3305; d) F. Weigend, *Phys. Chem. Chem. Phys.* **2006**, *8*, 1057-1065; e) D. Andrae, U. Häussermann, M. Dolg, H. Stoll, H. Preuß, *Theoret. Chim. Acta* **1990**, *77*, 123-141; f) A. Bergner, M. Dolg, W. Küchle, H. Stoll, H. Preuß, *Mol. Phys.* **1993**, *80*, 1431-1441; g) R. Krishnan, J. S. Binkley, R. Seeger, J. A. Pople, *J. Chem. Phys.* **1980**, *72*, 650-654; h) A. D. McLean, G. S. Chandler, *J. Chem. Phys.* **1980**, *72*, 5639-5648.

[6] S. Grimme, S. Ehrlich, L. Goerigk, *J. Comput. Chem.* **2011**, *32*, 1456-1465.

[7] G. A. Zhurko, CHEMCRAFT (http://www.chemcraftprog.com).

[8] a) E. D. Glendening, C. R. Landis, F. Weinhold, *J. Comput. Chem.* **2013**, *34*, 1429-1437; b) E. D. Glendening, J. K. Badenhoop, A. E. Reed, J. E. Carpenter, J. A. Bohmann, C. M. Morales, C. R. Landis, F. Weinhold, *NBO 6, Theoretical Chemistry Institute, University of Wisconsin, Madison* **2013**; c) A. E. Reed, R. B. Weinstock, F. Weinhold, *J. Chem. Phys.* **1985**, *83*, 735-746.

[9] E. D. Glendening, J. K. Badenhoop, A. E. Reed, J. E. Carpenter, J. A. Bohmann, C. M. Morales, P. Karafiloglou, C. R. Landis, F. Weinhold, Theoretical Chemical Institute, University of Wisconsin Madison, 2018.

[10] J. P. Perdew, K. Burke, M. Ernzerhof, *Phys. Rev. Lett.* **1996**, *77*, 3865-3868.

[11] J. P. Perdew, W. Yue, *Phys. Rev. B* **1986**, *33*, 8800-8802.

[12] a) F. Neese, *Wiley Interdiscip. Rev. Comput. Mol. Sci.* **2012**, *2*, 73-78; b) F. Neese, *Wiley Interdiscip. Rev. Comput. Mol. Sci.* **2018**, *8*, e1327.

[13] T. Yanai, D. P. Tew, N. C. Handy, *Chem. Phys. Lett.* **2004**, *393*, 51-57.

[14] F. Neese, F. Wennmohs, A. Hansen, U. Becker, *Chem. Phys.* **2009**, *356*, 98-109.

[15] G. A. Zhurko, Version 1.7 (build132) ed., **2014**.
